# Consequences of aberrated DNA methylation in Colon Adenocarcinoma: a bioinformatic-based multi-approach

**DOI:** 10.1186/s12863-022-01100-7

**Published:** 2022-11-29

**Authors:** Arash Moradi, Milad Shahsavari, Erfan Gowdini, Kamal Mohammadian, Aida Alizamir, Mohammad Khalilollahi, Zahara Mohammadi Abgarmi, Shahla Mohammad Ganji

**Affiliations:** 1 Department of Medical Biotechnology, National Institute of Genetic Engineering and Biotechnology (NIGEB), Shahrak-E Pajoohesh, Km 15, P.O. Box 14965/161, Tehran - Karaj Highway, Tehran, Iran; 2 Department of Biology, Faculty of Biological Sciences, North Tehran Branch, Islamic Azad University, Tehran, Iran; 3 Department of Radiation Oncology, Hamadan University of Medical Sciences, Mahdieh Center, Hamadan, Iran; 4 Department of Pathology, Hamadan University of Medical Sciences, Hamadan, Iran; 5 Department of Microbiology, Islamic Azad University, North Tehran Branch, Tehran, Iran; 6 Department of Clinical Biochemistry, Faculty of Medical Science, Tarbiat Modares University, Tehran, Iran

**Keywords:** DNA Methylation, Colorectal Neoplasms, Epigenetic Biomarkers, Precision Cancer Medicine

## Abstract

**Introduction:**

The biology of colorectal cancer (CRC) is remained to be elucidated. Numerous genetic and epigenetic modifications are in concert to create and progress CRC. DNA methylation as a principal epigenetic factor has gained increased attention and could be utilized for biological studies. This study aims to find novel methylated and downregulated genes with a focus on HAND2 in CRC and decipher the biological consequences.

**Material and method:**

Data on DNA methylation from GEO and SMART databases and the expression GEPIA2 database were downloaded. Afterward, a set of hypermethylated and downregulated genes in CRC was chosen by overlapping genes. Consequently, *HAND2* was selected as a key gene for further investigation and confirmed with cell lines methylation and expression data. The functions of HAND2 were further analyzed using gene ontology analyses and the protein–protein interaction network.

**Results:**

The methylation (*p* < 0.01) and expression (*p* < 0.01) of *HAND2* are significantly varied in CRC compared to normal control. The correlation analysis (Pearson's correlation coefficient = -0.44, *p* = 6.6e-14) conveys that HAND2 significantly downregulated and has a reverse correlation with the methylation status of CpG islands. The biological process analysis of HAND2 target genes conveyed that disruption in HAND2 expression could dysregulate ERK1 and ERK2 signaling pathways.

**Conclusion:**

Together, the findings showed that DNA hypermethylation of *HAND2* was critical evidence in CRC. Further validation and prospective studies are needed to utilize *HAND2* methylation as a promising biomarker.

**Supplementary Information:**

The online version contains supplementary material available at 10.1186/s12863-022-01100-7.

## Highlights

• Multiple open-access datasets were investigated.

• Investigation of cell lines methylation and expression data were added to consolidate the tissue-based data.

• The focus of this study was on the consequence of aberrant DNA methylation.

• The study introduced a simple method for investigating online databases.

**Supplementary Information:**

The online version contains supplementary material available at 10.1186/s12863-022-01100-7.

## Introduction

Colorectal cancer (CRC) is one of the leading causes of cancer-related death, and numerous efforts have been made to understand the accurate biology of CRC. In 2021 Sung et al*.* reported that CRC ranks third in incidence and second in mortality. In addition, they claimed that "CRC can be considered a marker of socioeconomic development, and, in countries undergoing a major transition, incidence rates tend to rise uniformly with increasing Human Development Index (HDI)" [1]. Recently, oncologists have been focused on the precision cancer medicine (PCM) concept, which utilizes targeted therapies to obtain efficient treatment with less inconvenience that patients might face [2]. The researcher's efforts elucidate numerous aspects of cancer biology and improve the quality of patient outcomes; nevertheless, cancer-related morbidity and mortality rate remain prevalent.

CRC progression arose from genetic and epigenetic alterations. The first profound model of CRC progression was proposed by Fearon and Vogelstein [3], which was based on the accumulation of genetic alterations. Epigenetic alterations can be exploited as clinically relevant disease biomarkers for diagnosis, prognostication, and treatment response prediction; they may also be targeted in novel therapies. The major epigenetic regulators are DNA methylation, histone modifications, and non-coding RNA species [4]. Furthermore, investigations revealed that epigenetic aberrations could perturb gene expression and lead to malignant transformation. It is also suggested that aberrant epigenetic modifications probably occur early in pathogenesis and are in concert with genetic defections [5]. Intriguingly, one of the unique properties of epigenetic alterations is that they are reversible, and it has been shown that they have treatment value in various cancers [6]. Thus, the impact of epigenetic alterations on cancer progression should be emphasized and studied more.

DNA methylation is an enzymatic modification in that DNA methyltransferases add a methyl group to cytosines leading to the regulation of DNA–protein interactions in the major grooves. Mainly, aberrant DNA methylation plays a significant role in tumorigenesis. Hypomethylation is commonly observed during cancer progression, leading to genomic instability and, less frequently, oncogenes' activation. DNA hypomethylation occurs on specific sequences, such as heterochromatic DNA repeats, dispersed retrotransposons, and endogenous retroviral elements. On the other hand, hypermethylation could suppress the tumor suppressor genes and has often been observed in CpG islands in gene regions. CpG island methylator phenotype (CIMP) is the hypermethylation of cancer-specific CpG islands [7]; Toyota et al*.* suggested CIMP as a subset of CRC [8], and they proposed that deciphering the mechanisms underlying methylation phenomena can be beneficial for CRC precise detection, prevention of cancer progression, and development of novel therapies.

This study aimed to explore the consequences of aberrated DNA methylation in CRC patients. The cancer methylome, for instance, CpG island hypermethylation, is traceable evidence. This study provides a multi-approach bioinformatics analysis strategy for identifying the hypermethylated and downregulated genes. Datasets (GSE17648, GSE25062, GSE29490, GSE47071, and GSE47592) from the publicly available Gene Expression Omnibus (GEO) database were downloaded and analyzed by GEO2R. Also, the TCGA differentially methylated CpGs and the expression data were obtained from the SMART GEPIA2 databases, respectively.

**Fig. 1:**
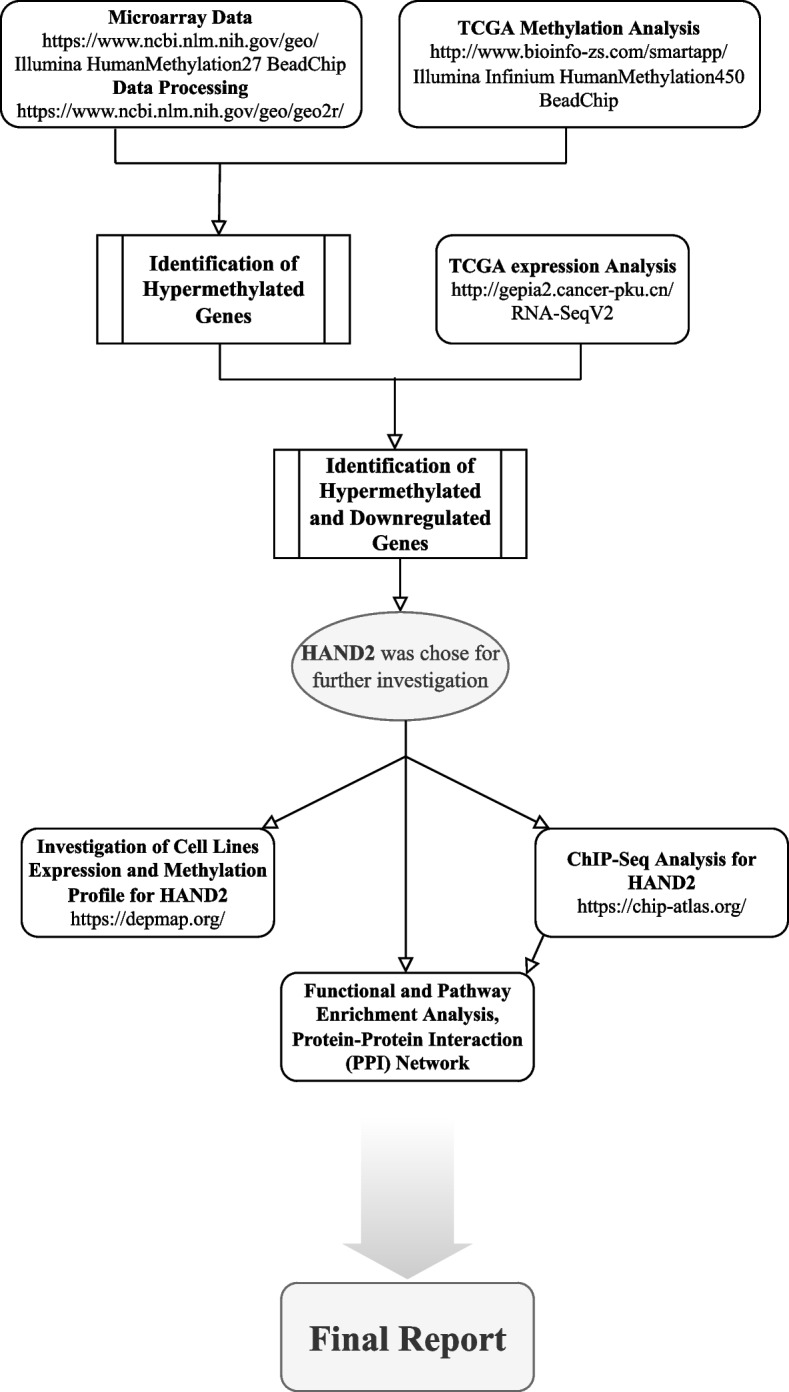
Flowchart of the bioinformatic analysis

Consequently, *HAND2* was selected as a key factor for further investigation. This approach was followed by chip-seq analysis, gene ontology (GO) enrichment analyses, and protein–protein interaction networks. The concise path of this study is depicted in Fig. 1.

## Material and methods

### Databases analysis

#### GEO database

The colorectal cancer tissue methylation profile datasets were obtained from the NCBI GEO database (http:// www.ncbi.nlm.nih.gov/geo/). The accession number was GSE17648, GSE25062, GSE29490, GSE47071, and GSE47592. The microarray data of GSE17648 was based on GPL8490 Platforms (Illumina HumanMethylation27 BeadChip), including 22 tumoral and 22 adjacent normal samples; GSE25062 was based on GPL8490 Platforms (Illumina HumanMethylation27 BeadChip), including 125 tumoral and 29 adjacent normal samples; GSE29490 was based on GPL8490 Platforms (Illumina HumanMethylation27 BeadChip), including 24 tumoral and 24 adjacent normal samples; GSE47071 was based on GPL8490 Platforms (Illumina HumanMethylation27 BeadChip), including 51 tumoral and 38 adjacent normal samples; and GSE47592 was based on GPL8490 Platforms (Illumina HumanMethylation27 BeadChip), including 51 tumoral and 38 adjacent normal samples. Differentially methylated CpGs were refined _log2_FC higher than 1 and p-value less than 0.01. It should be mentioned that _log2_FC is relative to the group selection order and only available when two groups of samples are defined. In this study, we defined two groups for each dataset, including "Tumoral" and "Normal," respectively. Hence, _log2_FC for hypermethylated regions was positive. The duplicated gene's name was deleted. The instruction of GEO2R explained that the _log2_FC is based on M-value and adjusted *p*-value calculated through Benjamini & Hochberg [9].

#### Investigating the methylation by SMART database

The methylation data based on Illumina Infinium HumanMethylation450 BeadChip from the SMART database (http://www.bioinfo-zs.com/smartapp/), including 288 cancerous and 34 normal samples (COAD (Colon adenocarcinoma)), were downloaded. Differentially methylated CpGs were refined through the M-value higher than 2 and p-value less than 0.01 [10]. The duplicated gene's name in the obtained results from the SMART database was deleted to have a faster and more reliable analysis. This study considers the M-value metric to measure methylation levels because the M-value is more statistically valid for the differential analysis of methylation levels [11].

#### Investigating the gene expression by GEPIA2

The expression data is based on the RNA-SeqV2 platform, generated by the GEPIA2 database (http://GEPIA2.cancer-pku.cn/). The gene expression profile of COAD is provided by Genotype-Tissue Expression (GTEx) and TCGA repository. Two hundred seventy-fine COAD cancerous samples and three hundred forty-nine normal samples, including 41 COAD normal adjacent samples, and 308 GTEx-based samples, were analyzed. Differentially expressed genes were refined through _Log2_FC cutoff higher than 1, q-value cutoff less than 0.01, and under-expressed as the chromosomal distribution by ANOVA analysis. The q-value is an adjusted p-value, considering the false discovery rate (FDR) [12].

#### The procedure of hypermethylated and downregulated genes discovery

Firstly, hypermethylated genes (_log2_FC less than 1 and p-value less than 0.01) in the five GEO datasets, including GSE17648, GSE25062, GSE29490, GSE47071, and GSE47592, were analyzed by Venn diagram (http://bioinformatics.psb.ugent.be/webtools/Venn/) to find intersections (hypermethylated genes) among five datasets. Afterward, hypermethylated genes (M-value higher than 2 and p-value less than 0.01) obtained from the SMART database were compared to the results of the previous GEO datasets intersections analysis. Ultimately, another Venn diagram was constructed to find the downregulated genes (_Log2_FC cutoff higher than 1, q-value cutoff less than 0.01) intersections with obtained hypermethylated genes from the previous step.

### Investigation of cell lines expression and methylation profile

The DepMap database (https://depmap.org/) was used to confirm the tissue's expression and methylation profile. This profound database provides "discoveries related to cancer vulnerabilities by providing open access to key cancer dependencies analytical and visualization tools" is provided. For this purpose, fifty-one colorectal cancer cell lines with methylation and expression data were chosen. The predefined expression and methylation data are based on _log2_(TPM + 1) and Beta-value, respectively.

### ChIP-Seq analysis for HAND2

This study uses the ChIP-Atlas public database (https://chip-atlas.org/), which analyses ChIP-Seq, DNase-Seq, ATAC-Seq, and Bisulfite-Seq data to find targets of the selected transcription factor. An accurate relationship between transcription factors (TFs) and their target genes and the effect of their regulatory activity (activator or repressor) should be established to define a distinct transcriptional regulatory network. The parameter for this analysis is refined through the average score based on Model-based Analysis of ChIP-Seq (MACS) above 499 and the ± 1 kb distance from the transcription start site (TSS) [13, 14].

### Functional and pathway enrichment analysis, protein–protein interaction (PPI) network

Gene ontology analysis (GO) is a proper standard method for annotating genes for identifying biological processes (BP), cellular components (CC), and molecular function (MF). In order to analyze the selected genes for functional enrichment, GO enrichment and KEGG pathway analysis were performed using ShinyGO (http://bioinformatics.sdstate.edu/go/). Furthermore, the STRING database (https://string-db.org) was used for protein–protein interaction (PPI) analysis to investigate the molecular mechanisms.

### Statistical analyses

In this study, the methylation levels were measured based on the M-value. The M-value method performs efficiently in Detection Rate (DR) and True Positive Rate (TPR) for both highly methylated and unmethylated CpG sites [11]. Correlation analysis was performed using Pearson's correlation to measure the strength of the linear relationship between two variables [15]. It should be declared to unify the data, and this study utilized the COAD data for investigation.

## Results

### Identification of DNA differentially methylated regions and differentially expressed genes in colon cancer tissues and cell lines

**Fig. 2:**
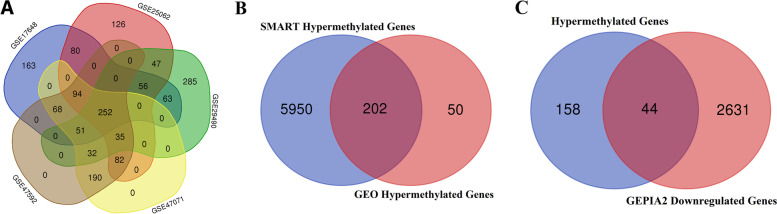
Multistep Venn Diagram for Obtaining Hypermethylated and Downregulated Genes. **A** The Venn diagram among five GEO methylation datasets. **B** The Venn diagram between the result of hypermethylated GEO genes and the SMART database hypermethylated genes. **C** The Venn diagram of hypermethylated genes and GEPIA2 downregulated genes

Five datasets (GSE17648, GSE25062, GSE29490, GSE47071, and GSE47592), including 272 tumoral samples and 151 normal controls, were downloaded to identify differentially methylated regions. The hypermethylated regions were indicated by generating a Venn diagram of five datasets. Altogether, two hundred and fifty-two common hypermethylated regions were defined (Fig. 2-A, Supplement 1).

Afterward, the common hypermethylated genes obtained from the GEO database were compared to SMART database hypermethylated genes through another Venn diagram. Two hundred two significant hypermethylated regions (genes) were obtained (Fig. 2-B, Supplement 2).

Ultimately, hypermethylated genes were compared to the significantly downregulated genes to identify the hypermethylated and downregulated genes simultaneously. *HPSE2, SDC2, SPG20, RSPO2, ZNF667, SFRP2, CHST10, HAND2, NPY, ZNF677, FIGN, GPM6A, AMPH, D4S234E, ADHFE1, CNTN1, TRPC6, GRIK3, NRXN3, GFRA1, FLT4, JAM3, UCHL1, ATP8B2, MAL, CNR1, THBD, PHOX2A, EDNRB, KIF5A, NPR3, SOX17, NTRK3, VIPR2, CD34, GRASP, CDO1, INA, JAM2, RYR2, GAS7, PDE8B, SFRP1, and PRSS1* were significantly hypermethylated and downregulated in COAD samples (Fig. 2-C, Supplement 3). The *HAND2* gene was selected in this study for further investigation because the potential role of HAND2 in CRC is not well understood.

### The correlation of *HAND2* methylation with its expression

Correlation analysis is a common method to examine the relationship between specific gene methylation and expression [16]. In this study, Pearson's correlation was calculated between the *HAND2* methylation and expression. Interestingly, the aggregated (mean of all probes) Pearson's correlation (Pearson's correlation coefficient = -0.44, *p* = 6.6e-14 for COAD) conveyed that *HAND2* significantly downregulated in CRC (288 colon cancer) and has a reverse correlation with the methylation status of CpG islands (Supplementary Fig. 1).

### The *HAND2* methylation and expression in CRC cell lines

The DepMap database was investigated to consolidate the correlation between *HAND2* methylation and expression hypothesis. Interestingly, the data available on the DepMap database (https://depmap.org/) for cancerous cell lines conveyed that *HAND2* is hypermethylated in CRC cell lines, simultaneously downregulated. Furthermore, the Pearson correlation coefficient test revealed a negative correlation (Pearson's correlation coefficient = -0.3035, *p* = 0.030) between the *HADN2* expression and methylation (Supplement 4, Supplementary Fig. 3).

### Identifying the HAND2 downstream genes, signaling pathways, and the interaction with other proteins

**Fig. 3:**
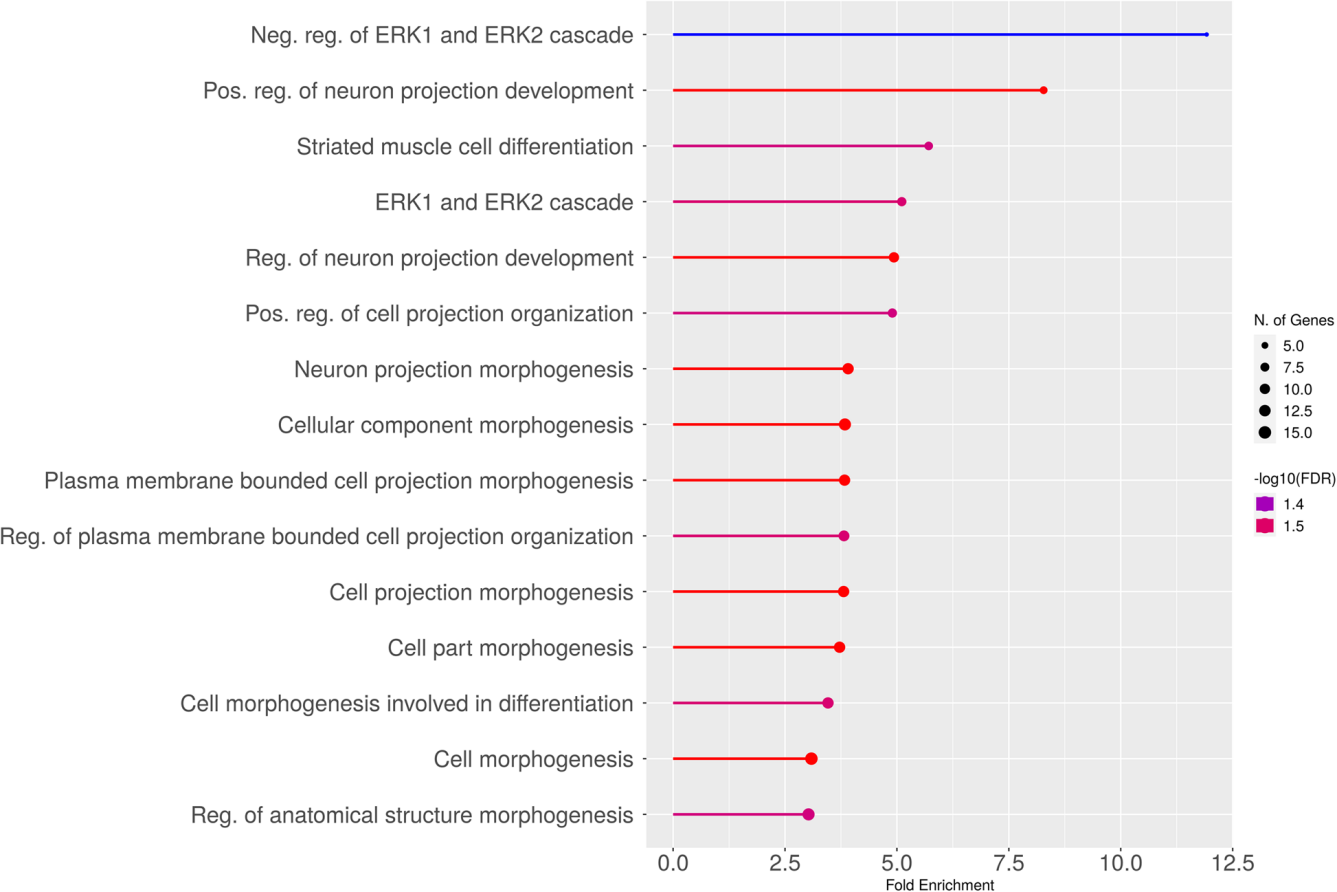
The interactive biological process of the HAND2 target genes. This figure shows that dysregulated expression of *HAND2* could have a mal-impact on cell homeostasis, for instance, the ERK1/2 cascade

Using the ChIP-Atlas database, potential HAND2 targets were identified by an average score above 499 and the ± 1 kb distance from the transcription start site (TSS). The number of refined target genes was 104. Afterward, obtained genes were analyzed with ShinyGO for enrichment analysis (Fig. 3 and Supplement 5).

The final results of the biological process of HAND2 target genes conveyed that disruption in HAND2 expression could dysregulate ERK1 and ERK2 signaling pathways. Notably, the HAND2 downstream genes conveyed that HAND2 is a critical transcription factor for maintaining cell homeostasis. Interestingly, it was shown that HAND2 could directly bind to ERK and reduce the phosphorylation of ERK [17]. In this study, the results showed that HAND2 downstream genes could regulate ERK1 and ERK2 cascade.

**Fig. 4:**
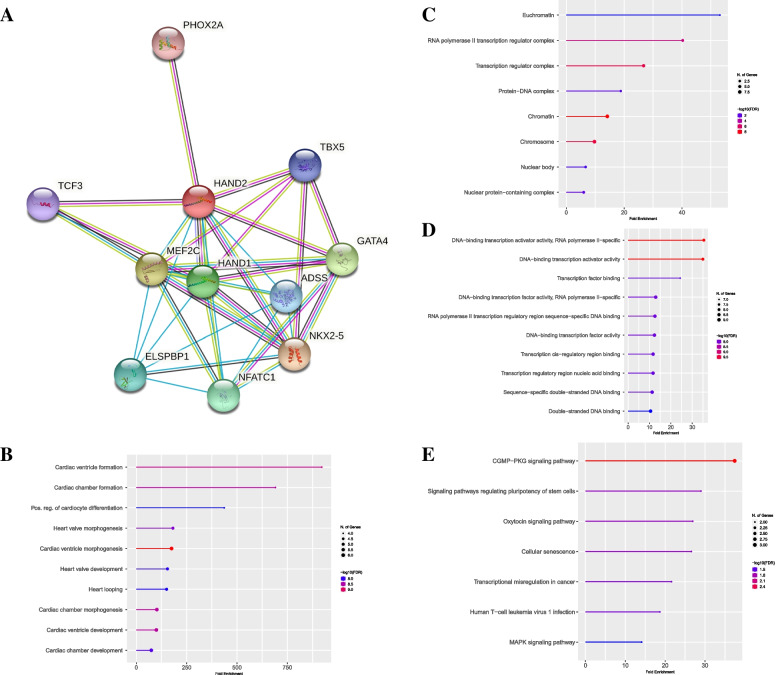
The HAND2 interactions and Gene Ontology. **A** The protein–protein Interaction network of HAND2. Gene Ontologies are represented as general function categories (**B**) biological process, (**C**) cellular component, (**D**) molecular function, and (**E**) KEGG

On the other hand, by utilizing the String database, the interaction network of HAND2 revealed that it has numerous potential interactions with critical proteins, including ADSS, ELSPBP1, GATA4, HAND1, MEF2C, NFATC1, NKX2-5, TBX5, TCF3, and PHOX2A, which all of them are capable of binding DNA. Pathway enrichment analysis (KEGG) and functional enrichment analysis (GO) were applied to elucidate the biological functions of the putative interaction proteins related to HAND2. Enriched results were subjected to multiple testing adjustments with a threshold value FDR (q-value) less than 0.05. To better exhibit functional consequence, only the top twenty significant enriched GO terms are shown in Fig. 4. The BP enrichment analysis manifests that HAND2 misregulation could perturb Cardiac ventricle morphogenesis (FDR = 3.22E-10), Cardiac ventricle formation (FDR = 1.77E-09), and Cardiac chamber morphogenesis (FDR = 2.61E-09). The KEGG enrichment analysis reveals that the misregulation of HAND2 could impact the CGMP-PKG signaling pathway, Cellular senescence, and Signaling pathways regulating the pluripotency of stem cells.

### The expression pattern of HAND2 antisense1 long non-coding RNA and its correlation with CpG island methylation

**Fig. 5:**
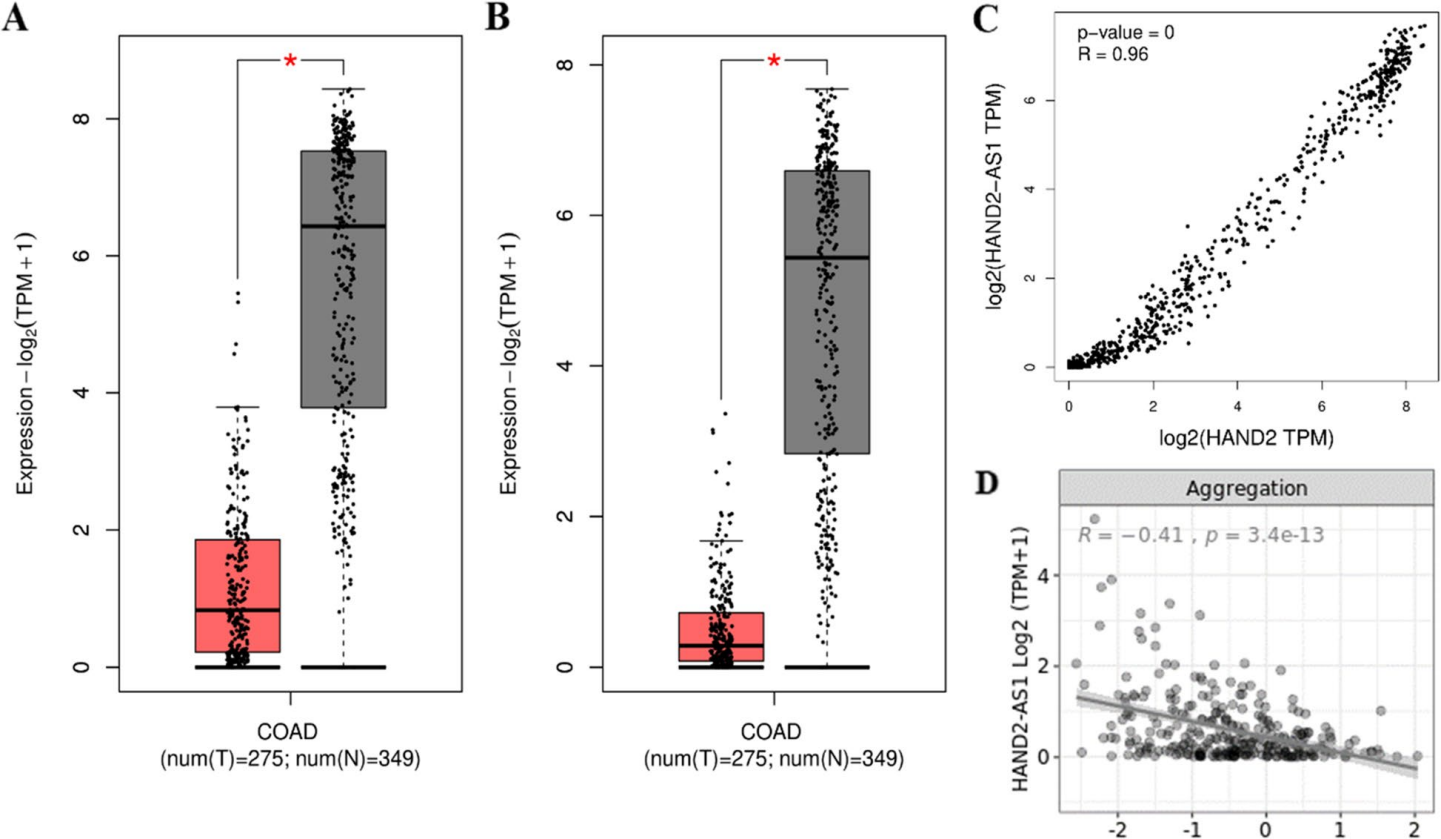
The Expression of *HAND2* and HAND2-AS1 in COAD samples. The plots are depicted by the GEPIA2 database. **A** *HAND2* and (**B**) HAND2-AS1 expression COAD samples. Red boxes are for tumoral samples, and gray boxes are for normal samples. **C** Pearson's correlation between *HAND2* and HAND2-AS1 expression. **D** The correlation between HAND2-AS1 expression and CpG island methylation

Previous investigations revealed that *HAND2* has an antisense long non-coding RNA [18]. The expression data of 275 COAD and 308 normal samples, analyzed by the GEPIA2, revealed that the *HAND2* and HAND2-AS1 were significantly downregulated in COAD samples compared to normal samples. The p-value cutoff was set to less than 0.01. (Fig. 5-A and Fig. 5-B) Afterward, the Pearson's correlation test revealed that the expression of *HAND2* and its long non-coding RNA antisense, HAND2-AS1, are positively correlated (Pearson's correlation coefficient = 0.96, *p* < 0.001) (Fig. 5-C).

Another Pearson's correlation test revealed that the expression of HAND2-AS1 had a significant (Pearson's correlation coefficient = -0.41, *p* = 3.4e-13 for COAD) reverse correlation with the methylation status of CpG islands (mean of all probes) (Fig. 5-D, Supplementary Fig. 2). This evidence led to deduced that HAND2-AS1 could be under the control of DNA methylation, which hypermethylation of CpG islands affects the expression of HAND2-AS1. Aligning with our hypothesis, previous studies on ovarian carcinoma [19] and endometrioid endometrial carcinoma [20] conveyed that HAND2-AS1 expression could negatively correlate with its promoter CpG island methylation.

## Discussion

Multiple lines of evidence proved that most of the CpG contents of DNA (approximately 70%) in vertebrates are located in the promoters, called CpG islands; nevertheless, CpG density itself does not influence gene expression, and the regulation of gene expression is dependent on the methylation of cytosine contents [21]. In general, DNA methylations are recognized by methyl-CpG binding domain proteins (MBDs), leading to recruits of histone deacetylase and gene silencing [22]. Studies demonstrated that cancer cells have a different methylome profile compared to normal cells. Current hypotheses are proposed that epigenetic disruptions are starting the processes of cancer creation [23]. Consequently, the studies focusing on epigenetic alterations are beneficial to understanding cancer's biology more precisely.

This study utilized a multifaceted approach to assess the consequence of DNA methylation in colorectal cancer. The statistical population for studying DNA methylation consists of 273 samples for cancerous tissues and 181 for normal controls, which were analyzed from different GEO datasets. Another resource for analyzing DNA methylation data was the SMART database, which includes 288 cancerous and 34 normal COAD samples. Furthermore, the gene expression data of 275 samples of cancerous tissues and 349 normal controls, which were analyzed by the GEPIA2 database, was used in this study. Afterward, multistep Venn diagrams were constructed to reveal the intersections between the hypermethylated and the downregulated genes. HAND2 is selected as an eligible candidate for further investigations because the role of HAND2 in CRC is not well understood.

HAND2 is a basic helix-loop-helix (bHLH) protein that forms homo- or hetero-dimers with other bHLH partners, such as HAND1. The constructed dimers could regulate gene expression by binding to enhancer boxes (E-boxes) [24]. HAND2 is well-known for its function in myocardial differentiation and is suggested to regulate the establishment of myocardial epithelial identity concomitant with GATA5 [25]. Multiple lines of evidence conveyed the aberrant expression and hypermethylation of HAND2 in various cancers. Prummel et al*.* expressed that loss of HAND2 disrupted mesothelium formation with reduced progenitor cells and perturbed migration, which leads to mesothelioma tumor formation [26]. In addition, the methylation pattern of the *HAND2* gene was investigated in endometrial cancer, and it was revealed that the alterations in *HAND2* DNA methylation commonly occur in endometrial cancer and could be utilized as a biomarker for early detection and a predictor of treatment response [27]. Also, aberrated *HAND2* DNA methylation was observed in cervical cancer [28]. This evidence expounds the critical role of *HAND2* silencing in cancer initiations.

In this study, different methylation and expression data for COAD were downloaded from different databases. *HAND2* downregulation and hypermethylation were commonly observed in COAD. Pearson's correlation conveyed that *HAND2* significantly (R = -0.44, *p* = 6.6e-14) hypermethylated and downregulated in the TCGA COAD samples. Also, the data obtained from the DepMap database shows a significant negative correlation (Pearson's Correlation Coefficient = -0.3035, *p* = 0.030) between the *HAND2* methylation and expression in colorectal cancer cell line data. A recent study conveyed that *HAND2* hypermethylation in CRC occurred more prevalently than other classic alterations. It was proved that HAND2 methylation is relevant to gene silencing [17]. Also, a pan-cancer analysis using TCGA data proved that methylation-induced gene expression silencing has developed across all thirty-three cancer types [29]. It could be deduced from previous studies that *HAND2* methylation may be crucial in early carcinogenesis, not only a dull epigenetic event. However, it is suggested that the exact mechanism should be investigated.

Another notable finding of this study expressed that downstream genes of HAND2, including DAB2IP, EMILIN1, CHRNA9, and DMD, are pivotal in regulating ERK1/2 signaling. Multiple lines of evidence demonstrated that ERK1/2 misregulation is fundamental for the development and progression of cancer [30]. ERK1/2 signaling can regulate BCL-2 proteins [31], which regulate numerous vital processes such as cell cycle progression, migration, and survival dysregulation that are cancer's hallmarks [32]. The positive correlation of HAND2 downregulation with MAPK/ERK signaling perturbation was reported recently [17]. Furthermore, this study showed that HAND2 could indirectly regulate the ERK1/2 cascade through its downstream target genes. Accordingly, the suppression of HAND2 may be implicated in the misregulation of ERK1/2 signaling.

Meanwhile, *HAND2* has an antisense long non-coding RNA that is downregulated in CRC. HAND2-AS1 is downregulated in numerous cancer, including bladder, gastric, breast, prostate, ovarian, and colorectal. Intriguingly, the evidence demonstrated that HAND2-AS1 was downregulated by promoter hypermethylation in various types of cancer [19, 20]. This key lncRNA acts as a sponge and competitive endogenous RNA with extensive targets, participating in proliferation, migration, invasion, apoptosis, and stemness [18]. Goa et al*.* studied HAND2-AS1 in cervical cancer and demonstrated that microRNA-21-5p targets HAND2-AS1. They postulated that HAND2-AS1 efficiently regulates miR-21-5p/TIMP3/VEGFA axis [33]. Another valuable study in bladder cancer conveyed that the oncogene microRNA-146 is sponged by HAND2-AS1 [34]. This study demonstrated the correlation between the expression of *HAND2* and HAND2-AS1, which was aligned with the previous studies. Also, evidence indicated that HAND2-AS1 expression might be under the control of DNA methylation, and further investigations are needed to prove this hypothesis.

Latterly, precision medicine considers each person's genetic and environmental factors in treating or preventing disease, particularly cancer management. One of the most focused approaches is circulating tumor DNA (ctDNA) released from cancer cells into the bloodstream, harboring tumor-specific genetic and epigenetic alterations. ctDNA analysis is beneficial for treatment and recurrence evaluation with minimum invasiveness [35].

Whereas ctDNA methylation could be more cancer-specific, *HAND2* DNA methylation may be a promising biomarker for detecting CRC in the early stage; furthermore, the probable recurrence of CRC.

## Conclusion

To conclude, we investigated and introduced public-available databases for the researcher with less computer science. We introduced the *HAND2* DNA methylation that occurs in the early stage of CRC, leading to the downregulation of *HAND2* and HAND2-AS1 expression. According to this In silico study and other In vitro and In vivo studies, downregulation of these critical genes leads to cancer formation in concert with other factors. This evidence has numerous consequences, such as perturbation of HAND2 downstream, increased stability of HAND2-AS1 targets, activation of ERK1/2 signaling pathways, and cancer formation. Further studies, particularly In vivo and fellow up studies, are recommended.

## Supplementary Information

**Additional file 1:** **Supplement 1.** Aberrantly Hypermethylation Genes Obtained From Gene Expression Omnibus Database.

**Additional file 2:** **Supplement 2.** Overlapped Hypermethylated Genes Between Results of GEO Analysis and Data Obtained From SMART Database.

**Additional file 3:** **Supplement 3.** Investigation of Hypermethylated and Downregulated Genes.

**Additional file 4:** **Supplement 4.** Methylation and Expression Data of CRC Cell Lines.

**Additional file 5:** **Supplement 5.** The interactive biological process of the HAND2 target genes.

**Additional file 6:** **Supplementary Figure 1.** Hypermethylated Regions of HAND2 CpG Islands and Their Correlation with HADN2 Expression in CRC Samples Obtained from SMART Database.

**Additional file 7**: **Supplementary Figure 2.** Hypermethylated Regions of HAND2-AS1 CpG Islands and Their Correlation with HADN2-AS1 Expression in CRC Samples Obtained from SMART Database.

**Additional file 8:** **Supplementary Figure 3.** Promoter methylation is correlated with gene expression in CRC cell lines.

## Data Availability

The datasets generated and/or analyzed during the current study are available in the Gene Expression Omnibus repository, https://www.ncbi.nlm.nih.gov/geo/ (including GSE17648, GSE25062, GSE29490, GSE47071, and GSE47592 datasets), ChIP-Atlas public repository, https://chip-atlas.org/, ShinyGO, http://bioinformatics.sdstate.edu/go/, STRING database, https://string-db.org, and DepMap database, https://depmap.org/. Also, The datasets analyzed during the current study are available from the corresponding author upon reasonable request.
