# Supplementary material for: Consequences of aberrated DNA methylation in Colon Adenocarcinoma: a bioinformatic-based multi-approach
Source: BMC Genom Data. 2022 Nov 29;23:83. doi: 10.1186/s12863-022-01100-7 (PMC9706923; doi:10.1186/s12863-022-01100-7)
Supplement: Supplementary file 1 — Additional file 1: Supplement 1. Aberrantly Hypermethylation Genes Obtained From Gene Expression Omnibus Database. [file 12863_2022_1100_MOESM1_ESM.docx]

**Supplement 1- Aberrantly Hypermethylation Genes Obtained From Gene Expression Omnibus Database.** We identified hypermethylation genes between CRC tumors and adjacent normal tissues from GEO datasets, including GSE17648, GSE25062, GSE29490, GSE47071, and GSE47592 by Venn diagram aberrantly. This study considered the list of common genes in five datasets for further investigation.

| **Names** | **total** | **elements** |
| --- | --- | --- |
| GSE17648 GSE25062 GSE29490 GSE47071 GSE47592 | 252 | ST8SIA5 HPSE2 PAX7 SKIP SLC6A2 DMRT1 CCNA1 PTPRR PRDM14 HS3ST3A1 S100P MSC POU4F2 FAM43B ST6GALNAC5 JAM3 LRFN5 SOX21 POU3F1 NOS1 WDR8 LOC349136 KCNA1 UCHL1 GNAS FBN2 ATP8B2 FOXE1 FBLIM1 RSNL2 SPRR2D ALK COL15A1 RASGRF1 TRIM31 SLC6A15 ATP4A KRTAP11-1 FZD2 LBP DEFB119 ZIM2 GCM2 LRRC4 SDC2 FCRL3 MAL SPG20 ZNF625 TRH ITGA8 CDH8 KCNQ5 DTX1 PNOC EFHA2 CRHR2 BPIL3 ADRA1A IL5RA OR1G1 CNR1 ATP10A RSPO2 NR2E1 THBD FCN2 RLN3R1 ZNF667 SFRP2 KCNB2 RIC3 CARD14 WT1 VGCNL1 STAC2 FOXL1 CDH18 COL23A1 ATP2B2 VEGFC CNGA2 CUTL2 PCDHGC4 PHOX2A HTR1E KCNK17 CHST10 SARM1 EDNRB DPYSL4 HAND2 CRISPLD1 SPAG6 KIF5A NPR3 CIDEA CNTNAP4 C20orf71 SOX17 IGF2AS NPY GAD2 ESR1 FLI1 TRPC4 PTPRT TRHDE ZNF677 LAMA1 ADCY8 EOMES SCARF2 FOXG1B NTRK3 TMEFF2 FBN1 BTG4 GABRA6 FIGN KHDRBS2 CCDC37 MDFI EFCAB3 C6orf32 GPM6A ACTN2 HNT HABP2 GPR26 HTR3D VIPR2 BNC1 AJAP1 ITGA4 IRF4 SCTR CD34 PDPN LECT1 ASGR2 AMPH WIT-1 CD1B GRM3 D4S234E CNNM1 SIX6 ZNF542 DEPDC2 C20orf185 SLC5A7 PCDH11Y GRASP CDO1 IRX4 GRIK1 POSTN INA DGKI ADHFE1 FLJ10781 C1orf158 UNC5C NCAM2 LCE3D BOLL NELL1 ZNF132 CACNA1A BPIL1 SORCS3 CNTN1 FAIM2 MSX1 LAD1 HBE1 PVALB ELOVL2 FCGR3B FLJ30834 JAM2 DLX5 CALCR ALX4 MMP26 FLJ25477 PCDH8 TRPC6 RYR2 SLC36A2 CBLN4 VSX1 GRIK3 GLRX SAA1 ZNF312 PCDH17 GPR109A KRTAP8-1 FLJ46831 COL4A1 LRRN6C ALDH1A3 DAK GAS7 SLITRK1 UMOD MEST HS3ST2 ACSL6 NRXN3 DOK5 OR1A2 BAPX1 C2orf32 EYA4 KRTAP15-1 GFRA1 GABBR2 PDE8B FLJ14054 TSCOT GRIA4 DCC TREM1 HKDC1 GPC6 BCAT1 CDKN2A BPI TM4SF19 TFPI2 ADAMTS5 SLC4A11 FLT4 C6orf155 TWIST1 TEX101 SFRP1 C20orf39 PIPOX GHSR LRRC3B GPR75 GRIA2 GATA4 PRSS1 CESK1 HECW1 CSF2 OR12D3 |
| GSE17648 GSE25062 GSE47071 GSE47592 | 94 | SLC4A1 KCNG1 HCN1 ODF3 PAX4 B3GAT1 CDH13 LBX1 GK2 ZNF454 FLJ25773 FAM5B SLC18A3 HTR2C SLC16A12 FAM19A4 SLC8A3 MAEL SLITRK4 EPHA5 OPRK1 FLJ36180 ADAMTS2 ARHGAP15 EGFL7 MYOD1 BFSP2 C10orf72 PDGFD SIRPB1 USH2A LPPR4 PLD5 DPYS ZNF134 TTC15 UNQ739 SPRR1A CHST2 TNFRSF8 SLC35F3 CST5 NUFIP1 CCDC8 PRKCB1 ADCY1 MAGEC2 KDR SLC27A6 MOXD1 FLJ23657 SMAD2 USP6 GDNF MYO3A SFT2D3 TREM2 SLITRK5 WNT2 KCNA3 SLC18A2 PROKR2 DEFB118 FGF5 SCN7A KRTAP13-3 FFAR2 CD1E EMR3 PPP1R16B KCTD8 PTPRM AK5 NDRG4 LOC124842 MGC35295 RELN C18orf34 MAMDC2 CLEC10A LDOC1 NRG1 ADAMTSL3 SYT9 AR SPATA8 SERPINB12 SNAP91 COL12A1 PTGIS SPRR1B NDN ZNF304 LCE1F |
| GSE17648 GSE29490 GSE47071 GSE47592 | 51 | AMICA1 MMP13 EVC SYN3 IQCF2 LCE1B FCN1 DDX49 ACSM2 MID1 DCD KAZALD1 C1orf165 MPO UCN2 GNS CFHR1 TBC1D3C IL21R FCGR3A CCND1 LRRC15 CSMD1 SMCR7 OR12D2 RGS18 TREML2 DMRTC1 PRAMEF2 OR51B4 SLC6A1 TNFSF11 TRIM49 TMPRSS3 STK31 TNS4 MGC4677 CHRFAM7A SOX14 SOX1 FCRL5 CFHR2 PRKAR1B IMPG1 LCN6 NALP14 TSGA13 FLJ40235 SEMA6B HIST1H2BO SOX2 |
| GSE25062 GSE29490 GSE47071 GSE47592 | 35 | MMP7 CPXM2 SIRPB2 FTHL17 RUNX3 HTR7 PXDN GALR1 ZNF80 PITX2 CD8A GRIN2A CBLN2 HMGA2 RXRG CX36 C1orf114 CMTM2 SPOCK NETO1 THBS4 DAB2IP FLJ90650 SCGB2A2 MCHR2 CNTNAP2 TTYH1 SLC13A5 KIAA0125 FLG ATP8A2 LCAT COL5A1 FBXO39 VAV3 |
| GSE17648 GSE25062 GSE29490 | 56 | ADAM7 TRPM8 TBX5 TRDN PODN IL17E UNQ467 ATP6V1G3 MYOT TM6SF1 PTGFR DEFB125 MGC39545 OR10J1 WBSCR17 MCAM MAGEA5 PCDH11X FLJ32447 FERD3L PI3 KCNIP1 CHODL DPP6 DPPA2 CD163 CCL11 KCNT2 SCGB1D1 SSBP3 CYP26C1 KCNS2 HOXA2 SLC30A8 RNASE3 POU4F3 CCL8 HSU79303 AKAP12 SOX8 DEFB126 ZP4 FLRT2 CASR TCERG1L OPRM1 TACR3 OR1N1 MGC33530 GPR87 EFCAB1 GSTM2 KRTAP13-1 NALP10 AKT3 UGT3A2 |
| GSE17648 GSE47071 GSE47592 | 68 | RLBP1 GAL3ST3 CEACAM6 MAGEC3 GPR143 MBL2 FFAR3 TRPM6 NXF2 NR0B1 ZNF549 GPR12 MYT1 SH2D2A GALR2 IL8RA PGLYRP2 FLJ33706 ZNF354C FRMPD4 DPCR1 C20orf91 SOX3 SLIT3 CLEC4M FLJ33860 ZIC3 S100A8 LILRA5 C1orf135 ADCYAP1R1 TBC1D3 LOC116123 CR1 KRTAP13-2 DEFB4 PPBPL2 TRPC5 GPR97 TAS2R9 CXorf6 PRG2 SLC7A9 CCL3L1 KRT20 KRT16 SNAPC5 CCL1 CRMP1 MAFB FLJ14816 IFNA1 MMP25 SEC14L4 FCGR1A GRAP LILRA3 LCE1D TNP1 NKX2-2 PEX5L C2orf21 EVPL ABLIM1 GALNTL5 RFPL1 CBFA2T3 FLJ46230 |
| GSE25062 GSE47071 GSE47592 | 82 | FLJ32110 BARHL2 ADRA2C TLX3 ADAMTS18 OR2B6 C1QC DNAI1 PKP1 UCN UTF1 TEKT1 SSTR2 CHST1 PANX3 MMP16 CASP8 CDH2 MGAT5B ZNF540 MAGEB6 SCUBE1 USP4 SLC22A8 WDR17 CELSR3 SH3GL3 VIM CCL20 FGF4 TRPM2 TRIM42 TSPYL5 MIA CNKSR2 DFNA5 GPR88 ELOVL4 DAAM2 PCDHB6 EBF FLJ32926 SORCS1 GSC NAP1L3 SYT6 ADAMTS1 T FLT3 MARK2 HLA-G FOXF1 GLDC ZNF415 GALNT14 ITGAX LOC112937 NEUROG1 MORF4 GLRB FGF3 SPSB4 MGAT3 ADRB3 MLNR ZNF135 LMX1B ST8SIA2 ACTA1 FOXF2 PAQR9 SLIT2 POU3F3 EMILIN3 RBP1 IVL HAVCR2 ADARB2 ADD2 PDE4A SLC6A3 ZNF471 |
| GSE29490 GSE47071 GSE47592 | 32 | MAGEA2 C22orf8 SIGLEC9 ELOVL3 PDZRN3 DLGAP4 TMEM44 CSTA TCF21 SFTPD MSR1 KIR3DL1 TMEM129 CES7 NUP50 SLC11A1 KIR2DL1 CTSG IZUMO1 CYP2C18 GNMT ASAH3 CXCL9 ELMO1 LACRT Gcom1 CLEC12A C21orf84 MGC39633 SERPINB3 LCE3C FLJ11155 |
| GSE17648 GSE25062 | 80 | PCDHB15 SOX7 SALL1 ADAM12 LILRA4 LPO POLG2 DKK2 GPR83 DRD5 NGFB NEUROG3 DMRT3 FGG ECRG4 SLC22A16 PSG3 OLFM1 CCR3 GLP1R SLC32A1 PRND OR6A2 PGLYRP1 NGB TYRP1 CHAT PDILT PATE FLJ39155 CPA4 NRXN1 PTF1A PMCHL2 EFEMP1 TAS2R16 CLEC4D CHRNA6 KLHL1 RARRES2 OTX2 CYGB KRTAP20-1 ZNF447 C20orf58 TRHR GRM5 TUSC3 AEBP1 GRID2 CHX10 NID2 APOC2 PTPRN LOC400120 CXorf36 MPDZ GSH1 OR10H3 MEG3 HIST1H3D TCTEX1D1 ADCY2 ZNF545 FLJ40919 ZNF671 IGFBP3 DNTT DLK1 PMCHL1 STMN2 MAPK4 DEFB129 CYFIP2 OLIG2 TMCO5 ALPL IFNA8 RPL39L C12orf54 |
| GSE17648 GSE29490 | 63 | MMP2 KLK6 NID1 LILRB2 LCE3E MAGEB3 GPNMB CD33 SEC63D1 DAB1 VN1R2 PIP3-E ERAF RNASE2 EBPL NURIT CMTM3 C3orf32 SLC6A18 OR7A5 FLJ36046 HK3 NR1I3 OTOA NPFFR2 FCRLM2 CATSPER1 DEFB103A LCE2D PI15 THY1 NPAS4 SST GSDML MAS1L IL18RAP RHO FPR1 FAM71C MGC3101 GDPD4 PRSS2 TCN1 MYEOV APOBEC1 SRD5A1 DEFA1 AZGP1 SPRR2A PRTN3 PPYR1 TYR ATP6V0D2 SERPINB10 GPX5 GABRA5 PCP4 OR5V1 LCE2C REM1 ADAMTSL1 OLFML1 NEK3 |
| GSE25062 GSE29490 | 47 | ANKRD45 NAALAD2 LAMA2 PDE1C C20orf186 BDNF LOC401498 CXorf20 KLHDC7B BTF3 MFAP5 TP73 FLJ30934 PHF21B FLJ45983 CHL1 GRM6 FLJ44674 CSMD3 C1orf188 KRTAP13-4 RAB11FIP4 GUCY2D HSPA1A ACCN5 FLJ27365 CART C7orf29 KIAA1944 ITPKB TAL1 AKR1B1 SYNE1 C1orf150 IRXL1 AQP1 ZNF660 GATA2 ADCY4 DLC1 SOX11 KCNK18 TSPAN4 KRTAP21-1 FCRL1 SSTR4 IFNA21 |
| GSE47071 GSE47592 | 190 | ABCA3 KCNK16 ERBB4 TSPAN32 BBOX1 VAMP8 IRS4 CHRDL1 NKX2-5 MTM1 ERG TPSG1 SLC16A14 HEPH ESX1 PTPRE SERPINA12 SLC12A8 GPR101 RBM35A OR1E1 DEFB1 KRT1 EPHA2 ARL11 CCL3L3 KCND1 GFPT2 GLRX2 ST8SIA4 HCRTR2 UNQ2541 CGB5 C9orf16 DNMT3B C20orf42 TMEM92 TFR2 FGD4 B3GALT7 RASIP1 FLJ22709 CHI3L1 CD53 CORO2B FLJ45256 NEBL HTR3C PICALM GSTP1 GNB4 MGST1 KRT17 MUC15 PRDM12 IL1RL2 CLCA1 C20orf151 PITPNA RTEL1 KCNK10 TNFRSF4 HOXB8 CHRNB1 COL6A2 NOXA1 IL23A JAKMIP1 LXN MAP7 ROPN1 ZNF264 CBS TRIM50C LRRC34 FKHL18 CD300LG SGK2 FLJ20245 PDCD6IP BTBD14A LST1 BMP4 ABCB5 HRASLS5 IL15RA AQP12A ASRGL1 FHL5 GRIA3 TMEM16G REG3G SPAG11 LOC283487 C3orf60 AIP HOXA7 CHD1L MTNR1A KIAA0746 FLJ11017 DARC AMPD3 C12orf22 EPHA1 TAAR6 UNQ9391 ARHGDIB FSHB GFPT1 EDIL3 ITGB1BP1 CRHR1 CD244 ZDHHC15 TCEAL2 SLC44A3 RFPL3 THEM5 CD80 HDHD3 LY75 IL1R2 ATP6V1E2 CTLA4 ADAMTS8 KCNQ2 VSIG9 HSPA12B RRM2 EDG4 MYL7 CPA3 F10 TNFRSF1B HYAL4 PLAC1 SLC16A3 CD300LB RALGPS2 HNF4A AMDHD1 LOC284837 GAS2L2 DRD4 WNK3 CRB3 KRT7 GPRASP1 RP11-49G10.8 SLITRK2 MS4A7 STK32B MGC9712 NOX1 MATN2 ABP1 DKFZp434I1020 MAGED4 KRTHA3B C1orf76 SLC6A12 CDCP1 CITED4 AIM2 PYGO1 FLJ43806 RSPO3 TRPC4AP C1QA AGBL2 HIST1H1D COL8A2 TMEM119 PDE1B RAET1E RPRM TGFBI ZFP42 TMOD3 VAV1 VHL GPR123 KBTBD7 PSD3 CDX1 ST6GALNAC1 GPR15 GHRH FLJ12700 |
| GSE17648 | 163 | PTGER3 DNAJB8 C9orf91 ZNF572 FLJ20444 CTNND2 C20orf195 KRTAP9-3 LOC348174 NOSIP UGT3A1 CDCP2 FLJ36116 CCBP2 ANXA3 C1orf132 SLC16A7 LAIR2 SYT10 ANGPT4 PABPC5 OSMR IQCH TP73L ZNF75A SLC6A6 TRIM29 MRGPRX4 SLC22A6 GNRHR COX7B2 CCL7 HBG2 NUPL1 C12orf59 PAEP DYM IL1F7 C8ORFK32 SLC34A1 TJP3 TEX13B SIM1 HPR GC ZIC1 C20orf160 GAGE4 KRTAP6-2 ABCC2 CCDC27 WFIKKN1 PRB2 CYP7B1 SLPI SLC26A4 SPINK7 CCL4 KLRG1 PAPPA2 LAT2 WDR78 PCDHAC2 ACE2 FAM107B TMEM27 IL1F9 ABHD7 TRIM51 C8orf34 CPO KLK9 KRTAP21-2 ARNTL ALS2CR11 MMP1 MS4A6E FBXO44 GLRA3 P2RY4 MX2 EDG6 C21orf100 FATE1 PSMAL EMCN ZNF610 IL19 COL9A1 C1QTNF4 CST4 INS AMY1A ACTL6B PLCG2 LCE1A C14orf39 C14orf49 NARG1L CRH LILRB1 GPSM1 GPR77 PSG2 KCNK9 C16orf28 DMBT1 MAGI2 CLEC7A C20orf79 HIST1H2AK FIP1L1 C6orf122 HIST1H2BI CACNG3 IFNW1 C1orf24 OTUD6A INT1 GLYAT P2RY10 GDF5 WFDC11 GUCY2F SLAMF7 SDC3 C19orf19 CNKSR1 PEG3 CCL5 EDARADD ZNF560 ENTPD1 PSORS1C2 TLR9 MSLN TNFAIP2 CFB TXNL6 MGC11257 FLJ35785 CCL15 OCM RRP22 PLAC4 DEFB123 SAA2 KRTAP22-1 TPSD1 CD300E FLJ21159 BCL10 CAPN7 HCRT CDA PZP ITIH4 HSBP1 PRODH2 ZNF645 KCTD1 LBR AQP7 |
| GSE25062 | 126 | RBP5 GAPDHS STYK1 GUCY1A3 C8A UGT2B17 SH2D1A FKBP7 PCDH21 PHACTR3 CPNE5 SCUBE3 CEACAM8 CD38 APEH ZNF96 NPAS1 ETNK2 RAB37 TPM3 KRTAP19-1 FLJ40365 FOXD4L1 CHFR SSX7 UNQ9433 FOXB1 CD40 GBGT1 FGF8 FOLR1 XTP7 ACTRT1 FADS2 TCF4 C8orf37 LONRF2 HOXA3 BNIP3 NDRG2 CRB2 CASP14 GPR45 RUNX1T1 CYP2E1 LRRC61 C20orf100 HNF4G FLJ37478 RAXL1 SEMA7A AGTR1 WIF1 UBD LMX1A GPR124 SULT4A1 TSSK1 CTNNA3 SCGB1D2 KPNA1 SPATA16 MRGPRX1 ANGPT2 C2orf10 SHD FOXD2 PAK7 HTR1B CDH22 SOX5 VPREB1 GZMA LPA ODZ1 ZNF285 DZIP1 HCN2 HOXC9 PLCL1 HPCAL1 ZNF300 FBXL22 COL14A1 BRSK2 FLJ90166 SNRPN GATA5 NKX6-2 LY6H LOC122258 FLJ31659 RILP PTPRO C9orf79 IL7R GRM7 MOS PCDHB1 DYDC1 SULF1 HCN4 SPRR3 KCNB1 PIK3R1 WFDC12 SEC31L2 ASB5 CDH12 ACOT4 BRS3 EDG3 ZNF662 PTHR2 PEG10 BAALC LOC348840 CIDEB RAFTLIN LCE4A SLC5A8 AP1S1 CYP1B1 EFS IL11RA GNRH2 |
| GSE29490 | 250 | GPHN NMBR ABCC6 IER2 NAGPA CSPG4 CFHR5 LDB3 WNK2 KCNA6 C18orf20 FOXQ1 DOCK2 RNF41 CSEN CYLC2 CXCL13 NR2F1 TRIM22 FNDC6 BASP1 NR1H3 DACH1 LGI2 PWCR1 GML A2ML1 HS3ST1 CNTN4 ASTN DNAJC5 IGSF4C ZNF154 SLCO2A1 FOXA2 TMEM98 C9orf150 SYT12 MYH6 RASD2 PARVA SLC35D2 A4GALT GALNT5 NIPSNAP1 CDC42EP3 PENK TSC22D3 C21orf94 CDX2 CST3 AKNA DSC2 LPXN AKR1C2 SPRY2 LTBR MYH1 RASEF GPRC5C BTBD4 EMR1 KIAA1018 LOC387758 TFEC TMEM25 PROX1 TBX21 SOX9 C20orf114 KCNJ9 LRIG3 RAB27B SNAP25 EDG2 C3orf22 DGKZ CMA1 NBLA04196 EDN3 ASTN2 C21orf63 FCER1A KCNMB1 AFAP FGFR2 ABCC3 ATG5 EGLN1 LGR5 MARVELD3 MYCT1 LOC119710 KRTAP26-1 C12orf46 SH3BGRL2 MAP6D1 PTGS2 GCET2 C20orf133 OBFC2B FCAR UACA IL18 TAS2R1 MGP GABRG3 SGCZ FLJ46365 SCNN1B PTPN14 FLJ13265 GOLGA5 MAGEA3 OVOL2 ITK FLJ10661 LYZL6 C10orf10 PSG1 FZD10 C16orf52 MALL LCE1E ARRDC4 TXLNA RAB31 PRP2 C21orf25 GYG2 C3orf37 AADAT FILIP1 IGFBP5 HAK SIGLEC6 OR10A4 STATH BMP10 NR2F2 PSTPIP2 FGD2 TEAD4 FMNL1 SH2D4A C19orf4 CDH3 MCF2L NPAS2 IQGAP3 PARVG FAM20B LMO7 BTBD3 PPP1R13L GRB10 HOXD8 TMEM45B PTPRS TMEM51 MFSD7 DNMT3L B3GNT3 PTPRD CYP4X1 ARX HTR2B BACE1 TNFSF4 RBP4 ADCYAP1 CYP2S1 SEPP1 WDFY3 KLF8 TNK1 OCLN GNAZ LILRB3 NFIB PLAT GNAI1 TCEB3C PPFIA2 TCL1A FOXA1 ERBB2 HOXC4 CDH1 OSR1 CALML3 TUBB6 FXYD7 MGC16291 SCT LOC148137 DLX3 ZNF541 SIGLEC12 SLC16A4 BTBD11 SPTA1 CXCL1 STEAP2 C19orf33 TGFA PRLR MGC52057 ZFP2 KCNA5 PDE4B SSTR1 APOBEC3C CTDSPL LILRB5 CD209 FAM84A ENO1 OR1F1 SP3 EXDL2 GPR24 FBXL16 ELA3A IFNA14 CAP2 GLI2 MPP3 TCL1B LAMB2 DNAH3 LAMC2 MAGEA11 HOXB9 KIAA0367 SIM2 TTC23 GRP SV2A RTKN MLZE FAT STAC KCNC2 KCNH7 SSH3 KLK1 CD164L2 AYTL1 MRPL16 OVOL1 ASF1A MARCO SEMA5A SCRT1 CST2 PRDM15 KITLG ALAS2 SEC61A2 NXF5 FLJ45964 KCNK5 TTPA ERRFI1 NPBWR2 SALL3 LRAT ANXA4 ADAM15 SPINT2 GPR39 ZNF214 CYR61 ITGB5 GULP1 SYNGR1 SEMA3C FAM13C1 TRMU KLRB1 ASB2 PRR3 HBG1 KCTD14 |
