# Supplementary material for: Consequences of aberrated DNA methylation in Colon Adenocarcinoma: a bioinformatic-based multi-approach
Source: BMC Genom Data. 2022 Nov 29;23:83. doi: 10.1186/s12863-022-01100-7 (PMC9706923; doi:10.1186/s12863-022-01100-7)
Supplement: Supplementary file 2 — Additional file 2: Supplement 2. Overlapped Hypermethylated Genes Between Results of GEO Analysis and Data Obtained From SMART Database. [file 12863_2022_1100_MOESM2_ESM.docx]

**Supplement 2- Overlapped Hypermethylated Genes Between Results of GEO Analysis and Data Obtained From SMART Database.** Identifying hypermethylated genes from the GEO and SMART databases. By construction of the Venn diagram, overlapping genes were identified.

| **Names** | **total** | **elements** |
| --- | --- | --- |
| SMART Hypermethylated Genes  GSE Hypermthylated Genes | 202 | PAX7 PTPRR HS3ST3A1 POU3F1 FOXE1 SDC2 SPG20 CDH8 KCNQ5 ADRA1A ATP10A ZNF667 SFRP2 ATP2B2 DPYSL4 SPAG6 NPY FLI1 TRHDE ZNF677 LAMA1 GPM6A SCTR AMPH CD1B GRM3 SLC5A7 IRX4 ADHFE1 C1orf158 NCAM2 CNTN1 HBE1 PVALB CALCR GRIK3 DAK HS3ST2 DOK5 TREM1 BPI FLT4 TWIST1 GPR75 HECW1 OR12D3 ST8SIA5 SLC6A2 DMRT1 CCNA1 PRDM14 POU4F2 FAM43B ST6GALNAC5 JAM3 NOS1 FBLIM1 ATP4A FZD2 ZIM2 LRRC4 FCRL3 MAL ZNF625 ITGA8 CRHR2 CNR1 NR2E1 THBD CDH18 VEGFC PCDHGC4 KCNK17 EDNRB CRISPLD1 CIDEA SOX17 ADCY8 BTG4 GABRA6 KHDRBS2 IRF4 PDPN PCDH11Y CDO1 GRIK1 INA DGKI LCE3D BOLL JAM2 ALX4 CBLN4 VSX1 GAS7 ACSL6 GPC6 CDKN2A TM4SF19 TFPI2 ADAMTS5 SFRP1 GATA4 PRSS1 HPSE2 LRFN5 KCNA1 GNAS RASGRF1 SLC6A15 DEFB119 IL5RA RSPO2 FOXL1 COL23A1 HTR1E CHST10 HAND2 GAD2 ESR1 TRPC4 EOMES SCARF2 TMEFF2 FIGN CCDC37 MDFI BNC1 AJAP1 LECT1 D4S234E SIX6 POSTN UNC5C ZNF132 SORCS3 ELOVL2 MMP26 TRPC6 GLRX PCDH17 COL4A1 ALDH1A3 UMOD NRXN3 GFRA1 GABBR2 GRIA4 PIPOX S100P MSC SOX21 UCHL1 FBN2 ATP8B2 SPRR2D ALK COL15A1 TRIM31 KRTAP11-1 GCM2 TRH DTX1 FCN2 KCNB2 WT1 STAC2 PHOX2A SARM1 KIF5A NPR3 CNTNAP4 PTPRT NTRK3 FBN1 GPR26 VIPR2 ITGA4 CD34 CNNM1 GRASP NELL1 CACNA1A FAIM2 MSX1 DLX5 PCDH8 RYR2 SLC36A2 KRTAP8-1 SLITRK1 MEST EYA4 PDE8B DCC HKDC1 BCAT1 SLC4A11 GHSR LRRC3B GRIA2 CSF2 |
| SMART Hypermethylated Genes | 5950 | MSRB1 PNCK AACS FSTL1 CREB3L1 ABCA3 MMP2 FEZF1 SAMD4A PP12613 KCNMA1 C10orf90 SMARCD3 PKNOX2 LINC01201 RALYL OSMR-AS1 GPR98 BTG2 ITGA5 RP11-1057B8.2 RXFP3 RP4-798P15.3 GLT1D1 CYP11A1 RP13-104F24.2 CLK4 KCNG1 FOXC2-AS1 SALL1 CADM4 SLC25A3 OTOP2 ACOT12 AC058791.1 CTD-2233C11.3 BSN-AS2 NTRK1 DDB1 SLC6A11 NAP1L2 CRNKL1 NTSR1 GPSM2 RTN1 CPXM2 RP11-234B24.4 MIR515-2 OR2W1 RNASEH1P2 LRRC71 KCNA6 C4orf22 TRGC2 ZEB1 CDK14 ERG ZSWIM1 FAT4 DIRAS1 CHD9 SALRNA1 RP11-35N6.1 KCNK3 RP11-278J6.4 FGR MSRB3 AC079135.1 DEFB133 PDE1C RP11-523O18.7 ADAM7 NINL TVP23BP1 MIR489 WSCD1 GRM2 RP11-362K2.2 CAPS RP11-574O7.1 KNDC1 LRRN1 FLJ26850 RP11-156P1.2 EVC TMEM132E NFIA SYN3 TP53AIP1 SUGCT LHFPL2 EPT1 RP11-74E22.4 TRGJP1 CCL22 RP11-74J13.8 OR10X1 SLC12A4 RP11-461O7.1 AC009264.1 SLC26A10 BMP3 CTD-3006G17.2 TMEM19 RUNX3 LUZP1 PRKCB EIF4G3 RP11-79P5.3 RP4-794I6.4 A2M RP11-164C12.2 LINC00460 LRGUK AC104389.28 FEV CPNE4 RNF32 GRK5 CTC-353G13.1 SLC8A1 CLDN16 RP11-81M19.1 CLC ISL1 VGLL3 SPAG11B ZNF83 C1orf61 PDCD4 RP11-196G11.2 SRRM4 AC093887.1 ARHGEF26-AS1 CST7 SELL OR8J3 ANO5 C16orf45 LTBP4 GFPT2 PYY KCNC1 OR8J1 ARHGEF9 OR9G1 ABR RP11-415I12.3 LINC01121 SIAH3 GML LINC01574 CNGA3 CD38 NAT8L KLK8 NEFH ALX1 ZNF257 DUSP4 PODN RP11-521O16.2 LINC01212 RP11-22P4.1 RP11-666E17.1 MAOA ZNF385B FBXL14 MIR518A2 RP11-446H18.5 RP11-671J11.5 VAT1L TNNT3 BPIFB2 GYPC C7orf33 C12orf40 LINC01163 LGMNP1 ST8SIA4 AC079613.1 RP11-706C16.8 KIAA1211L RP11-257K9.8 TPM3 FAM19A4 CHST15 CTA-384D8.31 B3GNT7 GDF10 A2ML1-AS1 SLC6A7 TRAPPC9 RPS11P6 MIR1225 P2RX3 PANX3 TUBB RARRES3 CTD-2231H16.1 FNDC1 TMEM255A CD82 RP11-398K22.12 TMEM26-AS1 AC133644.2 VAX2 SAA2-SAA4 LINC00605 RP5-896L10.1 RUVBL1-AS1 SLC24A5 BCL2 HCRTR2 MMP16 CAMTA1 FAM159A MIR654 RP11-358H18.3 SAMSN1 SPANXN4 FAM198A PLCL2 SEC14L1 NME8 CHFR GKN1 COL18A1 PXDN RP11-151A6.4 CTB-167G5.6 FCN1 AC067959.1 TNXB C1QL1 VIM-AS1 EMX2 FTMT TOX3 CRHBP CHST11 NPR1 DNAH11 ASS1 SLC9A9 EN1 GSTM4 AC009501.4 F7 RCSD1 RP11-855C21.1 RP11-576E20.1 ADAM23 SPTBN1 EXOC2 SPX CDC42EP3 KRTAP24-1 PTPN5 IDO2 HLA-J GNA11 TACR1 RP4-800J21.3 SMAD5-AS1_2 GRIK2 STMND1 IGSF11 RASIP1 DPYD RP13-577H12.2 BTF3 SLITRK4 TDRG1 CACNB1 RP11-563P16.1 QKI NCAM1 SLX1A OTX2-AS1 FAM24A PDP1 RP11-588P8.1 KRT71 MIR935 RP11-420J11.1 RP11-432M8.18 CNTN2 RP11-148L24.1 TKTL1 MIR885 CHRNB2 CDC14C COL7A1 NFIL3 TM6SF1 FUT4 DBH SEZ6 NANOS3 TFAP2B ECEL1 RP1-23E21.2 CIITA CLDN6 EPHA4 CMTM3 ZNF655 AC005013.5 MRPL15 CTD-2015G9.2 PLCXD2 NEU1 PP14571 TARID CLEC4GP1 OSR2 ARHGAP15 CYP51A1P1 GNB4 CDHR5 SPATA4 NBPF3 HBG2 FAM218A PRR5 CTD-2299N12.1 CCDC172 C19orf67 LONRF2 PALM2-AKAP2 CDC20P1 MIR206 LINC00486 RP11-554D14.6 DEFB125 AHNAK SCN11A GFI1 ARHGEF7-AS2 RP11-230C9.1 FHL1 RTN4RL1 RP13-507P19.2 TTTY14 HK3 RP4-678D15.1 LRRC53 ZFHX4-AS1 E4F1 C16orf62 IRX3 ELFN1 RP11-122L9.1 KCNA4 RP11-615I2.2 NMNAT2 SORBS2 PSMF1 SH3PXD2A MUC15 MAGEB6 IFITM10 RP1-111C20.4 PRDM12 RP11-672L10.3 SCHIP1 SKAP2 RP11-631N16.4 PRND 11-Mar HS1BP3 AC009487.4 HUS1 C1orf112 OR2B2 KIF19 HAPLN4 GALR2 TFEC RP11-360L9.4 PPM1H GPBP1L1 AC010886.2 TBX21 RP11-492L8.1 MIR503HG TNFAIP6 STRIP1 RTEL1 LINC00682 KCNK10 FSTL5 OR6A2 RNF182 ZNF738 AHRR RP11-927P21.4 UPP2 VMP1 ARFRP1 LINC00517 ZNF487 SLC46A2 HRH2 PCDHGB4 AC005251.3 PITX1 CTD-2297D10.1 PRR36 IL17D OR56B4 GPR158 LDLRAD2 RDX KCNIP4-IT1 VRK2 CELSR3 ZNF568 SVIL EHBP1L1 DCTN1 CAMK2B APBA2 GRIP1 RP11-700H13.1 TCF21 RP11-82L7.4 STOML3 RP11-540B6.2 WNT6 FTH1P8 RP11-756A22.7 NECAB2 AC008060.5 RGS20 CMA1 MTRNR2L8 RP11-397H6.1 TRAM2 AF146191.4 TGFB2-AS1 VWCE ARHGEF26 LXN AC007682.1 CTD-2203A3.1 NBPF13P ABCC12 RELL1 RP11-175K6.1 PREX2 RPS20P35 ASTN2 LINC00577 PI15 ELAVL2 RP11-210M15.1 RPN1 KLF2 FAM160B1 TAB2 RP11-706C16.5 SIX2 TNFAIP8L3 NXF4 NKX2-2-AS1 ADAMTS19-AS1 SNORD116-7 AC073071.1 ZNF726 CSMD3 DLGAP1 RP11-1134I14.8 UGT1A8 NTN4 ASCL2 PCDHGA10 SH2B1 SHH TYRP1 CTD-2089N3.2 RP11-288A5.2 EDDM3B GPR135 LGR5 NKX2-8 RP11-49I11.4 SIRPG ZNF264 CTD-2207P18.2 METTL5 AKAP2 CD8A PRRT1 LBX1-AS1 PLEKHM3 LRP1B ENKUR PLA2G4C AC002454.1 MIR6080 RP11-944L7.4 CASC10 CA9 ZNF710 KRTAP26-1 RP11-586D19.2 TLX1 OR2M2 RP11-486O13.2 VAX1 SECTM1 OR8K3 TRAV3 NAA60 RAB11FIP4 USH2A SUMF1 C5orf56 LPPR4 OLR1 SQLE DAGLB CTD-2533K21.3 NEGR1 FAM49A EHD2 C10orf53 CTB-43E15.1 SPG7 SCRT2 HOXA5 XXYLT1 FGF12 INPP5B RP11-624C23.1 IL20RA CACNG2 PCDHGA5 RP11-248B24.1 TLR5 RP11-307P5.1 MIR4439 BTBD9 ZEB2_AS1_4 AC022153.1 FPGT-TNNI3K GALNT9 ICE1 SAFB2 BEGAIN HHIPL1 RP11-480G3.1 CTB-57H20.1 RP11-575F12.3 SLC9A2 KLRG1 ADD1 ZSCAN12 RP11-66B24.8 TUSC7 RP11-272K23.3 CLDN10 RP11-889D3.2 SNORD115-14 CCDC144A TAC1 SOST HPCAL4 NFAT5 MIR762 SRGAP2C RP11-88I21.2 WIF1 CMKLR1 OR11H4 OR52A1 SCNN1B SLC44A5 PPDPF RP11-489D6.2 NOL4L CPA4 MEGF11 RP11-681L8.1 SDK2 BAIAP3 PTP4A3 KCNK7 PODXL RP11-281P23.2 CCT6B SNCA ZNF542P ERICH3-AS1 LINC00882 ESRRB ARHGAP26 PRCD RP11-945C19.4 GS1-72M22.1 RP11-896J10.3 ZNF536 ITGB2 RP11-21A7A.2 TRDJ2 SPATA32 HS3ST5 CD72 EPB41L3 TEKT2 WSB1 MAP3K6 MAP6 CTD-2503O16.4 DDIT4L OR2L13 PARVB RP5-1110E20.1 ELL RTP1 SETBP1 RP11-767I20.1 CDH11 MIR548A1 RNF175 NRSN1 AREG ZBTB7C RP1-60O19.1 MME RP11-491F9.3 VAT1 SHANK3 GRIN2A MAST4 CEP72 RP11-393N4.2 RP11-565A3.1 MYC NRXN1 RP11-527D7.1 MFI2 NR4A3 SPDYA DNAAF5 DSCAM-IT1 MBL1P RP11-467K18.2 CPO SOCS3 RP11-520P18.5 ULBP1 MIR2114 AGBL1 AC144450.1 PARK2 AC069368.3 GPR124 NELL2 VWA3A POU3F2 HLA-V RP11-482M8.3 PIANP OR52N1 DPY19L2P2 RP11-281O15.4 IL1RAPL1 VAV3-AS1 WDPCP NKX2-1 GAL APOBEC3F COCH RP11-483P21.2 KLK9 WT1-AS_7 PCYT1B RP11-445F12.2 GNGT1 PPATP1 VPS37B RP11-49G10.3 LINC01111 PCDHGA4 ADAMTS17 CBLN2 KIF5C RP11-348F1.3 MATN4 PPP1R17 RP11-382E9.1 OR5W2 RP11-293M10.6 SPATA16 TRIP10 RP11-353N14.1 XXbac-BPG154L12.5 PIAS1 NPHP3 ADCYAP1R1 MMP1 AC140912.1 FSD1 CDH20 ST6GALNAC3 SPINK13 SNORA71 MAGI2-AS3 RP11-669N7.2 OR8B4 LRRFIP1 TFAP2A-AS1 SNORD113 MROH5 LINC01483 RP4-800O15.2 SATB2-AS1 IL18RAP RP11-382A20.5 TNR-IT1 RP4-738P15.1 SLIT1 KRT86 A1BG-AS1 FZD10-AS1 DPP6 AF131217.1 AC003005.4 ACOX3 RP11-227D13.1 ADCY1 ZNF846 GRID1 UBE2QL1 CR1 RPS6KA2 LINC00161 KRTAP13-2 ADAMTS12 DAB1-AS1 RNF217-AS1 ZNF287 DAAM2 DPPA2 FOXG1 MAGEA10 RAD21L1 TBC1D16 PLXNA4 AMTN GRIN3A FPR1 FMNL1 CHTOP RP3-412A9.16 SYN2 LINC00504 SMOC2 CTD-2311M21.3 ARHGDIB KCNH1 HFM1 ANXA2 WTAPP1 MCF2L ACSS3 SPEG CTD-3179P9.1 CNBD1 MIR517B FAM87A RP4-576H24.2 DCDC1 RP4-597A16.2 LINC00505 MAGEA8 LINC00463 PARVG MUC22 CACNA1C TRIM77 EGR4 MAP3K14-AS1 PDCD6 RP11-348F1.2 BHLHA9 5-Sep ZNF775 CCL11 RP11-342M3.2 LINC00900 IDO1 OBSL1 RP11-544M22.13 RP11-240B13.2 STARD8 GRAMD1B FOXP2 ANKRD18B TGIF2LY HTR1A NLGN4X GDPD4 FBXO34 LARP1BP1 RP11-227F19.1 GSC LINC01109 LALBA UNCX RP11-90C4.1 XX-CR54.1 BPIFB1 SLC29A4 RP11-509A17.3 CRHR1 RP5-983L19.2 RP4-555L14.4 LPAR3 LINC01388 NRN1 FAM19A5 SHE ANO4 LINC01587 RP11-313J2.1 KCNQ4 PPP5D1 ANKMY1 C7orf50 CXCR1 UNC119B CR1L RP11-462G12.1 TCEAL2 TUNAR FAM153B FAM184B HMHA1 LINC01393 RP11-616M22.11 ADAM29 CTNNA1 LINC00595 C15orf26 ELFN2 CT47B1 CTB-158D10.3 FOXN3 CCDC3 ADCYAP1 KIAA0513 CTD-2319I12.5 PNMA2 AGRN YY1P2 ANKRD34C-AS1 RP11-8H2.1 SPANXN5 SPANXN1 RP11-613D13.5 PAK7 KCNS2 RP11-578F21.6 SLC30A8 LIFR-AS1 HIC1 IAPP RP3-507I15.2 LRP1 NKX2-3 ZNF790-AS1 AF013593.1 AC004076.9 CTD-2134P3.1 RP11-466L17.1 CTC-523E23.14 SLC6A1 C20orf85 SERTAD4 DDI1 PAX6 RPSAP52 SCG2 RP11-470C13.4 TREM2 FMO3 SLC17A6 KCNK9 MIR544A ITGBL1 AP000438.4 NETO1 DEFB134 THSD7A NDST3 OVCH1-AS1 LHX4 GALR3 NBEA GNAI1 GCNT6 EGFR IZUMO1 ZSCAN5B SFTA3 TESPA1 FAM19A2 NDRG1 CPXM1 PTPRQ APPL2 OR5L2 ZNF285 RASGEF1B ACOT11 AFAP1 RP11-9G1.3 PKNOX2-AS1 OC90 MSRB2 DPY19L2P1 RP11-183E9.2 DZIP1 TIAM2 RP11-261N11.8 RP11-139I14.2 OR51B2 FAM150A CFAP54 CTD-2514K5.4 SNX32 TRIM17 RORA-AS1 RP11-3B12.2 RP11-52L5.6 SLX1B-SULT1A4 TMEM196 ADAMTS8 HOXC4 RYKP1 MIR3185 RP11-444A22.1 MIR762HG C14orf37 UGT1A7 MYT1L TMPRSS3 AC019205.1 UPB1 RP11-631N16.2 KCNA3 IQCJ-SCHIP1 PIF1 RP11-17E3.1 LINC01102 TRHR SOHLH2 SLITRK3 LRP8 RNF152 CHRDL2 RP11-373N24.2 MIR99AHG AC006262.10 RP11-114H23.1 AKR1D1 CLPTM1L ITPKB MEOX2 STK31 CTD-2015B23.2 ZBED9 SH3D21 KIAA1257 SCT SKI RP11-342M3.5 PLEKHB2 ZNF544 USP2-AS1 CAPN11 AP003025.2 ARHGEF4 RP11-184A2.3 KRT16 UBE2O KLF17 RP11-46D6.4 RP11-286H14.4 CDR1 MIR638 FBLN2 LINC00485 RP11-95F22.1 ROBO4 RP11-402C9.1 RP11-466P24.5 HBBP1 NRG3 APOOP1 F10 GSC2 GIF COMP RP11-69M1.4 RTEL1-TNFRSF6B THBS1 TMEM52B TAL1 ARMCX1 ABL1 PTPRG MOCOS TXNRD1 BRDT RP4-655C5.4 RP11-834C11.12 BTBD11 DCAF4L2 ELMO1 RP11-305P22.9 GPER1 FAM184A UNC13A SLC24A4 BARHL1 PRX OR13A1 RP11-340E6.1 SYNE1 SNORD113-6 LINC00243 HRH1 IL25 HIST1H3C RP11-420A23.1 RP11-936I5.1 SPANXA1 RASGRP3 DRD4 SEMG2 ARL5C SOX14 TMEM200C GRPR KIAA0922 NTSR2 ZBBX PKHD1L1 ZBTB10 LINC01018 CTD-2330J20.2 HTRA1 LRMP PDZD2 MCC P2RX2 WISP1 RP11-148E17.1 OTOP3 ATP2B4 CHST12 CTD-2527I21.4 NME5 ZNRD1-AS1 BARX1 PLXNB2 RP3-481A17.1 DNAH10 GAS2 HBM CACNB2 GRXCR1 PDE10A EN2 STPG2 TOX2 PDHX HBB RP11-10J21.5 RN7SL815P BPIFA3 GNG11 AC079779.4 CASR MT1HL1 LINC00640 CANT1 METTL24 RHOJ IRF2 SLAMF7 GNG7 CDC37 VSTM2A PGR RUNX2 FCRL5 SOX1 RP4-684O24.5 ODC1 ZBTB8B NR2F6 ZNF730 AC073464.4 KRBOX1 SYBU TSPEAR FILIP1L L3HYPDH COL4A6 GAGE10 RP11-626H12.2 SCN7A ASIC4 GAS1 ZNF793-AS1 CCDC168 PEG3 TSPAN18 SLC35G2 TRPM3 MCF2 RP11-944L7.5 STK32B CAPSL ZNF532 FECH TYR AOC4P TACR3 SLC7A7 RP11-664D7.4 HAND2-AS1 IL10RA KIF1A SLFN11 VCAN TMEM64 CYP27C1 MED12L LEKR1 DNAH8 TSLP ADAP2 CNTNAP5 ZEB2_AS1_1 SLC1A3 CTD-2528L19.4 HAO2 CTD-2194D22.5 RPS27P25 PCDHGA11 RNH1 RP11-497E19.1 CDH7 RP11-81H14.2 RP13-150K15.1 MIR2682 RP5-858L17.1 OSBP2 PRRG3 LRRC18 RP11-699A5.2 AC105342.1 SULF1 FGF13 CTD-2525I3.6 PRDM5 RP11-534L20.5 UBE2E2 CTD-2355J17.2 EHD1 PRKG1-AS1 ILDR2 EFCC1 C2orf15 KCTD8 NEUROD6 ZNF550 CDV3P1 TRAV12-3 LINC00607 HCN4 OR8H2 CPB2-AS1 HFE2 UNC5D TRIML2 AP000282.2 RP11-637O19.2 LINC01317 RP4-663N10.1 FAM168A ETV3L RP6-191P20.4 ZNF830 QRFPR FAT1 ISLR2 B3GNTL1 ZNF660 MGAT3 KRTAP4-7 NREP CH17-140K24.2 RP11-510M2.1 SIM2 GJC1 RP11-575F12.2 GATA2 RPP25 TANGO6 C20orf194 SBSPON GABRA5 CTC-550B14.7 RP11-42A4.1 KIRREL GPC3 PIK3R1 AC013460.1 MAGEA12 PDGFRA ADCY4 CTB-31N19.3 TET1 ADRB3 DMD PRRT3-AS1 RP11-1070A24.2 ADCY2 ARHGAP36 OR6C65 FAM178B CRYL1 KCNQ3 ANKRD33B NOVA1 AC025470.1 KCNC2 CTD-2337A12.1 MVB12A SSH3 CCBE1 ZNF135 RP11-656G20.1 AC145123.2 RP11-545A16.3 COL26A1 RND2 LINC01006 NRSN2-AS1 CTD-2135J3.4 MYH4 MASP1 JAKMIP3 WFDC10B RP11-42O4.2 ADAMTS9-AS2 ASTN1 AIFM3 MROH6 PCDHGB3 PCDHGC3 ASB5 CAV2 ATRNL1 FOSB TMEM232 SUN3 SLC27A2 AC092667.2 ARHGAP35 RP11-227D2.3 ARMC4P1 FAM46C NEK9 CHRM3 KDM2B OVOL1 PREX1 DCHS1 AKR7L TPPP C8orf49 UST TIGD3 MIR4500HG PDCD1LG2 CALD1 LCAT ERICH1 ARHGEF25 POMC AC087499.10 ZNF404 KRTAP19-8 ADORA2BP1 RPL31 RP11-230G5.2 MKX KIAA1462 ZNF814 KDELR2 LY9 ATP2B3 RP11-705O24.4 PYGO1 TLE4 ZNF570 OR2M3 PKP3 MRPL23 RP4-613B23.5 COL24A1 NFKBIA ANO3 HIBADH CTB-118N6.2 IGFBP3 AL132709.6 GAK HOXA6 CRTC1 TTC9B CTD-2353F22.1 CPT1A LMO1 CNTN4-AS1 DCN TMEM132D GSE1 ACTBL2 WT1-AS_8 CTC-537E7.3 AC083900.1 COL11A1 RP11-716D16.1 FOXF2 S100A12 SNORD114-22 CTD-2561J22.5 KRTAP22-1 HLA-C SLC13A3 SPRED2 NXF5 TIMP4 PITPNC1 SLIT2 AC012594.1 CX3CR1 POU3F3 PDE1B MIR921 ITGAE LRRC37A17P SLC30A3 RP11-539L10.2 RP3-510D11.1 NKX2-2 ZNF804A AC011288.2 GATA2-AS1 ANKRD7 GDF7 RP11-654A16.1 PTCHD1 PPIAP17 PNMA3 CH17-270A2.2 CIDEB RP11-99E15.2 FAM20C ABCC8 RP11-219A15.1 OR2T6 BNC2 AC015691.13 KRBA1 MAGED1 XKR4 FBXO39 STMN2 LPPR5 STAB2 EIF3IP1 CDC20B LINC01143 TNPO2 MCPH1 ASS1P1 C9orf3 ROR1 LBH SYNM PPM1N RP11-219E7.3 BHLHE23 KRTAP13-1 WNT7B CCDC178 SLC4A4 AL132709.9 AP000662.4 ADAMTSL1 KAZN VENTXP1 ZBTB46 RFC5 MAP7D1 OR52L1 RP11-19O2.2 RP11-779P15.2 EPO PAX5 AOX1 ZNF682 COL1A2 DTNA AKT3 ADCK5 CRB1 PLK5 UBR4 RP11-770E5.1 MAB21L1 PROCR RP11-44K6.3 LINC00472 SYT4 GULP1 XPO7 NR1D1 RP11-71L14.4 TRIM5 KCNS3 PARD6B RP11-140A10.3 SRPK2P LINC00883 PCDHGA3 AC134300.1 RP11-32K4.1 THEMIS VWA3B RP11-141E13.1 LINC00404 ZBTB40 CH25H AC096553.5 COL12A1 ALPL SMAD6 STEAP2-AS1 MEDAG CHST3 GPR123 RN7SL418P LINC01210 ABLIM1 RP11-702C7.1 ADAMTS7 MAST3 TFAP2E LLNLF-187D8.1 COLQ RBMS1 PDCD6IPP2 NRXN2 SNORD108 MAEA NYAP2 TDGF1P3 RP6-24A23.3 PSD3 ABCA13 NLRC3 RP11-436D23.1 SRSF12 MAPK8IP1 RFX2 AC108142.1 LINC01548 ZNF519 HOPX MEF2C FAM216B ZNF304 CTD-2571L23.8 IGFL1 CBFA2T3 CDIP1 FOXO1B AFF3 COG7 SNORA60 LCE1F ESM1 SLC44A4 EFTUD1P1 PTGER3 USP29 RP11-76I14.1 RPTOR RP11-556E13.1 RP11-143K11.5 ERBB4 AMER2 AC078882.1 CTC-523E23.6 KIF26B ZAP70 PTN C1orf106 CTB-120L21.1 LPP XXbac-BPG32J3.19 MIR4785 CHST8 ANKRD34B SLC22A18AS RP11-154D6.1 HSPB6 SOX7 IRS4 CTD-2369P2.8 RP11-349F21.4 MACF1 CXCL14 WWC2 SEMG1 TGFB2 MPPED2 CTD-2251F13.1 TRAF3IP2 MDFIC MEF2C-AS1 CHRDL1 CHRNE EPB41L4A RP5-855F14.2 SNORD113-5 DOC2GP MAATS1 LZTR1 RP11-108L7.15 TRIO MEX3B ZNF385D NID1 FBXO16 RP11-654A16.3 MMP13 ZNF85 ITIH5 ZNF473 C8A RP11-487E13.1 CSNK1A1L FAM127C AC106801.1 HOXC13 CLGN OR10G9 OR5AS1 RP4-555D20.4 UCN OR10AC1 MMRN2 AF186192.1 STX18-AS1 COL20A1 RP11-1000B6.3 OR10D3 PTPRE RP11-144F15.1 LINC01150 ZSCAN5A UNC5B FAM20A RP11-216L13.16 NUDT10 UGT3A1 MSN MFSD6 CKLF-CMTM1 SH2D1A EPDR1 CROCCP1 MYO3B SLX1B SH3RF3 BPIFA2 RP11-410N8.1 ALKBH3-AS1 RP11-31I22.4 TBC1D31 RP11-876N24.2 ZNF347 RP11-510C10.2 PKD1 FOXR2 KIAA2022 KCNIP3 GPNMB TRPM8 GPR83 RN7SL526P PCBP1-AS1 KIAA1522 PSG8 GFRA4 CDK15 RP11-923I11.5 ZSCAN30 RP11-218D6.4 OR4C46 CLN8 AC004490.1 PTPRZ1 ZNF454 RP11-136C24.3 MAP3K5 RP11-38C18.2 CLDN11 HLA-H RP11-2E11.5 ARPP21 STAG2 HRK ODF1 CTC-459F4.3 SPAG16 Six3os1_2 MIR1283-2 DSCAML1 NFATC1 GLUD2 FBXO27 SCOC SLC18A3 INTU SYNGR3 TRBV24-1 EML1 SMAD3 NUP93 RP11-662M24.2 TBX5 APCDD1L AP004782.1 FBXO22 MRVI1 OR7E104P SHISA3 RP11-427J23.1 GJB6 ASB8 MEPE OR10J5 ANKRD10 FOLH1B C1orf101 RERG SLC16A7 HTR2C OR56A1 GS1-24F4.2 GALNT13 ZNF43 CCDC141 OR5AU1 ITGB4 CACNA1E SYT10 OTOR DRD5 CFAP61 PAQR6 CTD-2314G24.2 MAGEE1 AC007682.4 CMTM1 MAGI2-IT1 TBC1D5 CNTN4 CTIF LCE1B MIR1253 LRRC38 RP11-370K11.1 ATP11A RP11-145E5.5 RBM24 MUC16 ERVMER61-1 RP11-45D17.1 MYO1C VCAN-AS1 RP11-649A16.1 LINC01159 KRTAP7-1 SYT5 CNKSR3 BTBD17 SPRN POU2AF1 ZNF648 OSMR ZNF574 VWA8 CYSLTR2 DRAXIN RP11-650J17.1 ZC3H3 C19orf25 NLRP6 TMC2 RP11-2N1.2 LINC00925 RNF17 PCDHGA7 TRIM72 RP11-473J6.1 CKB DBX2 RP11-39M21.1 TRBV28 AC133141.1 INSC RP11-3B12.3 RP11-586D19.1 RP11-371A19.2 PARVA AC106782.20 JAKMIP2 MAP3K14 RP5-998N21.7 PTHLH PKLR TMCO5A CCNI2 TTLL1 PRKG2 TLX1NB C8orf48 LMF1 SLCO3A1 RP11-529J17.1 AC133680.1 C9orf50 TRBJ2-7 HDGFL1 PAG1 RP11-753H16.3 CAPN14 PAX9 NEFM AC007040.11 XXbac-BPGBPG24O18.1 MRPL28 AC108044.1 SLC6A1-AS1 TRAF3 CXCR2 RIPPLY3 APBB1 PCP4L1 SND1 MPPED1 LIPE RP11-541P9.3 ERICH1-AS1 LINC01391 MAN1A2P1 SNAP25-AS1 EMBP1 HMCN1 NREP-AS1 DPYD-AS1 RP11-68L18.2 ESYT3 MIR181B1 RP11-30J20.1 CD40 FAT2 LY86 WHAMMP2 RP5-947P14.1 GDI1 OR4C16 ZMYND8 AC016910.1 TCHHL1 RP11-319G6.1 HCG17 RP11-168P13.1 IGFBP7 ATP6V1G3 NACC2 ZNF382 ECE2 RP11-401O9.4 WTIP EPHA5 RP11-358M11.4 RP11-366F6.2 MYOT CTD-3035K23.7 POU4F1 C3orf20 AC004692.4 SGIP1 ARL4C CTC-523E23.15 UBQLNL ATG4B RP11-1042B17.3 GALR1 FBRSL1 MERTK GRIN2C COLGALT2 SNORD115-36 AEBP2 SLC47A1 LINC01231 ISL2 RNF144A CST1 EMC3 SLC7A14 SNORA24 MCHR2-AS1 LHX5 CCL7 AC004538.3 TP73 SH3RF3-AS1 TRPC7 LPXN CTD-2517M22.14 GRIK4 TRIM65 SERPINA1 COL4A3 SLC6A18 RP11-626H12.1 OLFM1 TMEM74 RET RUNX1 XXbac-BPG248L24.10 FADS2 ALX3 RP11-420L9.5 GPR64 LIPH ALDH1A2 LINC00535 DKFZp434J0226 GABRG1 RP11-637O19.3 MYO16 TSHZ2 LOC440461 RP11-433J8.1 ZNF543 ESRRG RP11-381K20.2 RP11-594N15.2 ZNF540 OTX1 EFNB3 STON2 GLP1R RP11-193H5.1 RP11-168L7.3 MIR7515HG STOX2 CDR2 MIR124-2 ZIC4 SLC25A1P3 PROKR1 ABCB1 DLG3 GPR12 RP11-576D8.4 FAM171A1 AC079610.2 RP11-2C24.7 BATF3 RP4-742J24.2 PCLO KLF6 USP54 CTD-3065J16.6 RP11-169K17.4 SLC25A21-AS1 RP11-657O9.1 TM2D3 SCUBE1 NOL3 SLC32A1 ARNTL2 TRIM49D1 DCD BHLHE22 IL17A AC000403.1 AQPEP CPA2 HDGFRP3 LEPR LTBP2 HECW2 MIR663A PKD1L2 RP11-326I11.4 NBPF2P LSMEM1 BTBD16 SLC43A3 PGLYRP2 ZNF502 GP6 INS-IGF2 SLC25A22 AC096558.1 EPS8L1 RP11-834C11.5 PITX2 MYBPC3 GJA4 TOB1-AS1 GTF2F2 WDR17 SIM1 PPP1R42 SATB2 KCNJ9 ZFHX4 BLID ADAMTS19 KIAA1217 C22orf42 ABTB2 LINC01361 AL021918.2 COL6A2 FZD7 AC131951.1 TMEM132C ANKS1B PATE2 CHL1 TDGF1P2 SDC4P TIGIT NCCRP1 KLHDC8B RP4-576H24.4 STOM SPOCK3 RP11-115J23.1 EGR2 PLEKHA3 FRMD6 RP5-921G16.1 DGKZ GLB1L2 UNC79 RP11-115N4.1 SPON1 ANK2 SH3GL3 AP000345.2 MIR100HG VLDLR BOLA2 CERKL EXOC3L2 HSPD1P7 LINC00092 MAP2K2 SLC1A2 AC106786.1 HIST1H2BB ZBTB16 ELN RFX1 VIM CHIAP2 STXBP5-AS1 ANO2 ZEB1-AS1 TMEM209 CTD-2308N23.2 RN7SL68P NGB RP11-400N13.1 OR2AK2 OR9G4 MAML3 RSPO4 RP11-321E2.12 GABRB2 SLIT3 RP11-276H7.2 MAN1C1 RP13-578N3.3 KRTAP13-4 ADAMTS14 XKR5 DYDC2 TEX28 NR1H2 SHANK1 ARHGEF39 AC025165.8 PHC2 DBX1 PPP2R5C OOEP ST3GAL6 DMBT1P1 CTA-360L10.1 AC093787.1 ZNF578 TDH SNORD114-21 ANKRD30BL RUVBL1 AE000661.37 MAD1L1 NKX2-1-AS1 GPR176 MYH11 DPYSL5 AC004899.3 ROR1-AS1 ANKRD11 PTCHD2 EBF3 NPAS4 NT5DC3 DDAH2 EMX2OS GUCY2D EGFLAM INSM2 FGF4 RP11-630D6.5 C5orf49 SLC26A4 PLD5 RP11-138I17.1 IRX1 OXR1 PAK3 DPYS MRC2 CTBP1-AS AC116366.5 RP11-158J3.2 DPPA3 RP11-1C8.4 AC007680.2 GSDMC SFMBT2 RNA5SP175 HEXB EBF1 RP4-712E4.1 CRLF1 ABI3BP AC008781.1 ATP1B2 C8orf22 NKX6-3 RP11-377G16.2 LINC01314 CDK5R2 MIR7154 RP11-478B9.1 TUB KLF12 RP11-295K3.1 PASD1 FAM69C HSPA1A ITM2C EPB41L4A-AS2 SCAMP5 NEUROD1 RP11-444E17.6 SENCR LINC00473 WBSCR17 GNAS-AS1 CDH6 PCDHGB2 RIMKLB ZNF134 MYL1 PPIB ANKRD36BP2 CALY MTERF1 UGT1A4 CTD-2034I21.1 SSUH2 OVOL2 PPP1R10 RP11-68E19.2 MIR376B CRYGN OR5AN1 PTX3 CENPN OR10K2 SPANXB2 TMEM106A C3orf80 SCGB3A1 UGT1A6 POM121L2 OR5AC2 DOK6 GPR149 C8orf88 DFNB31 VCX FCHSD2 LL21NC02-21A1.1 SERPINB9P1 IFNL4P1 CCDC40 ECEL1P3 ZNRF2 NEXN-AS1 TRBV30 CACNA1F HERC2P4 LINC01122 PCDHGA12 GRAMD4 FAM90A14P GJB7 DNAJB6 NAGS NFIC GABRR3 PRKAG2 BRINP1 OR6K3 RP11-53M11.5 RFX8 C8orf34 LINC00158 SLC35C2 ABLIM2 TCF24 RP11-17E2.2 IGFL2 HAVCR1 SULT4A1 SHC3 WASF3 RP11-435M3.2 SOX9-AS1 AL662800.2 MMEL1 C1orf140 SIRPD AC018866.1 CPQ HOXA7 AC096669.2 HOXD1 TRBC2 CPEB1-AS1 RP4-665J23.1 FFAR1 C17orf82 KRTAP21-2 CHODL CTD-2316B1.2 TNFRSF12A KRT6A CTD-2194D22.4 EGR3 ORAOV1 PTF1A MSANTD3-TMEFF1 BX842568.1 PPP1R3A MIR4437 PABPC4L RP5-906C1.1 CTD-2194D22.3 PRRT3 KIRREL3-AS3 BARX1-AS1 RP11-543D5.1 ZSCAN1 RP11-1191J2.5 FBXL8 LINC00237 DFNA5 FGF13-AS1 EIF5AL1 IL6 FGF14 PTPN7 RP11-89K21.2 PRICKLE2 STATH FBXO44 IGJ AF186192.5 TRIM51EP GLRA3 SPRED3 RIPPLY2 MYADML DMRTA2 BCL7C RP4-559A3.7 PSTPIP2 CTD-3195I5.3 LINC00861 OR51M1 GAPT ISG15 DHRS9 LYPD1 RNF165 ZCCHC14 ANP32C ASB15 RP11-523O18.5 CACNA2D3 ZNF831 CCRL2 RP4-799P18.2 DGKK ZNF461 RP4-539M6.14 SLC7A2 OR51I1 CLK1 MAGEH1 TAS2R16 NPY5R OR10S1 ITGAM RP11-978I15.10 OR5P3 CCDC149 RP11-661P17.1 L1CAM SH3RF2 OR51L1 MST1R DLL3 CREB3L2 CNTN3 TIGD7 RP11-758M4.4 GFRA3 MGAM COL3A1 PTMA RP11-290F24.3 GLIS1 PRDM11 RPL26 GOLGA8M KIAA1324L OR5A2 LINC01060 CACNA1D PRKAA2 LLfos-48D6.2 ELOVL2-AS1 IVNS1ABP ZNF529-AS1 C1QTNF4 RP11-964E11.2 RP11-799N11.1 CD37 RGS5 BTBD3 ARMC3 PRKAR2B PRDM8 MAPT-IT1 OPLAH PACRG IL1RAPL2 CCDC154 EPPK1 SLC8A1-AS1 CLSTN1 SPAG17 RP11-9L18.3 MUC19 P4HA2 EDIL3 FUT1 SEMA5B RORB-AS1 B3GALT4 LINC00977 MIR205 TMEM155 RP1-28O10.1 TMEM51 MIR4425 CC2D2A RCCD1 TBX4 BTN1A1 CMTM2 LHCGR DUSP8P5 VWDE TPRG1 SIRPAP1 TRIM67 LSP1 CHP2 LINC01059 KLHL33 AP000797.3 LRRTM4 ARHGAP18 CTD-2535L24.2 PTPRN2 CLDN5 LINC00710 MYOM2 CCDC140 BST2 ONECUT1 LINC00395 CYP26C1 AC012501.2 OR2A12 LYN BPIFA1 DUSP6 RP11-141M3.6 NAP1L3 GMPPB KLF7 RP11-662I13.2 B3GALNT1 EAPP REXO1L6P TCP10L MYO3A AC004980.8 DMKN RP11-712B9.2 DCDC2C ADAM33 TCN1 NTMT1 C17orf104 HIVEP2 BMS1P4 NUDT11 RP13-452N2.1 FAM65B B3GNT4 OR9A4 ICAM4 SYT6 C7orf66 LYG2 NOD1 SOX5 CDH22 KIF6 OTX2 SFT2D3 DSCAM IL12RB2 PERM1 NFIX CLEC14A LLNLF-65H9.1 RNASE3 HELT PIK3CG RP11-255M2.3 HCG4P8 RP11-402J6.1 CD5L COL4A4 RP11-993B23.3 IL17REL GJA3 RP11-173E2.1 AL928742.12 TBC1D30 AADACL3 HOXC12 CDK2AP1 LAMP5-AS1 OR6K2 NODAL FFAR4 GTF2A1L LHX3 GRID1-AS1 RP11-110I1.12 OR5D16 SRRM3 GINS4 SLITRK5 S1PR1 RP1-269M15.3 SKOR1 CSAG4 GIMAP7 SYT3 HCG4P7 CTD-3234P18.6 ATP6V1E2 RP13-143G15.4 RP11-646J21.7 RP11-410N8.4 HOXC9 DAB2IP SLC6A5 GRIK5 FGF14-IT1 DAZL RP11-255G12.2 C5orf66-AS1 SDK1 HAND1 C5orf66 LINC00602 SYK GSX1 CALHM3 FREM3 FUT9 RP11-242F24.1 RP11-430H10.1 TBR1 OTOS PRR32 ZNF773 AF038458.5 CCL8 SLC12A1 SLC6A19 NAT16 ENTPD4 CTD-3092A11.1 RP1-293L6.1 AC079154.1 TUBB6 KCNN1 FOXL2 CCDC80 KCNQ2 LINC01105 RP11-304L19.4 LEMD1 HTATIP2 AC092675.3 GRM5 AZGP1 C2orf71 PAMR1 FXYD7 PHYHIPL EPHA6 CXCL12 TFAP2C NTN1 PCAT4 SPRR2A RP4-683L5.1 LINC01442 FBXL21 CA10 RP11-383H13.1 COMMD10 GPR50 SIL1 LDB2 NR5A2 IGF1R JSRP1 RP11-13K12.2 EHD3 ISCA1P1 KLK5 MIR124-3 PURG EFEMP2 RP11-208N14.4 A1BG PKD1L1 NLGN1 TSNARE1 RNU6-803P DNAH9 TNFRSF1B TNS4 NAV1 IQSEC1 AEBP1 SHISA9 RP11-92A5.2 CCL28 SHROOM3 IRX2 AC005597.1 ZNF256 CH17-270A2.1 MSI2 AC112198.1 OR4K17 AC025280.1 PLEC LRRC7 VWA7 TEX41 LINC00458 ANK1 DCP2 SNORA80B PPM1L GJD2 CASZ1 ATP4B RP11-431K24.3 APCS KRR1 SAPCD1-AS1 PROB1 SNORD115-42 SNHG8 C6orf10 EXD3 PDZRN4 CARTPT CNTNAP2 USH1G PPAP2B SDHAP3 OPN1LW PAX8 MIR503 CTD-2623N2.5 BEX1 TRIM71 NR2F1-AS1 SPECC1L-ADORA2A RP11-1E1.2 LCE6A WNK3 BIRC8 FEZF2 LY6H RP11-586K2.1 RP11-522B15.3 MAP2 TRAC OR2L8 LIPE-AS1 MIR1-1HG AQP4-AS1 TMPRSS11F PANX2 KCNIP4 RP11-424D14.1 SOX2-OT FAM43A CFTR CAMK1D FCRL4 KLF14 OR4C15 KCNA5 MIR6720 DYNC1I1 FLRT2 RP11-15B17.1 WRN STAU2-AS1 Six3os1_3 NALCN-AS1 RIT1 CCDC144NL LINC01208 CTD-2335A18.2 RP4-815D20.1 SPON2 C12orf79 OR2G3 NKX2-4 RP11-379F12.3 ACSF3 TRBV27 RP11-131L23.1 BRINP2 RP11-298D21.1 SLC12A5 RP11-526A4.1 NRIP3 NOS1AP TMEM121 RP11-426C22.4 PAUPAR TEX26-AS1 NKX1-2 LRRN3 EFTUD1 LINC01392 TRIM49D2 PTPRO GPRASP1 MAP9 CXorf36 AQP1 AC061961.2 TCERG1L GFAP FAM133A ZDHHC14 AC024560.2 C6orf118 ZFP64 AC011752.1 RP11-554D15.3 LYNX1 XG KIF13B PCDH9 RP11-92C4.6 RGAG1 LINC01029 RP4-753D10.3 ZNF185 C22orf34 TTYH1 LHX5-AS1 ARHGEF33 DNASE1L2 LMO3 HMGCLL1 RP11-570L15.1 NEUROG1 GLB1L3 HIST4H4 NKAIN2 XKR6 ATP13A5 OR5AR1 LINC01053 RBMS3 MIR1283-1 RP11-459E5.1 DSEL C7orf31 MOS CD1E OFCC1 C9orf170 RP11-95M5.1 MIR646HG TDRP FAR2P2 AP002884.2 CTD-2554C21.2 ANGEL2 NHSL1 AC007796.1 RP5-998N21.10 DYDC1 ARHGEF6 TGFBR2 MIR205HG RP11-169K17.3 LINC01099 OR4K5 GLIPR1 GLRB SNORA71E MSLN ITGA11 RP11-166D19.1 WWTR1 MARK4 CRACR2A RNF212 PPP1R16B AC074183.3 TTC23L RP11-1166P10.7 PGC FGF3 TNFAIP2 AMOTL1 RP11-844P9.2 OR2T3 LINC00668 CTD-2561J22.3 ARHGAP6 RP11-434I12.2 KIF28P SERPINB10 CT45A1 AC096570.2 TTC6 PPP2R2B ARHGEF17 CHD5 ELF5 VSX2 RP11-329E24.6 IL12A-AS1 RP11-10A14.5 ZFR SCUBE2 RPS6P8 GPX5 VANGL1 DAOA MMP25 RP11-710C12.1 Y_RNA FABP4 NRCAM KCNB1 RADIL PATE3 ZMIZ1 DAP ENPP6 RP11-229O3.1 LA16c-390E6.5 LINC00298 TEX13A OVCH1 COL6A3 APOBEC3G TMEFF1 RP11-106M3.2 RP11-619I22.1 LA16c-306E5.3 RP11-774D14.1 CNN2P10 MYPOP CRTAC1 Z95704.3 HSD17B12 RP11-390N6.1 RP11-191L9.4 CCDC85A ZFHX3 CYP2B6 EGLN3 HS6ST3 OR5H2 PITX3 OR52E8 RP11-358H18.2 SHISA2 GRM4 LINC01551 RP11-272L13.3 LINC01010 RP11-770J1.3 OR52N2 ENC1 SPACA6P-AS WDR86 KCNMB2 CTD-2555C10.3 SIX1 TIMP2 CDH12 GPR156 AC021021.2 ZMAT4 OCM GPC6-AS1 RP11-133F8.2 LINC00578 ZNF19 OR11A1 MIR520D TMEM132B BASP1P1 AC007228.11 FRMD6-AS2 SGCE ANKRD34A GPRC5B ZSWIM5P2 TCF7 SAMD9 RP11-24F11.2 HSD11B1 KITLG FLG-AS1 SV2C AC009120.4 CACNA2D4 GS1-594A7.3 LHFPL3 KCTD12 MAGEB4 ZEB2_AS1_3 RP11-510C10.3 DLGAP2-AS1 COL5A1 RP11-348J12.2 RP4-764D2.1 ZNF662 FAM209B TNFSF9 KCTD21 AC079781.8 PPFIA4 CNIH3 AC007879.5 DOCK3 GNAQP1 PAQR9 THSD4 LRRIQ4 AC003984.1 RP11-307N16.6 RP11-129J12.1 LMNA KRTAP21-1 TBCD EFCAB1 MIR3074 KPRP PRICKLE1 WNT5B PEG10 TAS2R38 INHBA-AS1 TEPP RP11-742D12.2 TTLL8 APLF SNED1 TTN-AS1 RAET1E RP11-49I11.1 WNT9B RELN NKX3-2 LINC00284 HAS1 OR51B5 RASGRF2 TRPS1 HS3ST4 POPDC3 ATCAY RP11-91I20.1 RP11-541G9.1 IGFBPL1 MFSD9 LHX2 LCE3C PCNXL2 B3GALT1 AC092675.4 PTH1R RBP1 ZKSCAN7 ADAMTSL3 CALHM2 CTC-321K16.1 CRISPLD2 FBXO32 SNHG14 PCDHGA6 C8orf37-AS1 SLC26A4-AS1 NXPH1 ZRANB2-AS1 AR AC002368.4 RP3-370M22.8 THRAP3P1 FAM90A28P RASA3 PLXDC1 CDH4 RSPH9 GAD1 SERPINB12 RP11-371M22.1 RP11-272P10.2 GABRA3 RP11-452N4.1 FJX1 AMELY RP11-153K11.3 LINC01398 OR51A7 FGD6 TRHDE-AS1 RP11-266O8.1 TNR USP44 ADCY9 DPP10 ATP6V1B1 NOTCH1 AC018730.4 CNTFR-AS1 RP11-221N13.4 RP11-20I20.2 OCA2 SNAP91 PPP4R4 CELF4 CDKAL1 PDYN VAV3 DAAM1 ATF4P4 AC016735.2 SCML4 AC053503.11 ARHGAP22 SSTR4 TNFRSF10D ULK4P2 NPFFR1 RP11-259O18.4 UGT1A3 INPP5A PCDHGB1 HCG20 UGT1A5 RAB6C-AS1 LINC01362 ADD2 CNTN6 CLIC6 RP11-379P15.1 PYDC1 NPTXR AC107218.3 TUBB8P6 HCAR1 SCN9A LINC01375 TTC7B DPYSL3 GPC1 UBE2Q2P1 TEN1-CDK3 RP4-555D20.3 MNX1 OR10A7 ELMOD1 MS4A14 IGF2BP1 RP11-395B7.4 AC138649.1 RP5-1031D4.3 RP1-301L19.1 HLX ELAVL3 PTGIS CTD-2297D10.2 MAMLD1 ZNF200 RP11-464F9.1 SLC25A53 FBLN5 RP11-390F4.6 ZNF788 HAPLN1 MIR490 SNCB CCDC88A MIR551B CTD-2619J13.19 C10orf11 AC005336.5 AC010907.5 SLC16A12-AS1 ARNT2 MIR663B KCTD1 AMZ1 C5orf17 HOXB-AS3 NRSN2 KCNA7 ZNHIT6 SPOCK1 SPEF2 CXorf22 RXRB ANKRD45 AC133785.1 EIF2B5 GRAP2 OR4D1 GAPDHS MUM1L1 CNTNAP1 GOLT1A CYP26A1 RP1-161N10.1 TACC2 MGC16275 AC007131.1 ZNF492 RP5-945F2.3 RP1-170O19.17 AC092162.1 U2SURP RP11-786A20.1 CUBN CRTAM ADAM12 IGF1 ITPK1 RP4-769N13.7 BCAR4 POLK ADCY7 CCDC177 PPP1R14A TRBJ2-5 RP11-368M16.7 DAZAP1 GNAL B9D1 NKX2-5 LPO MITF PLEKHA3P1 AC021021.1 RP11-561I11.4 PEBP4 IGF2BP3 HOXD11 CACNA2D1 STK32C AC005027.3 SH3BP4 EGFLAM-AS2 CLUH CTB-147C22.9 ANKRD13B ZNF131 CTC-276P9.2 CCDC88B ARRDC2 HCN1 TMEM235 AC013733.3 FGFR1 FXYD6 RNU5F-1 MTNR1B TET3 AC004041.2 MIR5691 UNC5A IGHD2-21 CTD-2619J13.8 MAGEB3 LINC00693 AC008060.8 LRRN2 RP11-753H16.5 ZNF582-AS1 OR5D14 B3GAT1 SUSD2 FRY RP1-140K8.5 RP11-252M21.6 FADS1 AC005498.3 DKK2 UTF1 LBX1 OXT NTRK3-AS1 B4GALNT1 TANGO2 ARL4A IRX5 CTD-2384B11.2 PTPRG-AS1 IGHG4 LIMS2 AC097468.7 MIR129-2 TRAV1-1 BAHCC1 BSN RP11-124G5.3 CTC-525D6.2 SMO BVES-AS1 PIK3R5 GLT8D2 COL9A2 CNN1 KRT1 GPC5 SDAD1 AL122127.3 TRAV24 TLR1 OR5AP2 AGBL4 TENM4 CPNE5 INTS6 ZCCHC5 CTD-2020K17.1 SEC14L5 TMEM240 CLTA CCDC106 RP11-587H10.2 OGDH WHAMML1 MACROD1 CECR2 RP4-575N6.4 FIGLA TEKT1 HSPA1L LYPD4 SSTR2 C10orf67 EPHX3 RFESDP1 PRR16 ZNF32-AS3 UTS2B C1orf132 ZNF529 AARD DDX53 GHR RP11-345J4.5 DYSF CLEC4E SCUBE3 AC074389.5 MTRR PLEKHO1 HTR7 RP11-689K5.3 RIPK4 MIB2 WDR59 LINC00608 HIST2H2BB A2ML1 MSMB LMOD3 C3 CASC8 TRIM41 PRDM16 FOXI2 RP11-714M23.2 CENPVP3 RP11-666A20.3 INSRR SLC38A10 AC096559.1 SIPA1 DMRT3 TRIM2 GPR75-ASB3 MIR34B RP11-123K19.1 CTC-512J12.4 DLX6-AS1 KRT26 UGT1A10 ATOH8 GLTSCR2 RP11-1007I13.4 RBFOX1 FAM163A MEIS2 STARD13 PCDH7 RPLP1 ZNF75A SLC7A3 CDH10 RP11-147C23.1 RP11-356J5.13 LRRC4C NKX6-1 IFT140 RP3-335N17.2 SBNO2 FAM162B RP11-609D21.3 TRIM29 CTD-2600O9.1 RP11-372M18.2 RPL13AP17 RP11-106M3.3 AC008686.1 CCDC185 BCAM TREML5P ADAM19 OR52D1 RP11-455F5.3 AC003986.6 GCK NLRP10 PNLDC1 CTD-2269F5.1 ADAMTS16 SCP2D1 EDNRA PAQR9-AS1 ZNF331 C7orf62 PDE4C LINC01090 PPFIA3 RP11-600K15.1 ZNF334 WT1-AS RP5-906A24.1 RP1-27O5.3 MIR920 AC004987.10 POM121L12 FOXB1 AC055876.1 HHLA2 GMFG PIK3AP1 LTBP1 RYR1 FGF8 RGL1 MT3 DLGAP4 LRRK2 CTA-392C11.1 CCDC181 NKAIN3 CPEB1 VN2R1P ACOT7 ZNF395 FGF6 CNTFR PCDHGA1 RP11-106M7.4 RP11-415D17.3 WNT3 WT1-AS_1 ZNF467 ZNF549 SLC38A4 SLC25A21 CTD-2330K9.3 PALM2 RP1-34H18.1 DPPA5 CLSTN2 RP11-35J10.5 LGI4 EPHA1-AS1 C20orf78 MFAP5 RP11-114H7.2 OPRK1 FENDRR RNU6-1103P RP11-152L7.2 AF131216.5 TEN1 AC002480.2 VASH1 NDUFC1 SLC22A16 RP11-688I9.4 IGFBP7-AS1 PKHD1 RP11-379F12.4 MEG8 XXyac-YX65C7_A.3 CTD-2509G16.5 AC007392.3 RARB GDNF-AS1 AC091878.1 DIRC1 MAFA IMMT CNOT6 FIRRE FLJ46284 HLF SNRPGP16 ADAMTS2 MYH13 NPHS1 YIPF7 PKIB HOXD13 C15orf59 TCF4 CTD-2291D10.4 GABRQ SPANXA2-OT1 MYH1 MGST1 ADARB2-AS1 TBX2-AS1 FRYL RHOBTB2 AP002856.5 GPRC5C DCSTAMP OR13D1 PNPLA6 RP13-580B18.1 IGLL1 KLF2P2 RP5-850E9.3 CPAMD8 TIAM1 FAM154B RP3-333A15.2 PBX1 CTD-2666L21.1 CTB-35F21.4 MMP20 PLEKHM1P CTD-2012M11.3 RBM38 PHACTR1 MTSS1L PWAR5 SORCS2 MEGF10 NR1I3 PRRX1 MYOD1 ACSF2 SCN1A GNAO1 AC079776.2 MMP23B HCK CTD-2537O9.1 WNT3A CSTA MAGEB16 DISC1 GABBR1 TRBV25-1 USP4 TMEM25 MIR890 BMP6 PAX8-AS1 NPHP3-ACAD11 LRTM1 RP11-1E11.1 RP11-23P13.6 CFAP58 LCN1 RP11-304L19.1 MIR485 SLC16A1-AS1 CCAT1 PHF21B SLC17A9 TRGC1 ZFPM2 TDRD10 PIEZO2 GC SLC35F1 VSTM4 RP11-473C19.1 SNTG2 GRWD1 BCKDK AC147651.5 CMYA5 PXN SVEP1 AC060834.2 NTM RP11-161M6.5 RP11-667M19.5 PCBP3 RP11-46H11.12 BFSP2 ZNF354C GRM6 NGFR FRMPD4 RP5-955M13.4 LMCD1-AS1 HPYR1 MEIS1 PCBP1 RP4-584D14.7 OR13H1 NGFRAP1 MAGEC1 AMMECR1 HAGH AC098617.1 OR10T2 TMEM179 FBLL1 PDGFD CACNA1H ERCC1 LCE2D KCND3 RP13-16H11.7 CTD-2281E23.2 TRIL KRTAP6-2 NKX1-1 BVES ZNF254 LINC00629 AC105402.4 PAX1 WBP1 BRINP3 ABCC2 RIMBP2 SGK1 COL5A2 DNAJC16 PRMT8 LINC01237 TCEAL6 MIR3652 CCDC169-SOHLH2 RP11-175K6.2 AC091633.3 RP4-537K23.4 FGF14-AS2 BAI1 RP11-632K20.7 BRSK1 Vax2os1_3 THY1 AC002456.2 CTAGE14P ZNF518B RP11-1084E5.1 EPHA7 DISC1FP1 SCN4B MST1P2 ZNF439 ANTXR1 NSMF RP11-966I7.2 C7orf49 KRTAP1-5 ATG7 OR8K1 MIR1911 OR52A4 STK24P1 RP3-468B3.2 LINC01586 FBN3 HS3ST6 CTD-2278I10.6 CASP14 GRIA1 HOXA-AS3 HCG4P3 FAM19A1 PLAC9 ZNF627 WNT5A Six3os1_4 MTUS2-AS1 TRPA1 RP11-317P15.4 AC003002.4 TRGV10 CTD-2135J3.3 C1QTNF7 RNU6-923P CCDC90B LRP5 CTD-2655K5.1 RASAL3 RP11-849F2.7 NEFL UBA6-AS1 GALNT2 SNORD115-23 RP5-1065O2.4 NAV2 RP11-482H16.1 FLJ33360 ATXN7 RP11-200A13.1 IL21R STK3 RP11-678G14.3 ZNF781 FAM78A RNF150 PRDM6 EPHX4 DNMT3A TMEM229A MIR1204 MIR6090 COL19A1 BAG3 CA5A CCND1 RUNX1T1 RP11-162J8.2 EIF4E1B LRRC61 PIK3C2G RP11-462G12.2 HNF4G MSR1 CCL4 SNORD115-40 TRPM2 MEIOB RP11-394A14.4 LAMC3 BMP2 TOX NPY1R C19orf81 PCDH15 SYT7 GCNT2 GABRG3 PIGBOS1 SLCO1B1 SST AC011453.1 OLIG1 RP11-690J15.1 RP11-626I20.3 ZNF790 CTD-2576F9.1 SGCZ ACE2 TCF15 RP3-525N10.2 OLIG3 RP11-203I2.1 CILP2 PRPF38AP1 EDA2R MIR889 FAM107B IRS2 DENND2A LUZP2 RP11-823E8.3 KCNAB1 TRIM42 CTD-2126E3.3 BMS1 MXRA5 GRM1 PDE4D RP1-63G5.5 BPIFB6 EPHB3 RAMP3 CNTN5 TSPYL5 RBMS1P1 GAP43 RP5-1186N24.3 C7orf72 ERC2 POFUT2 C20orf166-AS1 PRDM13 RP11-19E11.1 OR6Y1 PCDH11X OR10A6 RASGEF1A CAND2 RIMS1 RCN1 TEX14 AMIGO3 DEFB121 TMEM225 FCRLB RP6-24A23.6 MIR4435-1HG TRUB1 ENPP2 PIK3CD SLC7A11 ACAN FHL5 CTD-2521M24.9 SHISA8 PPM1E RP4-728D4.2 ZIC3 PAX2 AC114765.1 CTA-331P3.1 CHST2 AP000857.3 TRIM51 ZNF530 RUFY1 FZD10 FRMD4A BIRC2 TNFRSF8 BCL11B LMX1A EVX1 WASF3-AS1 PRAC2 RNA5SP243 EGFEM1P RSPH3 LINC00391 WDR86-AS1 KLF9 AC062021.1 PCSK2 AC010127.3 KCNIP1 TMEM178A RP11-360L9.7 OR5M3 AC011306.1 TM4SF1-AS1 IL13 CD86 RP11-123O10.3 FOXN3P1 ASAH2B PIP5K1C ZDBF2 CAHM RP11-748H22.1 POU2F2 TSPAN31 ZFPM2-AS1 GPT CLIC1 CTD-2277K2.1 CNKSR2 CSAG1 BEND4 GFRA2 SNURF FILIP1 KIRREL3-AS2 SAMD5 LINC00621 BEND5 IGFBP5 COL8A1 RP3-399L15.3 CXCL1P EPHB1 RP11-805J14.3 SSBP2 MMP12 HS6ST1P1 OR8B12 COLEC12 GABRA1 MEGF6 LPHN3 LLNLR-304G9.1 BMP10 LINC01351 RPH3A LSAMP RP11-642P15.1 AC007906.1 KCNQ1 ZIK1 CTD-3032H12.2 PROCA1 APOF NLRX1 RP11-805I24.3 GREB1L HAGLR BSG LINC01088 KCNH1-IT1 RP11-613M10.8 PDE7B OR4A16 EFEMP1 ARMC2 BIN1 PIGB LINGO3 PCSK1N RPS15AP10 IGSF21 LINC00402 PCDHB6 GALNT15 PTPRC OLFM2 FATE1 LNX1-AS1 EEF1A2 RASSF8-AS1 CCDC74A VWC2L BEST3 HRNR ABCC9 TRIP6 CAPN8 CDH3 ZNF727 BPIFB4 GSG1L LINC01098 RP11-114G22.1 GREM2 RP11-157I4.4 ARHGAP20 DOCK1 NR3C2 MIR134 MTUS2 VASH2 RP11-650K20.3 IHH FOXD2 OR6P1 TPTE2P1 MDGA1 COL9A1 WNT1 TMEM178B SOGA1 COL4A2 EVX1-AS SOD1P3 AC068490.2 PPP1R13L SLC1A6 TCF7L1 GFPT1 BRD7P5 PAGE1 GGACT SLC27A6 C9orf129 FAM126A CTB-41I6.1 INS RP11-432M24.4 RP11-511B23.2 AC012668.2 RP11-93K7.1 HOTAIR CLEC4D AC007131.2 ZNF528 ANKUB1 SMAD2 VSTM2B EFNA5 NOL4 RP3-455J7.4 NXN AP000251.3 AC073316.1 MFSD7 FAM200A PRKCZ ZIC5 RP11-885B4.1 RP11-817J15.2 COL11A2 CNTLN RP11-448P19.1 CHRNA6 GATA3 OR52B2 PALM ZNF365 CMSS1 ARX IRX6 RP11-443N24.2 LINC01049 MPZ RP11-443B7.3 RP11-1055B8.8 SSBP3 PCDHGB7 AC017002.2 CYP4F22 AP001471.1 INSM1 C14orf39 TLN2 FOXC2 HDAC4 ZNF582 SYCP2L HS3ST3B1 GDNF RP11-425A6.5 MMD2 RP11-87H9.2 KLHL1 OR51B4 RP11-474D1.3 NEUROD2 C1QTNF9B GSTM1 CRH ACP5 ATP9A F11-AS1 RP11-396F22.1 GJD4 HOXA2 RP11-125O5.2 ZIM2-AS1 DKK3 TULP1 AF131215.8 CTB-33O18.3 AASS RP11-58E21.7 C19orf26 FLJ16779 C1orf50 RP11-806H10.4 ZNF793 SYNDIG1L CALCB LINC01197 VPREB1 LINC00290 PSD POTEF GYS2 IGF2-AS CT55 C8orf46 ADORA2A-AS1 C1QL2 IQCE HNRNPDP2 AC006116.21 HS1BP3-IT1 NKAIN4 TNFSF11 PTPRF KIF12 RP4-769N13.6 CASC15 PPFIA2 RFPL3S MYL3 THBS4 MEX3A AC074183.4 CMC2 ENOX1 MYLK RP11-26L20.3 PRIMA1 FCHO1 MSX2P1 RP11-434E6.4 HOXD10 RP11-934B9.3 FSCN1 CTC-276P9.3 RP11-9B6.1 LBX2 RP5-884C9.2 CTLA4 RP11-727A23.10 GCSAML WNT2 SIRPA MCM7 RP13-923O23.7 LYVE1 CSMD2 PDX1 LCN10 KRTAP20-1 UBQLN3 AP000997.3 FLG2 ACTRT2 WT1-AS_2 PDLIM4 WT1-AS_3 STEAP1B CLEC7A SLC37A2 DCBLD1 FAM132A TRDMT1 CFL1 UMAD1 LINC00299 RNU2-37P BAZ2B EML6 RP11-573G6.4 GCOM1 KRTAP19-5 CEP55 CTC-550B14.6 LA16c-306E5.2 TENM3 OPCML KRT20 AKAP12 CEMIP RP11-393K12.2 KIRREL2 HLA-DPB1 SFI1 AC114765.2 ANKRD60 OR52N5 GNE RP11-760D2.11 SLC22A31 CEP85L RP11-196E1.3 TUSC3 GABRD CTB-107G13.1 LINC00877 HSPA12B AC007563.5 RP11-136O12.2 FEZ1 UBXN11 VENTX PLEKHG4B DIRC3 ZNF880 NCKAP1L KCNJ12 KIAA1731NL PLCZ1 NLRP11 AC005754.7 CHRNB3 HLA-DQA2 F2R ADORA3 TUBA1C COL6A5 PIK3R2 LOXL1-AS1 MCHR2 KRTAP4-9 AC016757.3 RP11-190J23.1 EBF2 SMPD3 AC093323.1 AKR1B1 KAL1 RP4-712E4.2 RP11-355E10.1 GRID2 CALN1 HLA-G LRP2 SPTA1 SHB FOXI3 SEMA6D FGF12-AS3 RP11-517I3.1 KCNJ3 GPR113 ZNF573 TBX18 PCDHGA8 NOP14 NTF3 RP11-738B7.1 NKX6-2 OR56B1 OR5M10 IGHG1 GSDMB FOXB2 NID2 DNM2 CAPZA3 GLDC AC116614.1 RAX LINC01082 PDE6B CCL1 AC017002.1 DLGAP3 LACRT CXorf21 GATA3-AS1 ZNF735 OR1C1 FAM72A TMEM74B MCMDC2 WWC3 ZNF415 FZD8 ZNF853 RP11-513O17.2 LHFP CAMK4 SUSD4 GALNT14 ROBO3 RP11-834C11.4 RAB34 RP11-64D24.2 CCDC184 DEFB118 CPLX2 FGF5 LINC00466 RP11-157B13.3 IGHG3 ZFP2 VWC2 ANKLE1 Six3os1_5 GUCY2F COL16A1 RP13-726E6.2 REPS1 RP5-1027G4.3 CELF2 CD70 SUCLG2-AS1 NTNG1 RP3-471C18.2 RNU6-826P SETD1B ZNF606 RP11-523O18.1 TFAP2A C17orf102 LA16c-444G7.2 RP11-524N5.1 CRMP1 RP11-1102P16.1 PDE4B RP11-535M15.1 RP11-495P10.4 MYHAS ZNF804B MIR520B SNORD116-18 FBXL7 KRT7 MAFB NLRP4 GPR158-AS1 CLIP4 LRIT2 SLITRK2 AC009531.2 SGCD SMTN TMEM108 RP1-167F1.2 PKIA LINC01550 RP11-96H19.1 NKPD1 RP11-3J1.1 ZNF625-ZNF20 SEC14L6 RP11-40A13.1 GJC2 U91328.2 ENO1 GABRB3 RP11-61N20.3 GIPC3 GPR85 AC103563.8 AC012668.1 LCP2 MB21D2 ZNF772 SEZ6L ADAMTS3 MYZAP LINC01115 TBC1D7 GRM7 RP11-567M16.3 RP11-428O18.6 RP4-765H13.1 FFAR2 GLI2 MIR653 BTNL2 RP11-1021N1.1 SPP2 NAV3 MAP1LC3A KIAA1024 ZSCAN23 OTUB1 EMILIN2 SLC10A5 AC068134.6 RP11-632L2.2 RP11-283G6.5 RP11-1134I14.2 NLRP1 HOXA-AS2 UNC80 IQSEC3 PDCD1 OR5P2 ZNF512B IMMP2L CTD-2089N3.3 SPSB4 PALD1 ZNF469 KCNN2 KLHL34 EPHA5-AS1 SOCS5P3 C15orf32 ANKRD53 RP11-89M20.1 RP5-1120P11.1 PTPRM RP11-74K11.1 CCDC54 ARRDC5 ATXN1 SSPN TMEM59L IGSF9B SPSB1 KIRREL3 RP11-173C1.1 CHRNA3 CTC-444N24.13 FAM78B RP11-161M6.2 AC006033.22 MEI1 AK5 SYCN NDRG4 ELAVL1 CPS1 MIRLET7A2 TRIML1 SELV RP11-96P7.1 SLC35F2 IPMKP1 GALNT8 PCDHGB6 KIAA0226 C12orf56 RP11-550P17.5 RASSF8 RP3-438O4.4 PTCHD1-AS OR2A14 TCTEX1D1 RAB39A AC079630.4 RP5-965F6.2 NEK4P2 EIF4H DLC1 KATNAL2 SEPW1 RP11-783K16.10 DIRC3-AS1 BOC SEMA4A SSTR5-AS1 ANO8 LINC00944 MIRLET7BHG INHA WDR90 RP11-348M17.2 STX1B SHISA7 RGS17 WHAMM LINC01500 CLDN1 OR2T10 RP5-1121E10.2 ICOS PRAC1 ZNF671 NKX2-6 SPHKAP SPRR2G LINC01341 RP11-587D21.4 GDF1 ACTA1 SYCE1L ANKRD20A8P KCND2 RP11-214O1.2 LINC01250 RP11-395G23.3 WIPF1 SNORD114-31 LY86-AS1 ATP8A2 MIR663AHG FGF21 RAMP2-AS1 RP11-506B6.7 RP11-507B12.2 RP11-280K24.4 ZNF418 LARP4 AGBL5 ZNF503-AS2 BAALC-AS2 SLC24A3 LINC00599 C11orf96 C1orf228 GFRAL FAM57B NCAN TOP2B SOBP TSGA13 TMEM192 RP11-276H19.2 SHOX2 RIMS4 RP11-161H23.5 RSPO3 CPT1C TEX29 RNASE12 OR5H15 TPST1 RP11-189E14.4 DLK1 RP11-101O21.1 NOD2 HMCN2 CERS6 RP3-428L16.1 MARK2P9 RP11-65B7.2 HTR2A GLUD1P3 LTK RP11-1263C18.1 SCNN1G RP11-875H7.2 POLD3 RP11-439A17.10 TMPRSS12 TPH2 RP11-640N11.2 CCDC63 RP11-439A17.4 LPIN1 SNORD115-10 SEMA6B RP11-33A14.1 BAALC TNP1 JDP2 SERP2 ERRFI1 LOXHD1 RP11-233E12.2 CTD-2384A14.2 RP11-227F19.2 RP11-420N3.2 MTL5 LINC01158 PRPF40B ADNP2 ENO1-IT1 GNA12 RPL10AP3 LRAT PMCHL1 CTD-3032H12.1 MACROD2 TTLL5 SPTBN4 RP1-18D14.7 RP11-665G4.1 SPINK8 LINC00403 EPHA8 JAKMIP2-AS1 RP11-319E12.1 BEST4 DTX3 XXbac-BPG258E24.10 TIMD4 TMTC1 SUN5 GSTM2 EPHA10 ARMCX4 RP11-61O11.1 RP11-368L12.1 LDOC1 NRK CYBRD1 REG3A RGS7BP SPTSSB RP1-84O15.2 AUTS2 BACH2 AC010890.1 RPRM TECPR1 HAP1 LPHN2 SLC25A41 IVL STON1-GTF2A1L RP11-259O2.1 RNF123 RN7SL160P DEFB129 KDSR HAVCR2 RP11-830F9.5 PHLDB2 BMP5 SPATA8 APOOP5 CEBPE AGTR2 OLIG2 TRBJ2-6 CTD-2545H1.1 RP11-538I12.2 FCRL1 C2orf40 TMEM26 LRTM2 RP11-789C17.3 CITF22-24E5.1 ADARB2 SLC16A1 EVL LNX1 HGF SOX18 SYNGR1 TEX15 MYBPC2 CTC-484P3.3 CDK3 MIR891A PDE4A YPEL3 SOX2 C14orf177 CYP1B1 ESPNP CACNA1G VGLL4 SPC24 CTD-2143L24.1 KCP RP11-125O18.1 NXPH2 AP000275.65 PRKRIRP4 SCARF1 SLC6A3 APC2 SLC6A17 CYYR1-AS1 AC073130.3 CD1C EYA2 ZNF471 MAGEA4 CERS4 AVP EFS CCDC13 MCTP1 ZNF652 MEIS1-AS3 L3MBTL1 RP11-390E23.3 RPL39L FAM50A OAT RP11-475E11.9 MIR549A RP11-344P13.6 LINC00332 RP11-13J10.1 BARHL2 RYR3 MIR137HG ZFP92 TSPAN11 AC006538.4 TMEM130 LINC01435 LINC00445 LINC00643 DHCR24 MYO1B ZPLD1 PRTG LINC01547 RP11-875O11.1 RP11-346D14.1 RPL31P11 TLX3 CNTD2 C12orf42 FGL2 ZSCAN18 ADAMTS18 MICU3 DCHS2 RGMB MIR767 ELFN1-AS1 MATK RAC1 AC112719.2 REC114 CTNND2 MAST2 FRMD3 C1R SUSD5 COL22A1 OR8B2 GUCY1A3 ATP1A3 TC2N RBFOX3 UGGT1 PROZ PACSIN2 SEC23B SNORD116-19 ZFP28 PLCB2 TMEM204 KCNF1 KLF11 ZNF843 RP11-58E21.5 RP5-1029K10.4 MIR181A1HG ESX1 RP5-1024C24.1 DOCK2 BAI3 NOSIP DPYSL2 GOLM1 MAGEC3 KEL AC096772.6 MIR381HG FTHL17 RP11-384F7.2 RNVU1-8 CD1D NGF AFAP1-AS1 CTD-2012J19.3 SNORD114-29 CYLC2 AC010091.1 CTD-3138B18.4 PROM2 LMNTD1 RAB3C C6orf223 GPR101 VSTM2L ALOX5 TRIM22 CDH13 RP11-655G22.2 RAB8B MDGA2 GJA1 MIR9-3 HMHB1 FGF12-AS2 RP11-483C6.1 SLC39A7 BTNL9 LINC00574 VEPH1 GK2 FAM129A NXPH3 OR14A16 CTC-420A11.2 SMIM3 GPX7 CTD-2021J15.1 ZYG11A BASP1 ACYP2 MIR7-3HG TCAF1 SLC22A18 ARHGEF7 SLC23A2 BDNF-AS CRYM MIR382 PHACTR3 KIAA1683 EPHA2 TCHH CYP2U1 SQSTM1 RP11-662I13.3 PROK2 ZNF197 MEOX1 C15orf59-AS1 RP11-232C2.3 ZACN CMIP ZNF274 TRDN TDRD15 RP11-209K10.2 CARD11 RP11-179A16.1 CFAP46 TSSC1 RP11-269F21.2 TMEM45A C1QL3 FAM150B CDR1-AS SLC2A13 RP11-475O6.1 DAB1 KRT28 SYT2 ST3GAL1 RP11-379H8.1 RFTN1 GDF6 BTG1 MIR346 RP3-510L9.1 ZNF148 SSBP4 IKZF1 ZNF141 DAOA-AS1 HIF3A RP11-703I16.3 OR2J2 MYH16 ANKRD63 SNTG1 KCNA2 AQP10 FOLH1 RNA5SP38 SEC16B CHST1 C5orf38 SLC16A12 TAF1B LINC00898 NPAS1 AC002066.1 RP11-578F21.10 TRPV6 IRX1P1 RP11-254I22.2 RP11-21A7A.4 AC003005.2 EVC2 MIR525 FRRS1L GREB1 AFG3L2 TGFBR3L NEUROG3 TCAM1P ADIG BDNF LMBRD1 LDLRAD4 CTD-2368P22.1 CCDC26 SEMA3E CLEC2B SLC8A3 MAGI2-AS1 TSPAN8 FAM72B C3AR1 RP11-613D13.4 RP11-160H12.3 CADM2 RP13-766D20.4 TLX2 RP11-22D3.1 SNHG11 MAEL AL589787.1 SDR42E1 CPLX1 CTD-2201E18.3 LINC01057 FAM183B TMEM233 ANKAR EFHD1 AICDA RNF180 ZNF665 DCLK1 MYEF2 TLR6 AC007319.1 RP11-399K21.14 RP11-468I15.1 NDST4 ADAMTS9 ADORA2A MRVI1-AS1 TBX2 RP11-109I13.2 MIR1295A CTC-467M3.1 CELF6 COL21A1 NTNG2 TUBA3C ADCY5 RP11-504A18.1 RP5-942I16.1 HOXB3 AP000695.6 FAM135B PPAPDC3 RP11-474D1.4 ENO1-AS1 NR0B1 RP11-285E18.2 MORN1 AC003006.7 KLHDC7B ZNF716 RASGRP2 AC072061.2 TSC22D3 APOB TSC2 NRP2 TBC1D22B CDH2 RP11-744N12.3 SEPT4-AS1 FGF20 SNHG23 LAG3 AIM1L KRT3 FLNC PRAF2 RP11-67L3.4 DPP10-AS1 SPTAN1 PLCH2 MIR137 RP1-170O19.22 TENM2 HOXB13 RP11-108K3.1 TMEM72-AS1 PNMAL2 HOXC-AS1 CITED1 RP11-478C6.1 CNN3 AC021218.2 MIR105-2 CACNA1B PAPPA MYT1L-AS1 SERPINB7 RP11-79P5.9 PRRT4 CALCA ENAM DLL1 CCND2-AS1 SUN1 MAPT TRBV4-2 HLA-DPB2 TRIM55 RASGEF1C AC113607.3 PLXNC1 FOXO6 NUPR1L CTD-2113L7.1 KIAA1755 MGAT5B FAM208B SIX3 HDAC9 DUSP26 HOXA3 TOM1L2 RNASE10 APLP1 MNDA DLX4 AC006116.17 ZCCHC18 FOXJ2 CTD-2034I4.1 HHIP-AS1 PHOX2B MYT1 AC093627.12 RP11-629G13.1 FAM133CP CPB2 AC138430.4 RNF219-AS1 RP11-462L8.1 ZCCHC24 RP11-283G6.4 FGF19 LINC01465 PTP4A2P2 STAP1 GUCY1B3 NR3C1 DLX6 MIR300 GPR133 RP11-14J7.6 MAP2K6 LEFTY1 FSTL4 AJ011931.1 RP11-261B23.1 PFN2 TCEA2 PNLIP ARHGAP28 ZNF80 TMEM257 FAM181B COX8C RP11-608O21.1 MIR124-1 FAM110A INO80E KAZALD1 BNIP3 CARD6 CCDC170 CTD-3193K9.3 LINC00314 TXNRD2 FES HTR3B RP11-501J20.5 NPTX2 OR6C74 C17orf107 RORB SNAPC4 RP11-102K13.5 CDK6 CTD-2385L22.2 HMX2 CTC-254B4.1 RP11-556G22.2 RP5-877J2.1 IGHM GABRB1 RP11-351J23.2 SNAP25 CTD-2583A14.9 MUC12 FBLN1 MYOF TLL2 RP1 SUB1 ANO1 DEFB124 ZIC1 SLX1A-SULT1A3 FURIN RP11-705O24.1 ARL10 RP11-469L4.1 MAPT-AS1 LTB4R2 ST8SIA1 HLA-DPA1 PLET1 SMIM12 JAKMIP1 RP11-431K24.1 CNRIP1 NALCN CHST4 SYT15 RP11-713D19.1 SF3A2 KRT76 CTD-2544M6.1 SIRPB1 SOX3 FCER1A KRT87P GOLGA8A ADRA1B DPY19L2P4 TMEM57 LTB KCNMB1 DES RP11-367G6.3 CERS1 RP4-705O1.1 RP11-100E13.1 LMO2 PPP2CB RBM20 XKR7 RPL22 RGS7 MORN3 ASIC1 FER1L4 RP11-152K4.2 RP11-247C2.2 RSPO1 PRB2 CHAT TTBK1 RP11-60A8.1 MYCT1 S1PR4 KYNU SRGAP2 PDILT TFAP2D RP11-410D17.2 ZFP30 PEX7 IL22 MYH10 RP11-278L15.2 SPATA22 CLVS2 CYP7B1 KCNC3 OR10J1 AC005237.4 HSF4 LOR SCGB1D4 ARMC4 AL132709.5 RP11-1134I14.4 RP11-364B14.3 ASCC2 KLHL35 AC067956.1 FBXO17 GFM1 RP11-154H12.3 FAM196B RAB11FIP3 OR2I1P GPR123-AS1 PCK1 AA06 CYTH2 DMRTA1 SLPI IRAK3 MYO5A ZC3HAV1L PARP6 MRPS22 RP11-420N3.3 GNG4 AC016831.7 GAMT RP11-317M11.1 PPP2R2C P2RY12 PNPLA1 MIR218-2 NOX4 LHX8 VTN RP11-388K12.1 TBX1 PYHIN1 LINGO1 CIDEC RP11-25O3.1 SALL2 CD300LG RP11-363J20.2 LRFN2 WDFY4 AC104532.4 NRG2 OPRL1 IQCG KRT8P4 RP11-261P13.5 HTRA3 SPTBN5 RP11-469H8.6 OR4C12 STK33 RBM46 DKFZP434H168 ANKRD18DP SLCO1A2 WDR49 BMP4 AC073109.2 OSTM1 TRBJ2-3 AGTR1 SLC12A9 OTP MIR1258 POTEA PCDHGA9 RP11-672A2.3 JPH4 RP11-701I24.3 GALNT7 OBSCN SPECC1 FAM155A TRIM61 CBX8 MIR598 DPY19L2 CYYR1 FOXN4 CDHR3 TJP1 KIR3DL1 LINC00982 CLDN17 ULK4P3 ZNF599 RP11-152L20.3 LINC00689 CTD-2134P3.2 AC010967.2 SLIT2-IT1 AP000783.1 CCKBR FAM118A CCK TAF4 OR5H6 TRIM9 RP11-394A14.2 PRAP1 RP11-457M11.6 KY UTP14A WRAP73 ERICH3 CSMD1 RP1-111D6.3 AC092684.1 TRERF1 SLC24A2 RP11-21B23.2 ADSSL1 GLI3 LRRTM1 VAV2 COL25A1 RP11-23D24.2 AC073869.2 RP11-379L18.1 COX19 RP11-81K2.2 SCEL OSBPL6 SPANXN2 BCOR CCDC70 SH3TC1 AVPR2 GRIA3 FEZF1-AS1 KCNK12 BHLHB9 GOPC FOXD2-AS1 MEOX2-AS1 DEFB135 RP11-501C14.7 OR5E1P SLC35F3 OR12D2 BEX4 INHBB CUX2 RP11-328P23.3 HYDIN CTB-96E2.10 SPACA7 RAB31 LGALS14 DHRS3 RP11-426C22.5 ZFP82 CTD-2023N9.1 AC004987.9 C5orf58 CTNNA3 AP3B2 LCNL1 ZEB2-AS1 RP11-88H9.2 RP11-556I13.2 FREM2 RP11-744J10.3 TBX15 ZNF829 ARNTL RAPGEF4 RAET1E-AS1 CST5 FUZ ZNF813 TTC3P1 RP11-814P5.1 CTB-61M7.1 Six3os1_6 LINC00922 TRIM31-AS1 NIM1K FADS6 C16orf96 C16orf86 AGAP2 LAMC1 DOT1L ZNF521 PDE1A SNORD113-2 OR2T34 GPR88 MS4A6E ENKD1 KCNK4 ELOVL4 RP11-33N16.3 MGARP ONECUT3 ANGPT1 CCDC114 AGAP2-AS1 CCDC8 CASKIN1 MRPL44 HMGA2 RP13-895J2.8 AC007743.1 MIR1185-2 HHAT EMX1 TRPM4 LINC00632 AC010731.4 LTB4R FAM186A NWD2 ISYNA1 CREB5 ZFR2 CTD-2014B16.3 AP004372.1 ANGPT2 B3GAT2 THSD7B LAMP5 ACSM5 SLC4A10 BPIFA4P LHX1 C1orf95 OR2G6 SSPO DNAJC6 RP11-708B6.2 FERMT2 MIR105-1 NSUN2 BX322557.10 MAGEC2 CTD-2330K9.2 CTC-360G5.8 CPNE8 CTD-3051D23.1 DACH2 LINC00620 KIFC3 RP11-293I14.2 FAM92B SHD ARSI RP11-45A12.2 RP11-188C12.3 NUS1P2 RP11-625L16.1 GPX6 ZNF329 ZNF610 MIR320D2 RP11-434H14.1 LINC01169 AC073343.13 HLA-F RXRG NMUR1 KIR2DS4 DNAH10OS RP11-768F21.1 FAM183A SEPT7-AS1 KCNK2 KDR SORCS1 PCDHGA2 TBC1D4 AF196779.12 GIT1 DNM3 SYT1 RP13-314C10.5 RNF207 AC016738.4 CCDC84 FAM13C MOXD1 SYNDIG1 ST3GAL6-AS1 AL122127.25 AC097467.2 MKX-AS1 DPF3 ZNF701 KCNT2 RP11-44K6.2 RP11-445F12.1 CBLN1 RARA RP11-570L15.2 RNF220 PTPRS TRABD SFRP5 MIR329-2 TNC OR56A3 LINC00856 AP001331.1 DLG2 ITGB1BP1 RP11-572C21.1 RP11-5A19.5 PDE2A AC009487.5 MIR3663HG YPEL4 AGBL3 SACS WWTR1-AS1 AFF2 LAYN NCKAP5 PLXNA2 ZDHHC15 KRT27 MN1 RORA PTPRD LY6G6D LINC01305 MOSPD3 MYH15 RP11-282I1.1 SHISA6 FAP MIR892B GPR37 LINC00933 KCNJ1 SLC22A17 KIF2B DEFB107A SCARA5 AC000403.4 EBF4 LINC00327 SCGB1D1 RP11-422J15.1 LINC01141 UGT1A9 RP11-269F21.1 GS1-421I3.2 AC034228.2 ATP1A2 GPR50-AS1 FOXL2NB DGKG RP11-514O12.4 INHBA OR5B12 METTL9 TRBV4-1 LINC00200 RP11-21C4.1 METAP1D RP11-676J15.1 OR5BC1P PXDC1 ZNF663P RP11-442O18.1 PLCB1 RAI1 ATP6V0CP3 LOXL2 IGF2 ANKRD19P AC007192.6 ZNF229 AC099754.1 CTB-77H17.1 CHRNA1 RARRES2 GSX2 CLIC3 HOMER2 RP11-8L2.1 HTR1B PIWIL4 CTD-2377D24.4 ADAMTS1 HOTAIR_3 PRKG1 P2RY6 PLXDC2 GPR27 GRID2IP ELAVL4 CADM3 CDH9 SLC7A5 RP5-906A24.2 GZMA NFIB AC010729.1 AC009108.2 CTC-301O7.4 KHDC1L GUCY1A2 RP11-355I22.2 RP11-313I2.5 ZSWIM2 TBCA PACSIN1 LRCH2 GPR115 CYGB LIFR RFPL4B ANXA8L1 RP11-715H19.2 PPAPDC1A CACNG8 CHST9 RP13-379O24.3 SLC35D3 RP11-494M8.4 CTC-525D6.1 AC068499.10 RP3-331H24.5 SRSF10 TTC28 MAGI2 NLRP2 MAG FLT3 PCDH19 FOXP4-AS1 AC008060.7 DLK2 UCP1 SEMA6C CNN2P8 ARHGAP19-SLIT1 NOTO SRPK2 PLCL1 GRIN2D OR8H3 ECEL1P1 HPCAL1 VGLL2 GALNTL6 C11orf87 CYS1 KIAA1671 RP11-70C1.1 ANKFY1 RP11-654C22.2 SNHG24 RP11-661C8.3 CTB-33G10.6 CHRNA4 HCG4 CLEC2L RP11-672L10.2 LINC01519 RP1-170O19.23 4-Mar RP11-325B23.2 WNT7A SLC18A2 ASIC2 TSHZ3 MIR4787 FIP1L1 IQSEC3P1 RHOB GNPNAT1 GNAQ DOCK10 IGDCC4 HOOK2 PRB1 RP11-359E10.1 AATK-AS1 HCG4P11 LINC00461 AMH MTMR7 NEU4 COL2A1 PTH2R FNDC3A MIR1180 LINC01096 METRNL RN7SL479P RASSF2 RP11-105N13.4 SLC30A10 DLX3 NECAB1 AVPR1A NPBWR1 FOXG1-AS1 GRM5-AS1 CTC-512J12.6 COL14A1 PRKD1 SOX8 PDX1-AS1 RP11-310N16.1 LRRC4B CALCRL SCGB2A2 ATXN8OS CTD-2168K21.2 POT1-AS1 EGFL6 BRSK2 PCDHGB5 TBX5-AS1 LHX6 MIR495 MAGEA8-AS1 PNMAL1 PRR18 PCDH10 MIR892A CELSR1 CYP1B1-AS1 PRR5L OR52B6 TNNI3K IGLON5 THBS2 NEXN SRPRB FOXF1 AC079776.3 LINC00271 TPO RP11-267N12.1 AC007193.6 ETS1 SIX3-AS1 MIR34C TTYH2 GATA5 HSP90B1 MRGPRX3 RP11-506B6.6 SMOC1 HOXB13-AS1_1 HOXB6 TMEM131 RP11-382A20.4 PDHA2 MFSD4 STEAP2 TRAV35 ZNF528-AS1 CTB-96E2.3 RP11-148K1.1 OR5A1 ZP4 OR8K5 RNASE11 ST6GAL2 RP11-286E11.1 OR8D2 SOX21-AS1 BICC1 RP11-370P15.2 AC244205.1 SLC45A4 ADAP1 HOXA4 SLX4IP PTS KCNK13 SEMA3D EID3 SLFN13 CTD-2619J13.5 CCDC169 RP11-175E9.1 RP11-521O16.1 MMP17 TAS2R40 ZNF569 MROH9 CHIA LINC01475 ASB18 LYPD5 TBX20 FAT3 ZNF273 DST C18orf42 CAB39 RP11-160H12.2 MSH2 CTD-2555A7.3 NPPC ZEB2 RP11-58C22.1 WSCD2 VLDLR-AS1 SERTM1 NEUROD4 C6orf165 RP11-638L3.1 MGAT4A EIF4E3 TSC22D4 CHRM2 RP11-342A23.1 DKK1 CERS3-AS1 KCNH5 RASL10A 1-Mar OR5K2 GABRA4 CTD-2026G6.3 CTD-2562J15.6 GS1-259H13.10 PDE11A MIR124-2HG COL4A2-AS1 CADM1 NOTCH4 RP11-465L10.10 INPP5D ARHGEF1 ABCA4 TSPAN9 OPRM1 RP11-13P5.2 CLYBL C6orf136 CYP26B1 APCDD1 VN1R1 LECT2 RAB6C SPATS1 RPL23 RP11-810P8.1 NKAPL AQP5 TMEM163 MS4A7 MPDZ PRKAR1B EYS CCDC61 FAM189A1 RP11-849I19.1 IQCJ RNASEH2A DEPTOR MOGAT1 CCR10 ABHD15-AS1 ZNF470 RP11-493L12.3 MAP1B SLC13A5 HLA-L VCAM1 C11orf63 PPP2R2D RP11-122D10.1 TGIF2LX EDARADD MIR548F2 ROBO2 LINC00152 PCDHB3 TRGV11 CR2 BEND6 RP11-158H5.8 CTD-2118P12.1 RSAD2 1-Dec RP11-6D1.3 CTBP2 ZMYND11 KRTDAP CDH23 TTN CTC-573M9.1 SLC26A5 RP11-554D15.1 RGS6 SRGAP3 GUCY2EP RP11-613M10.9 RNF144A-AS1 MIR6128 AKAP13 USHBP1 ZFYVE21 FLG IFNA1 RNF121 RP11-557H15.3 HHIP FAM196A ADH1B AC087651.1 HOXB13-AS1_2 SARDH LINC00678 WNT5A-AS1 RP11-174G17.3 CD46P1 CHL1-AS2 CCDC146 HSPA1B RP11-444D3.1 SLC39A11 ATP12A BTBD7 SPRR3 AC104655.3 S1PR5 PITPNM3 PPP1R1C NOLC1 AC002076.10 ZNF667-AS1 SNORD113-4 UNC13C IDUA RHOBTB3 HMX3 RP11-227H15.4 PRR35 OR5V1 OR1J4 RIMS2 NPTX1 NPTN NPY2R PKDCC RP11-128A17.1 NOP14-AS1 RP11-1084I9.1 FOXP1 CLEC4G INO80B-WBP1 LINC01056 OR7G3 RP13-238F13.3 EPHB6 C16orf95 C6orf25 HIST2H2BA TRBJ2-4 RNA5SP107 KLHL17 TMEM145 RGMA TJP2 FAM159B AC004051.2 MUC21 RAB27A PAX3 AC005606.14 LINC01510 RP11-437J19.1 DEFB122 CACNA1G-AS1 RP11-120A1.1 KSR2 PPP1R3C ST8SIA2 LMX1B RP11-278H7.1 HLA-DQB2 NOVA1-AS1 GS1-279B7.2 DLGAP2 C4BPB LPPR3 MICALL2 CHRNA9 SPATA8-AS1 SOX11 HIVEP3 SLC16A8 SPATA13 TCEA3 PPBPP2 TOPAZ1 ZNF736 BMPR1B RP1-209A6.1 LINC01297 TLL1 ADAMTS10 CST2 BRF2 RP11-1070N10.4 NLGN4Y RAMP2 F11R RFX4 RP11-122C21.1 PDZD4 RP11-147K16.3 TM4SF19-TCTEX1D2 LINC01234 RP11-481J13.1 DAW1 REM1 RP11-434C1.4 LINC01482 FZD1 TRPC4AP AGAP1 GPT2 ANKRD1 LAMA4 PMEPA1 NPS RP11-525A16.4 MIR133A2 RP11-89K21.1 CTD-2540M10.1 SYNPO CCDC37-AS1 MTCL1P1 HOXC6 GMNC CTNNA2 AC016723.4 HLX-AS1 CCDC92 MIR4281 AL049564.1 ASCL1 LKAAEAR1 PEX5L PVT1 NMD3 SLC2A9 PRAMEF12 UCHL1-AS1 DNER PHOSPHO1 MAGEB1 AC135050.1 AC104820.2 CD300E HPVC1 SALL3 LCE2A EMILIN3 RN7SL519P FMNL2 XIRP2 AC007405.8 OR8B3 ANXA4 AC004699.1 RP11-394O2.3 SERPINI2 PARD3 AC087499.5 RP11-449J10.1 AC073072.5 AP001347.6 ONECUT2 FAM110B NRG1 DCAF12L1 SYT9 TGFBI OR2L3 CTD-2298J14.2 HAO2-IT1 AC009313.1 LINC00691 RP3-462D8.2 AC005537.2 OR5T1 LINC01104 RP11-150O12.1 TRDC CTC-398G3.6 DLEU1 JPH3 RP11-304L19.12 OGDHL SLC22A10 SPINT2 AC006547.13 RP4-569M23.5 AC009404.2 RP11-546B8.6 LRRC37A6P SLCO4C1 CTD-2576F9.2 FAM155B LHX9 BAX MCIDAS DUSP9 IGHE RPL37 NT5C1A NANS CNPY1 GALNT16 SPG20-AS1 RFTN2 NPAS3 SLC35F4 LCE4A TRIB2 COL6A6 AHSP DNAH5 KHDC1 MAD2L2 ZNF300P1 PTPRD-AS2 MAL2 LINC00410 TUBB4A XXbac-B562F10.12 STX4 TRPC3 UBE2DNL KLRD1 AC145676.2 FMN2 TRIM8 RNF6 SEMA3C BOLA2B SCN5A AC011754.1 GRAMD3 FBXO10 EVX2 CACNA1I PDGFC RP11-565A3.2 LINC00242 VHL ZPBP KIAA0825 FAM49B C1orf68 RP11-867G2.8 LINC01359 BCKDHB TRBV6-1 GALNTL5 NPHP3-AS1 PAH IGHD2-8 IFNA8 RP11-145O15.3 AC018890.6 CIART ASB2 SNCA-AS1 FRMD4B RPRML RP11-662G23.1 CFAP45 RP11-342D14.1 GOLGA8B OSBPL5 MMP9 DLG3-AS1 CRCT1 RP11-193J6.1 RP5-963E22.4 |
| GSE Hypermthylated Genes | 50 | RIC3 C6orf32 HNT FLJ10781 FLJ14054 C6orf155 C20orf39 LOC349136 BPIL3 OR1G1 RLN3R1 CUTL2 FOXG1B ACTN2 ASGR2 DEPDC2 ZNF542 FLJ30834 ZNF312 GPR109A FLJ46831 CESK1 SKIP RSNL2 PNOC EFHA2 CARD14 VGCNL1 CNGA2 IGF2AS HABP2 HTR3D WIT-1 LAD1 FCGR3B FLJ25477 SAA1 LRRN6C BAPX1 KRTAP15-1 WDR8 LBP C20orf71 EFCAB3 C20orf185 BPIL1 OR1A2 C2orf32 TSCOT TEX101 |
