# Supplementary material for: Consequences of aberrated DNA methylation in Colon Adenocarcinoma: a bioinformatic-based multi-approach
Source: BMC Genom Data. 2022 Nov 29;23:83. doi: 10.1186/s12863-022-01100-7 (PMC9706923; doi:10.1186/s12863-022-01100-7)
Supplement: Supplementary file 3 — Additional file 3: Supplement 3. Investigation of Hypermethylated and Downregulated Genes. [file 12863_2022_1100_MOESM3_ESM.docx]

**Supplement 3- Investigation of Hypermethylated and Downregulated Genes.** Identification of hypermethylated and downregulated genes in CRC by Venn diagram. Forthy-four hypermethylated and downregulated genes were identified for further investigation.

| **Names** | **total** | **elements** |
| --- | --- | --- |
| GEPIA2 Downregulated Genes  Hypermethylated Genes | 44 | HPSE2 SDC2 SPG20 RSPO2 ZNF667 SFRP2 CHST10 HAND2 NPY ZNF677 FIGN GPM6A AMPH D4S234E ADHFE1 CNTN1 TRPC6 GRIK3 NRXN3 GFRA1 FLT4 JAM3 UCHL1 ATP8B2 MAL CNR1 THBD PHOX2A EDNRB KIF5A NPR3 SOX17 NTRK3 VIPR2 CD34 GRASP CDO1 INA JAM2 RYR2 GAS7 PDE8B SFRP1 PRSS1 |
| Hypermethylated Genes | 158 | PAX7 PTPRR HS3ST3A1 LRFN5 POU3F1 KCNA1 GNAS FOXE1 RASGRF1 SLC6A15 DEFB119 CDH8 KCNQ5 ADRA1A IL5RA ATP10A FOXL1 COL23A1 ATP2B2 HTR1E DPYSL4 SPAG6 GAD2 ESR1 FLI1 TRPC4 TRHDE LAMA1 EOMES SCARF2 TMEFF2 CCDC37 MDFI BNC1 AJAP1 SCTR LECT1 CD1B GRM3 SIX6 SLC5A7 IRX4 POSTN C1orf158 NCAM2 UNC5C ZNF132 SORCS3 HBE1 PVALB ELOVL2 CALCR MMP26 GLRX PCDH17 COL4A1 ALDH1A3 DAK UMOD HS3ST2 DOK5 GABBR2 GRIA4 TREM1 BPI TWIST1 PIPOX GPR75 HECW1 OR12D3 ST8SIA5 SLC6A2 DMRT1 CCNA1 PRDM14 S100P MSC POU4F2 FAM43B ST6GALNAC5 SOX21 NOS1 FBN2 FBLIM1 SPRR2D ALK COL15A1 TRIM31 ATP4A KRTAP11-1 FZD2 ZIM2 GCM2 LRRC4 FCRL3 ZNF625 TRH ITGA8 DTX1 CRHR2 NR2E1 FCN2 KCNB2 WT1 STAC2 CDH18 VEGFC PCDHGC4 KCNK17 SARM1 CRISPLD1 CIDEA CNTNAP4 PTPRT ADCY8 BTG4 FBN1 GABRA6 KHDRBS2 GPR26 ITGA4 IRF4 PDPN CNNM1 PCDH11Y GRIK1 DGKI LCE3D BOLL NELL1 CACNA1A FAIM2 MSX1 DLX5 ALX4 PCDH8 SLC36A2 CBLN4 VSX1 KRTAP8-1 SLITRK1 MEST ACSL6 EYA4 DCC HKDC1 GPC6 BCAT1 CDKN2A TM4SF19 TFPI2 ADAMTS5 SLC4A11 GHSR LRRC3B GRIA2 GATA4 CSF2 |
| GEPIA2 Downregulated Genes | 2631 | PNCK C5orf42 FSTL1 CLMP PNMA1 ABCA3 RP11-147L13.12 PCDHB15 PLCE1 SLMAP SAMD4A CIPC KCNMA1 PINK1 SMARCD3 PKNOX2 PYROXD2 FIBIN ITGA5 CNTNAP1 KLHL13 UBXN10-AS1 AC083843.4 RP5-1039K5.12 WASF1 HSPB1 RP13-104F24.2 CLK4 AHDC1 KCNG1 CADM4 OTOP2 CSPG4 IGF1 FAM214A RP11-6O2.3 LDB3 AHSA2 BOLA3-AS1 NBPF12 PELI2 PPP1R14A NAP1L2 GNAL TLN1 MITF NTSR1 WISP2 RTN1 CPXM2 KIAA0895L UTY HOXD11 MRGPRF-AS1 CACNA2D1 COL4A5 RGN ZEB1 ZG16 CTD-3220F14.1 FAT4 IZUMO4 KCNK3 EPN2 AATK MSRB3 PDE1C LINC00665 FGFR1 IGLV5-52 FXYD6 PDLIM2 PHYHIP ZNF208 MAP3K3 NINL TMEM110-MUSTN1 CAPS AP006621.5 LRRN2 EVC ZNF582-AS1 SYNGAP1 HERC2P2 FRY SERPINF1 BZRAP1 RP11-181G12.2 B4GALNT1 RP11-350N15.6 SLC12A4 BMP3 SLC26A10 RP11-867G23.10 LIMS2 BAHCC1 PRKCB ENTPD3 CTD-2036P10.6 RP11-355O1.11 A2M BVES-AS1 CDK20 RP11-644F5.10 GLT8D2 CDK11A RP11-617F23.1 CNN1 CTD-2545G14.4 KRT1 PALLD SPACA6P MT-ATP6 AC005944.2 MT1JP CTD-2020K17.1 TMEM240 CCDC106 GRK5 SLC8A1 AP1S2 RP11-347C12.1 USP32P2 CA11 SIX5 VGLL3 ZNF83 C15orf52 ARHGEF26-AS1 SLC1A7 RP11-148K1.12 ANO5 ANKRD6 NEIL1 LTBP4 C16orf45 GFPT2 GHR PYY MKNK1 DYSF MT-ND4L SLFN12 PLEKHO1 ZNF345 CNGA3 MIB2 PODN IL17RC ITGB1BP2 ROR2 RP11-574K11.5 MAOA ETNK2 DSTN VAT1L RP11-347I19.7 HECTD2 KLF3-AS1 TNNT3 GYPC FIGF C3 HDAC7 SLCO2A1 MAPK12 CHST15 C1orf216 XXbac-B461K10.4 RASA4B GDF10 RNF217 PGA4 RP11-34P13.16 CSNK2B-LY6G5B-1181 GAS2L1 RP11-588K22.2 ZNF25 ATOH8 PIRT BCHE AC005154.7 ENOSF1 SERPING1 MEIS2 STARD13 BCL2 SRSF11 PCDH7 ZNF75A RP11-517I3.2 NPTN-IT1 RP11-157P1.4 FAM198A MAFK CHFR NAALADL1 EMILIN1 PXDN TBC1D17 TNXB C1QL1 FAXC RP11-87H9.4 A4GALT PDZRN3 RP11-755F10.3 HOXD-AS2 BCAM C14orf132 TF RP11-63M22.2 PTRF THRB NPR1 SLC9A9 CTC-529I10.1 AC009501.4 WDR19 RP4-669L17.8 ZNF331 RP11-834C11.7 POGZ NBPF19 EFHC1 CDC42EP3 UPK3BL ZZEF1 TSPAN7 RP11-66N24.3 ZNF334 NBPF8 MRGPRF CELA3A IGSF11 PENK SMAD9 RASIP1 RGL1 SENP7 MT3 DPYD AC024560.3 LRRK2 ACTC1 CROCCP3 CPEB1 MAPK10 CNTFR RP1-283E3.4 CACNB1 QKI NCAM1 ZNF549 ZNF467 ZSWIM8-AS1 PI4KAP1 LGI4 NACAD POU6F1 BBS1 MFAP5 FENDRR IL6R WDR6 TP73-AS1 CLEC3B AC132217.4 MTND2P28 EPHA4 TTLL11 LINC01579 ZNF655 RARB GDNF-AS1 TARID HLF ENHO PJA2 SLAIN1 TCF4 C15orf59 GNB4 GALNT18 NKTR PHF1 TBX2-AS1 DNHD1 LONRF2 RP11-574K11.24 OR2A9P MT-ND2 CPAMD8 STON1 AHNAK RP3-402G11.27 FHL1 PBX1 TNFSF12 CTB-31O20.4 FN1 TTTY14 GOLGA8N CBX6 CH17-472G23.4 PPP1R12A RBPMS RBM38 C16orf62 RP11-616M22.12 RP11-43N16.4 RBM6 MTSS1L SORBS2 PLXNB3 KLF4 ACAD11 RP11-11N7.5 RP11-569A11.1 FAM229A HP SCHIP1 GNAO1 BTK RP11-631N16.4 MMP23B CLCA1 IDS OLFML2A GALR2 PRPF39 SEMA3F-AS1 GABBR1 NIPAL4 TMEM25 BMP6 HMGN2P15 TIAF1 NPPA RIC3 PLSCR4 RRAD VIT ZDHHC11B RASSF4 PLA2G6 CCM2L A2M-AS1 ZFPM2 CRABP1 TDRD10 PIEZO2 SLC35F1 SYTL4 VSTM4 CEBPD LDLRAD2 AC074289.1 RDX PACSIN3 OMD ZNF568 CSRP1 AC007566.10 SVIL AFG3L1P ZFP36 CMYA5 NDRG2 EHBP1L1 SVEP1 RILPL1 PHYKPL DIXDC1 ADAMTS9-AS1 PCBP3 AC018766.4 SEMA3G ZNF354C NGFR XXbac-BPGBPG55C20.2 TCF21 MEIS1 LINC01125 JPH2 WDR91 WNT6 TMEM179 GNRH1 CMA1 CACNA1H FYN CTC-429P9.3 FST RP3-402G11.28 BEX2 KCND3 ARHGEF26 TBC1D3B AQP8 PREX2 KLHL21 CFAP44 BVES CCL14 PI15 COQ10A KLF2 TUBA1A TNFAIP8L3 SELP RAB9B NAIP RP11-1319K7.1 SOX10 FGF14-AS2 PELI3 RP11-632K20.7 DNAJB4 BRSK1 NAB2 AC093110.3 KIT DCLK2 HSPA2 SH2B1 ACAP3 EPHA7 CNTNAP3B TACR2 SCN4B PPOX MST1P2 ZNF439 TARSL2 AC009120.6 USP9Y KANK3 ACADVL PRRT1 AC156455.1 PLA2G4C ANKDD1A ICA1L PLAC9 SVIL-AS1 ENO2 RP5-855D21.1 C1QTNF7 RP11-798M19.6 C5orf56 ADAMTSL4 SLC25A34 GLIPR2 FAM49A NEGR1 EHD2 MAP1A SSC5D NEFL SNRPGP10 AC002398.12 RP11-894P9.1 AGER PARD6G HOXA5 CBS HPGDS EME2 NAV2 WNT9A ADH1C INPP5B NPIPB5 SNTA1 PRPS1P2 ZNF781 PRDM6 RNF150 CLPS INCA1 SLC16A2 GK5 RUNX1T1 SIGLEC1 ADD1 AGAP4 AL928768.3 RP11-681H18.2 SMCHD1 RRAS PPP1R32 S100B NPY1R TOX MGP TAC1 TRPC1 GCNT2 RP11-69E11.4 PLEKHO2 CTC-524C5.2 KCNN3 SST FKBP5 NPIPP1 MAPK7 SRGAP2C RP3-525N10.2 RP11-419C5.2 CCDC176 CALB2 TMOD2 MAB21L2 SCNN1B DENND2A ECHDC3 PTPN14 KCNAB1 ZBTB47 DMTF1 PDE4D ZNF542P SNCA TSPYL5 LRIG1 RP11-574K11.31 PRCD GAP43 LINC-PINT RBP7 LINC00641 C20orf166-AS1 GNG2 HCG27 NR2F2-AS1 PDE3A HOTAIRM1 RP11-203M5.7 ZNF536 FOLR2 CAND2 GOLGA6L5P TRANK1 CPB1 EPB41L3 WSB1 BCL6 MAP3K6 NPIPA5 MAP6 RP4-791M13.3 CTB-109A12.1 RP11-373D23.3 ENPP2 SETBP1 PITPNM2 AIF1L FHL5 SLC6A16 RP11-792A8.4 DOCK4 GOLGA6L9 SHANK3 MAN2C1 FAM127A RP11-152H18.3 ADIRF-AS1 TBC1D3L CTD-2033D15.2 NRXN1 SGSM3 SCART1 FRMD4A C3orf70 ASIC3 YJEFN3 RPS4Y1 TMEM259 UNKL RP11-33E12.2 SOX15 RNPC3 ARHGAP10 PIANP GP2 WDR86-AS1 KLF9 MIA PCSK2 TSPAN2 6-Sep CEMP1 MRC1 MT-ND6 BRICD5 TMEM178A CTD-2270P14.1 MORN5 RP11-848G14.5 PPP1R3F PIP5K1C ZDBF2 CTSF ZBTB4 FAM228B GPT KIF5C PDK4 TRIP10 GFRA2 NPHP3 ADCYAP1R1 FILIP1 ARGLU1 MAGI2-AS3 ZNF90 BEND5 IGFBP5 NRTN AC005682.5 RP11-379B18.5 SSBP2 RERGL COLEC12 LIX1L IRF2BPL GUCA2A NPIPA3 REEP1 PIFO MYOCD RP11-254F7.2 DPP6 LSAMP SMARCA1 CD109 ANGPTL1 RGS1 FHOD3 UBE2QL1 RP11-553L6.5 RP11-793H13.8 LINC01088 GADD45B FGD2 ZNF862 KLHDC1 DAAM2 EFEMP1 LINC01140 P2RY14 PCSK1N AC005104.3 MOCS1 GBA2 SLC25A42 SYP GALNT15 SYN2 KIAA1109 EEF1A2 RASSF8-AS1 ANKRD65 ZDHHC17 ITGA10 ABCC9 RP11-390P24.1 HACD1 RP11-345P4.9 GPR155 RP11-89K11.1 PNISR ACSS3 SPEG COX7CP1 GREM2 HERC1 ZNF577 C4B SHC2 CASQ1 INPPL1 CACNA1C CHGB IGHMBP2 FAM73B IFFO1 RP11-490M8.1 CCDC107 MDGA1 ZNF767P ADARB1 5-Sep CCL11 RP1-286D6.5 COL5A3 COL4A2 ALDH1L1 CCL23 OBSL1 C1S CDRT4 TCF7L1 CITED2 EGFL8 GPIHBP1 STARD8 FAM126A FOXP2 RP11-334J6.7 PKIG NLGN4X CSDC2 ZNF528 RP4-717I23.3 EFNA5 RP11-394O4.5 NXN RP11-263K19.4 MFSD7 BGLAP RP11-274B21.2 HTR4 MRAS RP11-250B2.3 PFN1P6 AXL MT-RNR2 NRN1 KIAA0408 SHE F13A1 PALM SASH1 CTD-2562J17.6 KCNQ4 OGN PHC1 DUSP3 TUBB2B HHEX CEND1 FLCN MPZ TCEAL2 PCDHGB7 ABCA10 ZNF493 HDAC4 ZNF582 AP000347.4 GFOD1 GDNF LINC00595 STK36 WASH7P FOXN3 NTRK2 DSTNP1 MFGE8 GSTM1 KIAA0513 KIAA1644 CA4 RP11-348N5.7 HSD17B3 APBB3 HOXA2 RP11-374M1.5 CLIC4 GPSM1 HIC1 RP11-798K23.5 CH17-258A22.4 AASS RP11-434H6.7 ZNF790-AS1 NKX2-3 TNRC6A GNG3 ZNF793 SLC8A2 TACC1 C10orf128 SAMHD1 DMXL2 PSD GNAZ LILRB3 CTB-13F3.1 SCG2 MYSM1 TCEAL7 RP5-1074L1.4 CDON TRIM73 MS4A4A PKD1P1 C8orf46 EID2B CD99L2 HOXB4 SPOP BLVRA ABCA6 NBEA GNAI1 DNM3OS RASL12 SLC25A4 MYL3 ZMAT1 THBS4 ENOX1 MYLK PRIMA1 HOXD10 ZFP36L1 RP3-368A4.6 ARL4D SH3BP5 DZIP1 RNF146 AKAP6 SNX32 ZNF10 TRIM52 HOXB-AS1 LYVE1 IGFBP6 GTF2IRD2B ADAMTS8 LCN10 RP11-983P16.4 PRICKLE4 RP3-461P17.10 PLCD1 AGAP6 RP11-394B2.1 STAB1 DDX3Y MXRA7 C9orf172 CFL2 PDLIM4 NAP1L5 LAT IQCJ-SCHIP1 TMIGD1 P3H2 HERC2P3 MCOLN3 RNF152 TPSB2 CHRDL2 TAGLN3 MASP2 RP11-566K19.6 ZNF211 NISCH BBS4 MIR99AHG TENM3 PALMD RGS11 HOTAIRM1_2 ITPKB AKAP12 MEOX2 SLC25A27 STAT5B XIST TMEM91 CEP85L SLC39A13 SHISA4 TUSC3 HOTAIRM1_5 FBXL22 ARHGEF4 HSPA12B RP11-500M8.7 FEZ1 DIRC3 APOD ZNF880 PDE5A FBLN2 SCARA3 NCKAP1L EMP3 CPA3 CBX7 RP11-110I1.5 OSBPL1A MYOC SCN1B AC003090.1 RTEL1-TNFRSF6B PID1 TAL1 PPP1R3E VAMP5 ARMCX1 ABCC5 ADORA3 TCAP HCFC2 ABL1 RP11-411B6.6 CRIPAK RAI2 ANKRD44 AC016757.3 PWAR6 VIP CRIP2 AKR1B1 WASH5P FAM184A GPER1 RP11-334C17.5 ABHD8 MAP3K12 CCDC136 SEMA6D LMBR1L HERC2P9 SYNE1 RBM5 RHOT2 FAM110D ERO1LB UVSSA RGL2 CRELD1 SNCG LINC01082 WDR27 CTB-89H12.4 AC137934.1 CTD-2192J16.22 SYT11 ZNF415 PKHD1L1 ST5 LRMP HTRA1 ZNF853 LHFP ATP2B4 CTD-2026K11.1 TNFRSF14 GUSBP11 LINC01089 RP11-834C11.4 RAB34 ROBO3 FAM66C RP11-471B22.2 MZF1 ZFP2 MT-ND5 FAM50B PTPRN P2RX1 COL16A1 CACNB2 RHOQ CTC-529I10.2 RP11-235E17.6 TMEM200B HBB GNG11 ZNF606 METTL24 RHOJ GOLGA6L4 RBFOX2 CRMP1 GNG7 PGR PLIN5 MT-CO1 PRPH SRPX FBXL7 FGF10 RP6-159A1.4 KRBOX1 CLIP4 LUC7L3 MAPK11 ABCA9 CAMTA2 DDX17 SMTN CCDC180 ENO3 ZNF204P L3HYPDH CMTM5 TWIST2 SCN7A GAS1 ZNF793-AS1 GJC2 RP11-81A1.6 PEG3 TSPAN18 AKAP8L GPR17 RP11-356J5.12 ZNF532 AOC4P LINC00893 HAND2-AS1 IL10RA GAB3 RP13-39P12.3 NPIPB4 RP11-61I13.3 NEURL4 KIF1A SLFN11 SYDE1 SGK494 IL4R KANK2 RP11-305K5.1 CCDC69 HSPA7 EIF1AY ATP6V1G2 PLGLB1 OR51E2 RP11-266L9.8 SMIM5 SPAG8 LY6G5B MAMDC4 DZIP1L FAM124A CLIP3 LOXL4 ZCCHC11 FGF13 NLRP1 HOXA-AS2 UBE2E2 LAMB2 MYL9 TGFBR3 PBXIP1 ZNF512B C6orf49 LRRN4CL RP11-147I3.1 AKR1C1 NPIPA2 SNHG22 LPL KIZ PTOV1-AS2 CLEC2D ANKRD53 RP11-166B2.1 CEACAM19 PTPRM SSPN TMEM59L RP11-734K23.9 IGSF9B PHKG1 CHADL MADCAM1 CHRNA3 RP11-161M6.2 TPM2 RP13-516M14.10 FAM219B KLHL42 TCEAL3 OLFML3 NDRG4 GJC1 GATA2 RP11-677O4.6 C20orf194 RNA5SP216 HRC RP11-346C20.4 ZNF37BP FAXDC2 PIK3R1 GPC3 ACACB SV2A MAF TAF1C RASSF8 PDGFRA ADCY4 RP11-424G14.1 DMD ADM C22orf39 RP11-927P21.5 ADCY2 IL17B MS4A12 TTC14 SH3BP5-AS1 MAPK8IP3 DLC1 KLHL5 PPP1R1A SELM NOVA1 ELMSAN1 CTD-2537I9.12 PNPLA7 CDH19 ZNF135 CCBE1 BOC PER1 PGM5 MUSTN1 ARHGDIG RND2 LRRC75B FXYD1 STX1B RP11-244O19.1 AF131215.9 MASP1 RUFY3 ADAMTS9-AS2 SH3BGR PCDHGC3 LDB1 CTD-2054N24.2 ASB5 ZNF671 CAV2 SMG1P7 LINC01341 RP11-545I5.3 RP11-379F4.4 NEK9 ACTA1 CTC-429P9.2 NEURL1B MAPKBP1 CPE DCHS1 MBNL1-AS1 RP11-147L13.13 WIPF1 TPPP INPP4A BMX RP11-164J13.1 UST GSTM5 CALD1 LCAT NHSL2 ARHGEF25 POMC ZNF418 ZNF404 SLC24A3 KIAA1462 TGFB1I1 MT1A PYGO1 C11orf96 C1orf228 SOBP ST3GAL5 ITGA7 COL24A1 CPT1C RP11-736K20.4 RSPO3 CCDC159 KCNIP2 CTC-510F12.2 NAV2-AS1 GOLGA8R TSPAN4 RP11-384K6.2 RN7SKP70 ARHGAP24 AC079922.3 DCN SCMH1 HMCN2 JMJD7-PLA2G4B LTK FOXF2 PRELP GYG1 ACTN1 USP32P1 N4BP2L1 TIMP4 SLIT2 BAALC PDE1B C2orf88 HACD4 HSPB7 MLLT11 PLP1 PMP22 CCDC152 RP11-341G23.4 WFDC1 PTCHD1 RP5-965G21.6 FZD4 PRPF40B CIDEB ABCA5 FAM20C ABCC8 SPTBN4 CLEC10A BNC2 BEST4 SLC45A1 SERPINA3 DTX3 KRBA1 TMTC1 AC006128.2 GSTM2 ARMCX4 STMN2 STAB2 METTL17 LDOC1 ADAM11 PCOLCE2 CYBRD1 PEAR1 RP11-540B6.6 MT2A C9orf3 ROR1 MIR143HG TMEM88 ZFY PPP2R3A SYNM RPRM RP4-613B23.1 HSD17B14 PPARGC1B STON1-GTF2A1L CTRB1 CENPT SLC25A23 MAPK4 FAM13B GLP2R PHLDB2 CKM SLC4A4 RP11-548H3.1 GS1-124K5.11 ADAMTSL1 CLASRP FAM101B CALCOCO1 MID2 MAP7D1 MTFR1L SRRM2 C2orf40 AOX1 CYR61 RP11-309L24.6 DTNA SERPINA5 PBX2 AKT3 IL1R1 LTBP3 ABTB1 KCNJ8 HSD17B6 ITIH4 RUFY2 ADARB2 EVL SOX18 RP11-7F17.8 DIO3OS GULP1 RP11-693N9.2 SYNGR1 CFD KCNS3 REEP2 CDK3 C1orf162 HSPA12A LINC00883 HIST2H2BE SATB1 YPEL3 SOX2 KAT2B CYP1B1 CYP4B1 RP11-658F2.8 MEDAG CHST3 HNRNPU-AS1 BTAF1 CTD-2033D15.3 VEGFB RP11-197N18.2 AS3MT COLQ HMGN1P3 RBMS1 SCARF1 RP11-774O3.3 NRXN2 ACKR3 RPL23AP1 AC073130.3 FNBP1 EYA2 MAPK8IP1 RFX2 ZNF471 S1PR3 ADGRB1 GNB3 CERS4 EFS CCDC13 PDE3B MEF2C ZNF304 RP11-286H15.1 L3MBTL1 CBFA2T3 CDIP1 RPL39L PSMA3-AS1 LZTS2 CCL13 AFF3 AGPAT9 DPH1 PTGER3 RP11-410L14.2 CTD-3157E16.2 RYR3 VAMP2 TSPAN11 RP3-508I15.9 PTN LPP TMEM130 RP11-264B17.3 RP11-158I9.5 MEF2D GNG8 FAM65A AC017104.6 RP11-875O11.1 HSPB6 C5orf45 FGL2 ZSCAN18 MACF1 MICU3 WWC2 MPPED2 RP11-1334A24.5 MDFIC CHRDL1 MEF2C-AS1 EPB41L4A MOB2 SH2D6 HSPB8 LAMA2 RP11-1055B8.4 FRMD3 C1R DOC2GP MAATS1 RAP1A LZTR1 GUCY1A3 NID1 ALOX12-AS1 ROGDI RBFOX3 KIF7 CTC-359D24.3 CILP ITIH5 PRKCDBP ZFP28 FAM127C CAPN3 TMEM204 POLI KCNAB3 AC093495.4 CTD-3157E16.1 RP11-281O15.7 FHL3 PHLDA3 MMRN2 RP11-1000B6.3 ZNF248 SYNPO2 KCTD15 NUDT10 NGF ALPK3 GIMAP8 FAM95B1 TRIM22 PLIN4 GPR162 PKD1 RP3-425C14.4 RP11-958N24.1 BTNL9 CAMK1 GPNMB FAM129A MYOM1 KCNIP3 AGAP11 CD33 ZNF219 PCBP1-AS1 NXPH3 CROCC ZSCAN30 CRYZL1 CRY2 PTPRZ1 FCGR2B DIP2C RP11-136C24.3 NFATC4 BCO2 PDLIM7 CA14 RP11-16E12.1 STX2 FAM46B PKD2 CLDN11 FAM13A-AS1 TIMP3 RP11-274B21.3 RP11-1069G10.1 KIAA1683 MXRA8 CLCN6 NFATC1 PLN MAN2A2 MEOX1 FBXO27 INTU EML1 MRVI1 SHISA3 PTGDR FAM150B CTD-3064M3.3 RERG KRT13 RP1-59D14.5 KATNAL1 PAQR6 HIF3A RP11-455F5.6 MEIS3P1 FYCO1 ANKRD35 CNTN4 CTIF CTD-2303H24.2 SOCS2-AS1 DFNB59 FLYWCH1 LINC00173 RP11-680F20.12 LGALS1 GPR173 DACT3 PIAS3 VAMP1 RBM24 EVC2 FLT3LG ZNF154 GOLGA2P5 LINC00969 SYT5 MARK1 ZNF596 UBA7 N4BP2L2-IT2 OSMR RP11-732A19.5 PNMT CYP21A2 CTD-2368P22.1 HSPB3 TRIM3 ABCA1 CSF1 CLEC2B THBS3 CRYAB RP11-2N1.2 PPAP2A CKB RASD2 ULK1 INSC SYNC LINC00294 PARVA TEAD3 RP11-890B15.3 MAP3K14 RP11-333E1.2 RP11-802O23.3 CADM2 CD36 AC002116.8 CLK2 MTND5P1 ZNF692 SEC31B EFHD1 RNF180 COPZ2 HSPB2-C11orf52 RP11-753H16.3 MYEF2 DCLK1 MEF2A NEFM NNAT ANGPTL7 CDKN2B-AS1 KCNH2 FOXD3 TBX2 APBB1 PCP4L1 C1orf123 CHD3 SYPL2 CELF6 COL21A1 CXXC1 ADCY5 ZSCAN26 SRPK3 RP11-358D17.2 RP11-244F12.3 ESYT3 HOXB3 REC8 SGCB PPAPDC3 WHAMMP2 RASGRP2 TSC22D3 GPRASP2 CFH PINLYP TRO NRP2 CDH2 NACC2 RP11-513I15.6 ZNF382 HYI MROH7 CCDC102A ITGA1 WTIP CTD-3092A11.2 PRAF2 FLNC ARHGAP33 DPP10-AS1 RP5-890O3.9 MYOT CTD-3035K23.7 BCL2L2 SIPA1L2 CADM3-AS1 RCAN2 SCD5 ADGRL2 PLEKHG2 C4A MERTK COLGALT2 CORO2B CHGA DMPK PAN2 NEAT1 NUPR1 CTD-2517M22.14 MT1M RGAG4 TP53INP2 AKR1C2 RP11-793H13.10 ANTXR2 NPPA-AS1 MYL6 OLFM1 MAPT CASD1 LPPR2 IL1RL1 HOXD4 ZNF771 RASGEF1C TTBK2 CTXN1 MT-CYB CTC-296K1.4 SCRG1 TSHZ2 KIAA1755 4-Sep RDH5 ZNF540 HDAC9 DUSP26 RP11-887P2.5 TOM1L2 ZSCAN16-AS1 APLP1 NLGN2 RP11-504P24.3 AGAP9 RP11-231C14.4 TMEM252 FAM69B HECTD4 STRADA ZCCHC24 CTF1 MS4A6A EGFL7 RP4-669L17.10 IP6K3 GUCY1B3 NR3C1 RP11-1336O20.2 RP11-532F6.3 SCNN1D CHKB DNALI1 ASAP3 SMIM10 MT-ATP8 TCEA2 LA16c-390E6.4 PGM5-AS1 ACCS HDGFRP3 SH3BGRL PDXDC2P RP3-449O17.1 FAM149A PKD1L2 PPM1M ABCA8 PDE9A MAGOH2P PGBD1 RP5-1057I20.4 LINC00894 NBPF1 ILK BNIP3 STEAP4 FES RASA4 AOC3 CCNL2 NPTX2 WDR17 RP11-102K13.5 RP4-758J18.10 ANXA6 ZFHX4 LARGE P2RY1 TEAD2 TSPYL2 RP11-25K19.1 SNAP25 KRT24 BHMT2 COL6A2 FBLN1 FZD7 GATSL3 ANKS1B TMEM47 CHL1 LUC7L GATS PRKAB2 HSPB2 PGM5P4-AS1 DLG4 LINC01481 TTLL7 DEFB124 KLHDC8B SLX1A-SULT1A3 STOM DDX26B GSN ARL10 RAMP1 RAD51-AS1 TPTEP1 RP11-295P9.3 ST8SIA1 RSRP1 FRMD6 CNRIP1 MYH3 ANK2 SPON1 RAB23 PLA2G4A MAPRE3 CTD-2647L4.5 RIMS3 ZNF516 RP11-728F11.4 RP11-190A12.8 AVIL LMOD1 ZBTB16 ELN VIM GOLGA8A KCNMB1 DES RNF112 FGFR2 ASPA CERS1 NGB RP11-326G21.1 BEST1 REV3L RBM20 ZRANB1 RAD52 SLIT3 SDHAP1 FAM229B MAN1C1 HMP19 DISP1 LINC01160 RAB6B DENND4B AC025165.8 SCGN STK32A TPCN2 ST3GAL6 ATN1 MYH10 VPS9D1 TSSK3 TMEM220-AS1 C14orf159 HSF4 MYH11 MPP2 ZSWIM8 H1FX-AS1 MT-ND4 ITPR1 NICN1 NPAS4 DUSP1 FBXO17 NT5DC3 CPM MYO5A IRAK3 PARP6 RP5-902P8.10 MT-CO3 ENTHD2 CTRB2 AC009948.5 TBKBP1 GAMT GBP2 AC034220.3 AGPAT4 VWF NPIPA1 RPL24P8 TBX1 TPM1 CIDEC SALL2 EBF1 CD300LG CYP2E1 CRLF1 ABI3BP ATP1B2 NRG2 CTC-260F20.3 GABARAPL1 TIE1 TUB STK33 HSPA1A PIGCP1 VSIG4 AGTR1 WBSCR17 MCAM AC003973.3 CTD-3162L10.1 ZNF134 RIMKLB CRYBG3 USP2 OBSCN TNFAIP8L1 HRASLS5 CTD-2373N4.3 DPY19L2 CALY TNS1 PLCD4 MDM1 SMPX CDHR3 LINC00982 ZNF436-AS1 LENG8 PTX3 RP11-347I19.8 RP11-415J8.3 C3orf80 SEMA6A-AS1 SLC26A11 CTC-490E21.11 CCK RP11-486G15.2 PRUNE2 TRIM9 C8orf88 DFNB31 NXF1 HPGD SLC4A7 PLEKHH2 SEMA3B FKBP1B NSUN5P1 PER3 C10orf10 RP11-6O2.4 ADSSL1 PPP1R12C LRRTM1 GLI3 RP4-639F20.1 S100A1 RP11-430C7.5 RP5-1068E13.7 RBPMS2 TCEAL4 RP11-514P8.6 NFIC VPS13A SUN2 GPAT2 ASMTL-AS1 CELF3 NPIPB3 GRB14 MEIS3 MT1X MST1 NUMBL LARP6 CSPG4P12 JAZF1 SULT4A1 C7 WASF3 DYNC2H1 CCDC130 BEX4 ARRDC4 RAB31 CPQ PKD1P6 ANKRD9 MST1L MT-ND1 STX16-NPEPL1 ATG16L2 AP3B2 F8 LAMA5 ARHGAP23 LINC00106 RP11-350N15.4 ARNTL FUZ EHBP1 SEPT7P2 CCL2 TXNIP PRNP RP11-171I2.4 POPDC2 ZRANB2 C16orf86 CEP95 EFHC2 DFNA5 PDE1A PBX3 CAMK2G AC078941.1 CPED1 STAG3L5P ENKD1 C6orf3 FBXL2 CORO6 PRICKLE2 CMAHP PPT2-EGFL8 SEMA6A FAM131A AC005154.6 AC114730.11 TEF RP11-186B7.4 AC083843.1 FAM179A INAFM2 MTATP6P1 ADGRA2 TMEM100 RP11-499P20.2 UBAP1L MIR4697HG RP11-97C16.1 CACNA2D3 AC053503.12 CA7 ACSM5 PLIN1 SLC7A2 CLK1 C1orf95 TSSC2 FERMT2 PHLDB1 MAGEH1 ZNF333 RP11-13K12.1 RP11-1212A22.4 CPNE8 RP3-414A15.12 L1CAM AC017116.11 RGS2 TAGLN TPSAB1 TIGD7 CNTN3 GFRA3 CPA1 MOB3A RP11-326C3.7 DIRAS3 TMBIM4 RP11-20I20.4 RXRG PRKAA2 C1QTNF4 CD163 DPT RGS5 SORCS1 AP000892.6 PRDM8 PRKAR2B OGT RNF207 CCDC154 SLC8A1-AS1 MMP28 RPS2P7 FAM13C CCDC84 PDGFRL ADGRD1 ZDHHC11 MFAP4 SCAPER EDIL3 DENND5A PTPRS LATS2 SFRP5 ARHGEF37 TNC GUCA2B LEAP2 GADD45G RP11-575L7.8 PPP1R12B LURAP1 CD27-AS1 AC053503.6 PILRB CC2D2A RP4-742C19.13 PDE2A ZNF580 TUBB2A YPEL4 DUSP8P5 EZH1 LAYN RP11-203J24.9 MAGED4B MN1 RORA FBXL15 PLCG2 SLC22A17 EBF4 SCARA5 NFASC HTR2B CLCA4 MAOB CLDN5 ZNF512 PTGDS RP11-554A11.4 NCS1 ATP1A2 CCNL1 SCPEP1 ADGRB3 TGFB3 NAP1L3 RP11-380L11.4 RP5-966M1.6 AC006942.4 AC007326.1 ADAM33 PXDC1 BTC GPR183 RP11-867G23.1 SDPR LRRC24 LAMB1 LINC00899 NUDT11 AC005519.4 CTSG SNPH RP11-481J2.4 RARRES2 HOMER2 KLF8 ADAMTS1 PRKG1 ZNF354B CADM3 CLEC14A AC093642.3 CTC-510F12.7 PLA2G5 CYTL1 ACKR1 MTMR9LP SORBS1 ETFDH CTC-301O7.4 FGF7 RP11-932O9.9 LRCH2 COL28A1 MTND1P23 FNBP4 MB LIFR GSTT2B MT1L GTF2A1L ANXA8L1 MXI1 PGA3 NDST2 S1PR1 GIMAP7 TTC28 MAGI2 SGCA MTURN EVA1B NBPF10 MBNL1 SEMA6C GRIK5 ZFC3H1 DCUN1D4 SDK1 HAND1 PLCL1 RP11-802E16.3 LINC01355 CYS1 RP5-894A10.2 PTPN21 OSR1 ZNF302 GDPD3 ANKFY1 TBC1D1 AC016995.3 DENND3 SRP14-AS1 RP11-696N14.1 NPEPL1 TP53INP1 TUBB6 TSHZ3 ZNF300 CCDC80 RHOB FADS3 HEXDC RP11-359E10.1 FGF2 FXYD7 CLU PAMR1 EPHA6 CXCL12 TMEM198B NTN1 CNTNAP3 FCGR2C RP3-402G11.26 MT-ND3 EPHX1 LGI1 ADGRL3 AC026150.8 RP11-182J1.12 2-Mar LDB2 C16orf89 NECAB1 COL14A1 SNRPGP15 PRKD1 SOX8 CAPRIN2 LRRC4B EFEMP2 CSAD RECK CAV1 ALDH1A1 NLGN1 TMEM35 RAB3IL1 RAPGEF3 NAV1 RP11-44M6.7 SHISA9 BAG2 LINC00342 MICAL3 CYP1B1-AS1 SNRPN TTC21A SPARCL1 CTSL NEXN FOXF1 TPO IL6ST WNT2B CCT6P1 TTYH2 CASZ1 TCEAL5 EPM2A GPR146 U2AF1L4 PDZRN4 CARTPT ZNF528-AS1 MON2 TCIRG1 RP11-620J15.3 PPAP2B RP3-508I15.20 SIDT2 RASD1 DBN1 BEX1 SLC2A4 MIR600HG HOXA4 LINC01278 NBPF26 SLFN13 LY6H LINC01002 LYL1 ZNF34 MMP17 NPR2 MS4A4E KCNIP4 FLRT2 DYNC1I1 FLNB-AS1 DST TTLL3 ZEB2 METTL7A ITM2A CRYGS RP11-51B23.3 EIF4E3 CHRM2 RP11-159D12.2 MTCL1 COX7A1 RP11-498E2.9 CICP14 FAM189A2 RUSC2 CADM1 RP11-242D8.3 KLHL29 LILRB5 MAP9 GPRASP1 ACBD4 CXorf36 CDKN1C SPSB3 AQP1 ST6GALNAC6 RP4-635E18.8 RP11-64B16.2 AC159540.1 KIAA1614 CYP26B1 LYNX1 RGS9 TXLNGY AF001548.3 SUGP2 ANKRD12 RAB12 MS4A7 MPDZ PI4KAP2 CCL21 CEP126 RP11-92C4.6 ZNF626 FMO2 IL16 FAM189A1 TUBG2 VCL TTYH1 C3orf18 CCR10 LMO3 SOCS2 MAP1B TMEM220 RBMS3 CAP2 CUL9 DSEL STAT2 ACTG2 DPEP2 RP11-1277A3.2 DNAJB5 CREBRF MATN2 TMOD1 TDRP FAR2P2 ST6GALNAC2 MPP3 FGFBP2 ARHGEF6 RP11-412D9.4 CHRD LCN6 KLC1 RP11-228M15.1 INAFM1 NCALD RP11-166D19.1 USHBP1 WWTR1 RNF212 BEST2 ZNF337 RN7SL417P MEG3 RP4-631H13.6 RP3-331H24.7 BTBD19 AMOTL1 ADH1B ARHGAP6 RP5-855D21.3 ANGPTL2 KCTD7 RP11-66N24.4 ST3GAL3 KLF15 SCAND2P KB-1572G7.2 PTBP2 AMY2B PPP2R2B ARHGEF17 LA16c-349E10.1 CHL1-AS2 CCDC146 MT-CO2 GCG SCUBE2 SPRR3 RP13-131K19.6 PITPNM3 NRBP2 ULK2 RP11-72I8.1 AL442127.1 FABP4 ZNF667-AS1 KCNB1 RADIL IDUA CSRP2 RGS10 SLC25A28 CAB39L FSTL3 AC010226.4 PCP4 TTC23 RP11-304C12.5 NPTX1 PKDCC ECHDC2 CIRBP SGSM2 ACTA2-AS1 COL6A3 CTD-2537I9.18 RBM5-AS1 AC108488.4 NDUFA6-AS1 STARD9 EPHB6 RP11-106M3.2 PDLIM3 CRTAC1 CTB-133G6.1 PCSK5 FAM107A KLHL17 RGMA SAP25 GIMAP5 PAPLN RP11-574K11.32 CITED4 SLC25A12 RALGAPA1 GPX3 uc_338 TCP11L2 MALAT1 SIK3 PPP1R3C GPM6B MROH7-TTC4 ULK3 WDR86 MICALL2 ANK3 RP11-122K13.12 KRT5 FAM65C TIMP2 ITGB3 RP11-434B12.1 RP3-453C12.15 CLIP1 ABLIM3 MAP7D3 LTC4S TUBGCP6 LINC00578 ADAMTS10 FAM160B2 COL6A1 RP11-228B15.4 RP11-188P20.3 STMN4 AMT MIR497HG SGCE RP11-468E2.11 GPRC5B KITLG PDZD4 MMRN1 LINC01573 PI16 RP11-1379J22.2 GKAP1 FAM193B KDM5D RANP4 MIR22HG DDR2 RP11-1212A22.1 KCTD12 PKMP3 ACTA2 REM1 ZNF662 RN7SL608P RABL2A PTGS1 LAMA4 HOXD9 DOCK3 RP11-258C19.4 IGFBP4 CES4A ZNF266 SYNPO C1orf186 LINC00702 IGIP RP11-448G15.3 CASQ2 CLIC2 CTB-79E8.3 PYGM PRICKLE1 HES4 PEG10 WNT5B TPSD1 KB-68A7.1 SRSF5 TSNAXIP1 RP1-253P7.4 RP11-395I6.3 RP5-1142A6.9 SNED1 PLA2G4B TTN-AS1 RP13-516M14.1 C10orf54 KBTBD12 GGTA1P AHNAK2 DNER LPAR1 FOXD3-AS1 NKX3-2 EMILIN3 MAMDC2 TRPS1 ACOX2 VDAC1P8 POPDC3 GEM CA1 AC138035.2 MYL2 PTH1R RP11-408A13.4 AP001347.6 FAM110B ADAMTSL3 EVA1C CALHM2 PRRT2 PHYHD1 GAB1 CRISPLD2 FBXO32 SNHG14 PRSS53 TRNP1 HOXB2 GATM RUNDC3A RP11-175B9.3 CYP4F29P HDC HSPG2 AC018462.3 FLNA DPP10 CP AP000347.2 DAPK1 GALNT16 STAG3L5P-PVRIG2P-PILRB WHAMMP3 SERTAD4-AS1 TRIB2 INPP5A CES1 TNS2 CTC1 SRF ZNF300P1 C2orf74 TUBB4A SIN3B SEMA4C CDKL1 MTERF2 FMN2 RP4-565E6.1 NPTXR TTC7B DPYSL3 SCN9A RP11-53O19.1 SMYD1 UBE2Q2P1 ARMCX2 TCEAL1 ZNF358 MS4A14 RP11-1000B6.7 PHF21A FCHSD1 RP11-79D8.2 HLX PTGIS MDM4 PTCH2 FLRT1 HDAC6 CIART MAMLD1 ASB2 NDN FBLN5 HBA2 HCLS1 CCDC88A GPR15 LINC00891 RP4-800G7.2 LINC01004 ARNT2 GOLGA8B IL11RA NRSN2 HOXB-AS3 SPOCK1 DCAF8 HBA1 HOXA11-AS1_5 AQP7 |
