# Supplementary material for: Consequences of aberrated DNA methylation in Colon Adenocarcinoma: a bioinformatic-based multi-approach
Source: BMC Genom Data. 2022 Nov 29;23:83. doi: 10.1186/s12863-022-01100-7 (PMC9706923; doi:10.1186/s12863-022-01100-7)
Supplement: Supplementary file 4 — Additional file 4: Supplement 4. Methylation and Expression Data of CRC Cell Lines. [file 12863_2022_1100_MOESM4_ESM.docx]

**Supplement 4- Methylation and Expression Data of CRC Cell Lines**. The data was obtained from the DepMap database (<https://depmap.org/>). *HAND2* is hypermethylated in CRC cell lines, simultaneously downregulated. Furthermore, the Pearson correlation coefficient test revealed a negative correlation (Pearson R: -0.3035, p = 0.030) between the *HADN2* expression and methylation.

| Cell Lines | Expression (log2(TPM+1)) | Methylation (Beta-value) |
| --- | --- | --- |
| C2BBE1 | 0.056584 | 0.65871 |
| CCK81 | 0.014355 | 0.38108 |
| CL11 | 0.014355 | 0.85173 |
| CL14 | 0.275007 | 0.18858 |
| CL40 | 0.084064 | 0.42381 |
| COLO201 | 0.056584 | 0.6001 |
| COLO320 | 0.042644 | 0.85329 |
| COLO678 | 0.056584 | 0.90199 |
| CW2 | 0.014355 | 0.2968 |
| GP2D | 0 | 0.56075 |
| HCC56 | 0.014355 | 0.19867 |
| HCT116 | 0.056584 | 0.86303 |
| HCT15 | 0.124328 | 0.71016 |
| HT115 | 0 | 0.65847 |
| KM12 | 0 | 1 |
| LOVO | 0 | 0.45479 |
| LS123 | 0.070389 | 0.52005 |
| LS180 | 0.056584 | 0.21015 |
| LS411N | 0.014355 | 0.66816 |
| LS513 | 0.014355 | 0.42516 |
| MDST8 | 0.555816 | 0.3058 |
| NCIH508 | 0.014355 | 0.39411 |
| NCIH684 | 0.028569 | 0.65 |
| NCIH716 | 0 | 0.4242 |
| NCIH747 | 0 | 0.64673 |
| OUMS23 | 0.042644 | 0.16757 |
| RCM1 | 0.097611 | 0.14549 |
| RKO | 0 | 0.69236 |
| SKCO1 | 0 | 0.13748 |
| SNU1040 | 0.056584 | 0.0832 |
| SNU1197 | 0.137504 | 0.27504 |
| SNU175 | 0.286881 | 0.26043 |
| SNU283 | 0.15056 | 0.0424 |
| SNU407 | 0.014355 | 0.65265 |
| SNU503 | 1.803227 | 0.06932 |
| SNU61 | 0 | 0.15298 |
| SNU81 | 0 | 0.3294 |
| SNUC1 | 0.014355 | 0.22127 |
| SNUC2A | 0.400538 | 0.32072 |
| SNUC4 | 0.014355 | 0.32673 |
| SNUC5 | 0 | 0.89908 |
| SW1116 | 0.014355 | 0.47145 |
| SW1417 | 0 | 0.2437 |
| SW1463 | 0 | 0.56372 |
| SW403 | 0.097611 | 0.25461 |
| SW48 | 0 | 0.92294 |
| SW480 | 0 | 0.63297 |
| SW620 | 0 | 0.48567 |
| SW837 | 0 | 0.3385 |
| SW948 | 0 | 0.45846 |
| T84 | 0 | 0.28014 |
