# Supplementary material for: Consequences of aberrated DNA methylation in Colon Adenocarcinoma: a bioinformatic-based multi-approach
Source: BMC Genom Data. 2022 Nov 29;23:83. doi: 10.1186/s12863-022-01100-7 (PMC9706923; doi:10.1186/s12863-022-01100-7)
Supplement: Supplementary file 5 — Additional file 5: Supplement 5. The interactive biological process of the HAND2 target genes. [file 12863_2022_1100_MOESM5_ESM.docx]

**Supplement 5. The interactive biological process of the HAND2 target genes.**

| **Enrichment FDR** | **nGenes** | **Pathway Genes** | **Fold Enrichment** | **Pathway** | **URL** | **Genes** |
| --- | --- | --- | --- | --- | --- | --- |
| 0.048064 | 4 | 75 | 11.91948 | Neg. reg. of ERK1 and ERK2 cascade | http://amigo.geneontology.org/amigo/term/GO:0070373 | DAB2IP EMILIN1 CHRNA9 DMD |
| 0.02534 | 6 | 162 | 8.277415 | Pos. reg. of neuron projection development | http://amigo.geneontology.org/amigo/term/GO:0010976 | CBFA2T2 RAPGEF2 DAB2IP ELAVL4 BDNF DMD |
| 0.033789 | 7 | 274 | 5.709604 | Striated muscle cell differentiation | http://amigo.geneontology.org/amigo/term/GO:0051146 | FHL2 TNNT2 POPDC2 BDNF SLC8A1 ALPK2 DMD |
| 0.032568 | 8 | 350 | 5.108347 | ERK1 and ERK2 cascade | http://amigo.geneontology.org/amigo/term/GO:0070371 | RAPGEF2 DAB2IP EMILIN1 ITGAV P2RY6 CHRNA9 GPBAR1 DMD |
| 0.02534 | 10 | 453 | 4.933558 | Reg. of neuron projection development | http://amigo.geneontology.org/amigo/term/GO:0010975 | CBFA2T2 SEMA5B RAPGEF2 CHN1 DAB2IP TIAM2 THY1 ELAVL4 BDNF DMD |
| 0.033789 | 8 | 365 | 4.898415 | Pos. reg. of cell projection organization | http://amigo.geneontology.org/amigo/term/GO:0031346 | CBFA2T2 RAPGEF2 RAC2 DAB2IP TIAM2 ELAVL4 BDNF DMD |
| 0.02534 | 12 | 686 | 3.909449 | Neuron projection morphogenesis | http://amigo.geneontology.org/amigo/term/GO:0048812 | SEMA5B RAPGEF2 RAC2 CHN1 DAB2IP GFRA3 TIAM2 THY1 ELAVL4 GDNF BDNF DMD |
| 0.02534 | 14 | 815 | 3.839095 | Cellular component morphogenesis | http://amigo.geneontology.org/amigo/term/GO:0032989 | PHLDB1 SEMA5B RAPGEF2 TNNT2 RAC2 CHN1 DAB2IP GFRA3 TIAM2 THY1 ELAVL4 GDNF BDNF DMD |
| 0.02534 | 12 | 700 | 3.831261 | Plasma membrane bounded cell projection morphogenesis | http://amigo.geneontology.org/amigo/term/GO:0120039 | SEMA5B RAPGEF2 RAC2 CHN1 DAB2IP GFRA3 TIAM2 THY1 ELAVL4 GDNF BDNF DMD |
| 0.032568 | 11 | 644 | 3.817379 | Reg. of plasma membrane bounded cell projection organization | http://amigo.geneontology.org/amigo/term/GO:0120035 | CBFA2T2 SEMA5B RAPGEF2 RAC2 CHN1 DAB2IP TIAM2 THY1 ELAVL4 BDNF DMD |
| 0.02534 | 12 | 704 | 3.809492 | Cell projection morphogenesis | http://amigo.geneontology.org/amigo/term/GO:0048858 | SEMA5B RAPGEF2 RAC2 CHN1 DAB2IP GFRA3 TIAM2 THY1 ELAVL4 GDNF BDNF DMD |
| 0.02534 | 12 | 721 | 3.71967 | Cell part morphogenesis | http://amigo.geneontology.org/amigo/term/GO:0032990 | SEMA5B RAPGEF2 RAC2 CHN1 DAB2IP GFRA3 TIAM2 THY1 ELAVL4 GDNF BDNF DMD |
| 0.032568 | 12 | 775 | 3.460493 | Cell morphogenesis involved in differentiation | http://amigo.geneontology.org/amigo/term/GO:0000904 | SEMA5B RAPGEF2 RAC2 CHN1 ITGAV GFRA3 TIAM2 THY1 ELAVL4 GDNF BDNF MYADM |
| 0.02534 | 15 | 1085 | 3.089726 | Cell morphogenesis | http://amigo.geneontology.org/amigo/term/GO:0000902 | SEMA5B RAPGEF2 RAC2 CHN1 DAB2IP ITGAV GFRA3 TIAM2 THY1 ELAVL4 GDNF BDNF MYADM CDC42SE1 DMD |
| 0.033789 | 14 | 1034 | 3.025979 | Reg. of anatomical structure morphogenesis | http://amigo.geneontology.org/amigo/term/GO:0022603 | PHLDB1 SEMA5B LFNG RAPGEF2 RAC2 CHN1 DAB2IP EMILIN1 TIAM2 THY1 GDNF BDNF MYADM CDC42SE1 |
