# Supplementary material for: Consequences of aberrated DNA methylation in Colon Adenocarcinoma: a bioinformatic-based multi-approach
Source: BMC Genom Data. 2022 Nov 29;23:83. doi: 10.1186/s12863-022-01100-7 (PMC9706923; doi:10.1186/s12863-022-01100-7)
Supplement: Supplementary file 6 — Additional file 6: Supplementary Figure 1. Hypermethylated Regions of HAND2 CpG Islands and Their Correlation with HADN2 Expression in CRC Samples Obtained from SMART Database. [file 12863_2022_1100_MOESM6_ESM.docx]

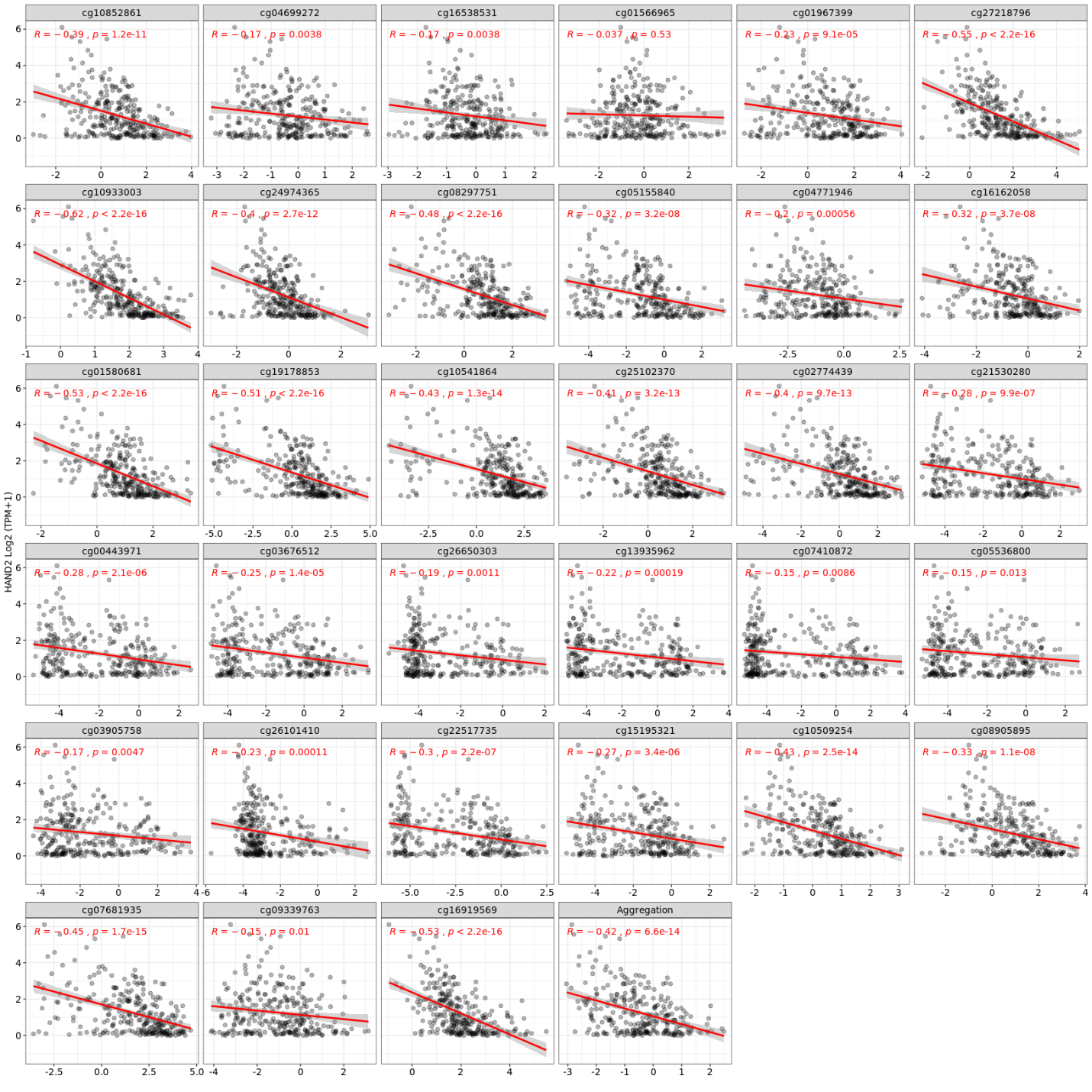


**Supplementary Figure 1. Hypermethylated Regions of HAND2 CpG Islands and Their Correlation with HADN2 Expression in CRC Samples Obtained from SMART Database.** The methylated data is expressed with M-value, and expression data is based on _log2_(TPM+1). The aggregation of all methylated regions compared to expression revealed that *HAND2* significantly (R = -0.44, p= 6.6e-14 for colon cancer) hypermethylated and downregulated colon adenocarcinoma. The aggregation method is expressed as the mean of thirty-three founded probs for HAND2. Correlation between gene expression and methylation (Number of samples in COAD: 288), COAD: Colon adenocarcinoma.
