# Supplementary material for: Consequences of aberrated DNA methylation in Colon Adenocarcinoma: a bioinformatic-based multi-approach
Source: BMC Genom Data. 2022 Nov 29;23:83. doi: 10.1186/s12863-022-01100-7 (PMC9706923; doi:10.1186/s12863-022-01100-7)
Supplement: Supplementary file 8 — Additional file 8: Supplementary Figure 3. Promoter methylation is correlated with gene expression in CRC cell lines. [file 12863_2022_1100_MOESM8_ESM.docx]

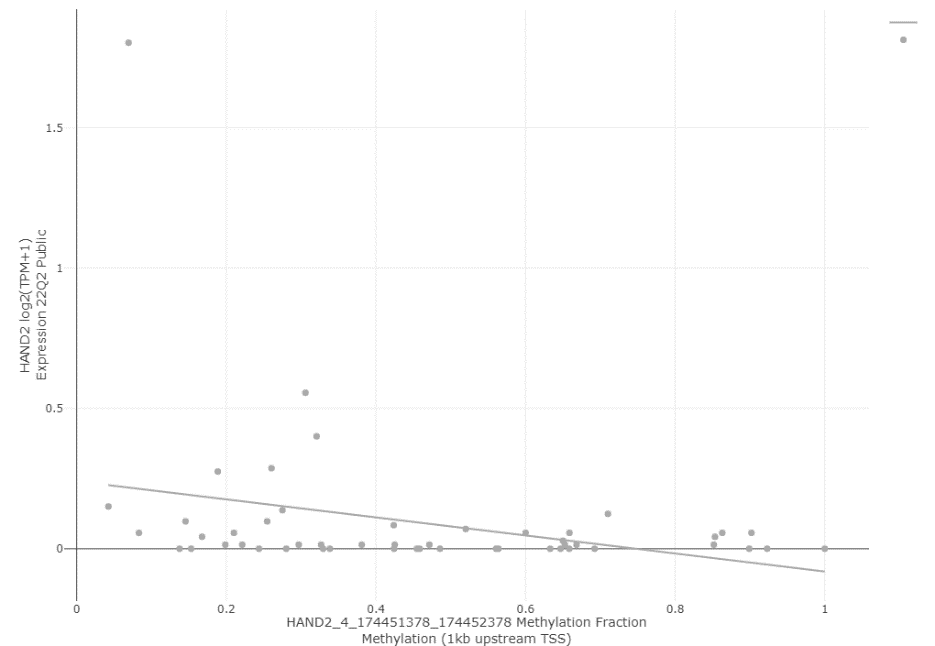


**Supplementary Figure 3. Promoter methylation is correlated with gene expression in CRC cell lines.** Scatter-plot depicts the correlation between changes in promoter methylation and HAND2 expression in CRCs (Pearson's correlation coefficient= -0.3035), indicating increased promoter methylation is associated with HAND2 downregulation. The figure is depicted by https://depmap.org/.
